# Regression without regrets –initial data analysis is a prerequisite for multivariable regression

**DOI:** 10.1186/s12874-024-02294-3

**Published:** 2024-08-08

**Authors:** Georg Heinze, Mark Baillie, Lara Lusa, Willi Sauerbrei, Carsten Oliver Schmidt, Frank E. Harrell, Marianne Huebner, on behalf of TG2 and TG3 of the STRATOS initiative

**Affiliations:** 1 Center for Medical Data Science, Institute of Clinical Biometrics, Medical University of Vienna, Spitalgasse 23, 1090 Vienna, Austria; 2 Novartis Pharma AG, Basel, Switzerland; 3 Faculty of Mathematics, Department of Mathematics, University of Primorska, Natural Sciences and Information Technology, Koper, Slovenia; 4 Faculty of Medicine, Institute of Biostatistics and Medical Informatics, University of Ljubljana, Ljubljana, Slovenia; 5 Faculty of Medicine and Medical Center, Institute of Medical Biometry and Statistics, University of Freiburg, Freiburg, Germany; 6 Institute of Community Medicine, University Medicine of Greifswald, SHIP-KEF, Greifswald, Germany; 7 School of Medicine, Department of Biostatistics, Vanderbilt University, Nashville, TN USA; 8 Department of Statistics and Probability, Michigan State University, East Lansing, MI USA

**Keywords:** Initial data analysis, IDA framework, Regression models, Data screening, Reporting, Variable selection, Functional form, Variable transformation, STRATOS Initiative

## Abstract

Statistical regression models are used for predicting outcomes based on the values of some predictor variables or for describing the association of an outcome with predictors. With a data set at hand, a regression model can be easily fit with standard software packages. This bears the risk that data analysts may rush to perform sophisticated analyses without sufficient knowledge of basic properties, associations in and errors of their data, leading to wrong interpretation and presentation of the modeling results that lacks clarity. Ignorance about special features of the data such as redundancies or particular distributions may even invalidate the chosen analysis strategy. Initial data analysis (IDA) is prerequisite to regression analyses as it provides knowledge about the data needed to confirm the appropriateness of or to refine a chosen model building strategy, to interpret the modeling results correctly, and to guide the presentation of modeling results. In order to facilitate reproducibility, IDA needs to be preplanned, an IDA plan should be included in the general statistical analysis plan of a research project, and results should be well documented. Biased statistical inference of the final regression model can be minimized if IDA abstains from evaluating associations of outcome and predictors, a key principle of IDA. We give advice on which aspects to consider in an IDA plan for data screening in the context of regression modeling to supplement the statistical analysis plan. We illustrate this IDA plan for data screening in an example of a typical diagnostic modeling project and give recommendations for data visualizations.

**Supplementary Information:**

The online version contains supplementary material available at 10.1186/s12874-024-02294-3.

## Introduction

Statistical models are commonly used in medicine and other fields, e.g., for predicting an outcome variable based on the values of some predictor variables, for describing the association between the outcome and the predictors, or for estimating the causal effect of an intervention on an outcome, adjusted for covariates. This is often done by defining a model structure such as, in its simplest form, a linear combination of the covariates, and estimating the unknown parameters of the model. Many aspects of multivariable regression analyses such as choosing an appropriate model family, covariate selection for a model, consideration of nonlinear associations of continuous covariates with the outcome, or validation of regression models have been discussed extensively [1–4]. However, correct interpretation and adequate presentation of a model crucially depend on knowledge about the predictors, in particular about their marginal and joint distributions. Further properties of the data such as patterns of missing values, collinearities, measurement errors, or complex hierarchies in the measured predictors may have to be considered when choosing an appropriate model building strategy.

In practice, however, with standard software packages and coded functions for statistical procedures enabling regression analyses with little effort, data analysts may rush to perform sophisticated analyses, without systematically checking for errors in the data; without a clear understanding about the underlying features of the data; without knowledge on the suitability of the data for the intended analyses, or even without knowledge whether the data actually could provide answers to the research questions of interest. This problem may be even more prevalent when machine learning algorithms are used to model the data, because anomalies in the results are not so easily detected due to the lower explainability of these models. Our previous review of initial data reporting in five highly ranked medical journals revealed that some sort of data screening was performed in all papers and led to amendments of the intended statistical analysis [5]. It remained unclear if these screening steps and subsequent amendments were preplanned or performed in a post-hoc fashion and the reporting was poor [5].

The main aim of Initial Data Analysis (IDA) is seen in providing reliable and transparent information about the data and how they meet preconditions to conduct appropriate statistical analyses and a correct interpretation of the results to answer pre-defined research questions [6]. Others have noted the need for a strategic approach to initial data analysis (for example, [7]). The IDA framework consists of six steps [6] incorporated in the research work flow [8]. Regarding steps I and II of the framework, we assume that metadata exist in sufficient detail, and that data cleaning was already performed [9]. Metadata summarize background information about the data, and a data cleaning process identifies and corrects technical errors. In this paper we focus on those aspects of the IDA framework that address preparation of the data for building a regression model and possible consequences of the findings (steps III—VI).

- Data screening examines data properties to inform decisions about the intended analysis.
- Initial data reporting documents give insights into the previous steps and can be referred to when interpreting results from the regression modeling.
- Possible consequences of such initial analyses may be that the intended way of building the regression models may have to be revised, i.e., the analysis plan refined or updated.
- Finally, reporting of IDA methods and results in research papers (step VI) is necessary to ensure transparency regarding key findings that influence the analysis or interpretation of results.

Further details about the elements of IDA are discussed in [6].

Our objectives are (1) to give advice on what to consider in an IDA plan for data screening in the context of regression modeling to supplement the statistical analysis plan and (2) to illustrate this IDA plan for data screening in an example with recommendations for data visualizations. While our examples are taken from medicine, our recommendations are not meant to be restricted to medical applications but may also guide data analysts in other fields of science. Reproducible R code with link to dataset is available at https://stratosida.github.io/regression-regrets/. Intermediate results of this project have been presented at various conferences and workshops. Feedback was sought from STRATOS members and other experienced statisticians to come to a consensus.

We outline the assumptions made in our paper in Sect. "Aims and scope of initial data analysis in the context of regression analysis". In Sect. "General strategy to develop an IDA plan for regression analyses" we define a list of several topics of relevance for an IDA plan in a regression modeling context. We show how to prespecify IDA topics by means of an example study in Sect. "Illustrative example: bacteremia study". In Sect. "Possible consequences of IDA", we discuss possible consequences of IDA findings. Finally, we reflect on integrating IDA in the research process for regression analyses and address reporting and limitations.

## Aims and scope of initial data analysis in the context of regression analysis

This paper elaborates on planning and conducting IDA, in particular data screening, in a reproducible manner in the context of regression analyses with a continuous, binary, ordinal or count-type outcome variable. While we focus on descriptive or predictive research questions, many aspects discussed here will also extend to explanatory models that seek to estimate a causal effect of an intervention. We are very aware of the danger of formulating and testing hypotheses after exploring the data that can lead to overstated associations and false positive results [10, 11]. Hence, IDA should not be confused with exploratory data analysis (EDA) or with Chatfield’s notion of an ‘initial examination of data’, as the latter two approaches explicitly evaluate associations of the outcome and the predictors [12, 13]. Instead a key principle of IDA is that it should –without good reason- not anticipate analysis directly related to the research question, implying that associations between outcome and predictors are not explored, neither numerically nor graphically. Nevertheless, the conduct of IDA is guided by the research question and the intended analyses.

**Fig. 1:**
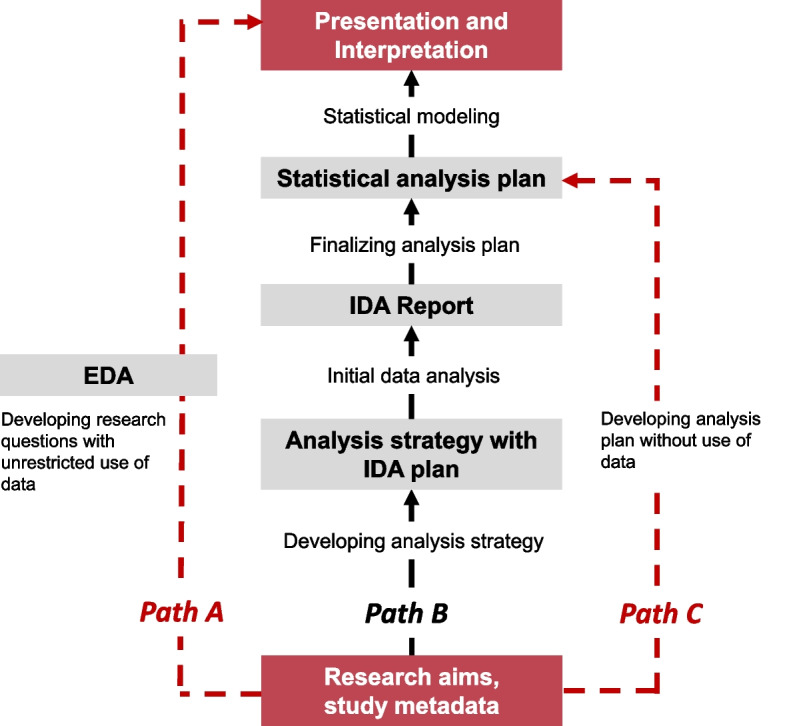
Embedding of initial data analysis (IDA) in a research workflow (Path B), contrasted to two extremes: a workflow based on exploratory data analysis (EDA) that has unrestricted access to the data when developing research questions and analysis strategy (Path A) and a strategy that does not make use of any initial data analysis to fully prespecify the statistical analysis plan (Path C)

Figure 1 illustrates how IDA planning and conducting could be embedded in the research workflow, and contrasts the research flow including IDA (Path B) to workflows that unrestrictedly use the data to develop research questions and the analysis strategy (EDA, Path A), and to a workflow that fully prespecifies statistical analysis without initial data analysis (Path C). Ideally, a statistical analysis plan can be fully prespecified before data collection, and it may entail, among many other items, the specification of the predictor set to be included in a model, the model structure, the outcome variable, any further steps considered in model building, and specifications how the modeling results will be interpreted with respect to the research question, and how they will be presented. This statistical analysis plan may also contain adaptive specifications since it is often not clear upfront if certain assumptions are justified by the data. Next, an IDA plan is devised which specifies a set of (mostly descriptive) analyses of the data to be conducted before the prespecified regression model is estimated, carefully avoiding any evaluations of predictor-outcome associations. This IDA plan may also include evaluating conditions that lead to changes in the prespecified analysis strategy, e.g. because of abundance of missing values, very imbalanced or even degenerate distributions or because of redundancy of predictors. Then IDA is conducted according to the prespecified IDA plan. Using the results of IDA, the statistical analysis plan may be updated or refined; any changes will be transparently reported as a consequence of IDA. Finally, statistical analysis is conducted and reported using the finalized statistical analysis plan. Besides suggesting refinements or updates of a preliminary statistical analysis plan, IDA may also guide presentation and interpretation of modeling results, which will be exemplified in later sections of the paper.

As a simple example, consider the case where the prespecified model includes a particular categorical predictor with four levels, which should be included as three dummy variables contrasting three levels to a common predefined reference level. There is hardly any background knowledge about the expected frequencies of the four levels. Without consideration of IDA, during the regression modeling process it may turn out that the prespecified model is not estimable because at one of the levels of the predictor no events were observed. The analysis team may recommend to revise the analysis strategy and to collapse this level with other levels in the analysis, but such revision is problematic because it is made post-hoc and has not been part of the analysis plan; critical assessors of the research may be suspecious about the criteria upon which the choice to collapse the levels was made, as the analysts may have tried several different analyses. Let’s assume that the IDA plan included checks of the distributions of each predictor and conditions under which collapsing of predictor levels is considered. IDA was then conducted according to this plan and revealed the sparsity of one level of the predictor, but without evaluating the frequency of outcome events for that level. As a result, the statistical analysis plan was refined according to the predefined conditions, transparently stating that the sparse level of the categorical predictor in question was collapsed with a suitable other level. Final statistical modeling proceeded according to the pre-specified, finalized SAP.

STROBE [15] and TRIPOD [16] are reporting guidelines giving advice on which aspects of a study to report. TARMOS [17] is a recently proposed framework for treatment and reporting of missing values in observational studies. In these frameworks, a couple of items/statements relate to description of study data. Our systematic approach to initial data analysis is meant to provide the information that the modeling team needs; it is not meant to create results that should directly enter the final report, although some of them will. Since regression models can be used for a wide range of purposes, assumptions on the regression analysis set-up in this paper are listed in Table 1. IDA tasks will be explained in a well-defined, practically relevant setting typically encountered in biomedical research.

**Table 1:** Assumptions and scope for a general strategy of initial data analyses as prerequisite of regression analyses

Aspect	Details
Purpose of analysis	Focus on descriptive or predictive research aims; many aspects also apply to causal aims
Type of analysis	Regression analysis with one outcome variable and several predictors
Type of outcome variable	Continuous, binary or count
Number of predictors	3 – 50; the number is assumed smaller than the number of effective observations (low-dimensional setting)
Data cleaning	Has been conducted; for example, plausibility limits of all variables satisfied
Analysis strategy	Most elements defined: type of regression model, set of candidate predictors, selection of variables, consideration of possible nonlinear effects of continuous predictors, coding of categorical variables, inclusion of interactions, missing data handling
Background knowledge	Data analyst collaborates with a domain expert to discuss all aspects of the analysis strategy

## General strategy to develop an IDA plan for regression analyses

In this section, we provide guidance on how to develop an IDA plan focused on data screening to prepare regression analyses (Table 2) and is focused on three IDA domains: missing values, univariate distributions, and the multivariate system of predictors. We explain the aspects that we consider mandatory to be addressed in an IDA plan, and refer the reader to Additional file 1 for prerequisites (Section S1) and optional extensions (Section S2). Consequences of IDA results are discussed in Sect. "Possible consequences of IDA".

**Table 2:** Check list for an initial data analysis (IDA) plan

Topic	Item	Features
**Prerequisites**		
Research aim	PRE1	Define the research aim (descriptive, predictive, or causal) and phase of research (exploratory or confirmatory)
Analysis strategy	PRE2	Check specification of models and roles of variables in the models
Data dictionary	PRE3	For variables identified in PRE2, and any additional structural variables, check variable labels, definitions, values, units of measurement, data type, etc
Domain expertise	PRE4	When discussing analysis strategy with a domain expert, address: key predictors, structural variables for IDA, predictor grouping, expected missing values proportion, and predictor distributions/correlations
**IDA screening domain: Missing values (predictor and outcome variables)**		
Participant (unit) missingness	M1	Describe: number of potentially eligible but not assessed, assessed but not recruited, and recruited but didn’t contribute data
Variable (item) missingness	M2	Provide missing value count and proportion for each predictor and the outcome variable. Distinguish by reason, if applicable
Complete cases	M3	Describe complete observations for outcome and predictors in any model described in PRE2
Patterns	M4	Investigate missing value patterns across all variables, structured by structural variables. Display as tables or appropriate visualizations
Missing values – Optional extensions		
Predictors	ME1	Investigate predictors of missingness (complete vs incomplete cases)
**IDA screening domain: Univariate descriptions (structural variables, predictors and outcome)**		
Categorical variables	U1	Summarize category frequencies and proportions, with appropriate plots. Summarize frequencies of collapsed categories as well
Continuous variables	U2	Inspect distributions with high-resolution histogram, summary of key quantiles (e.g. 1st, 5th, 25th, 50th, 75th, 90th, 99th) extreme values (5 highest and 5 lowest), measures of central tendency (mean) and dispersion (Gini mean difference, standard deviation, interquartile range). Include number of distinct values. Describe mode of the data and its frequency. Similarly, inspect distributions of transformed variables, if applicable
Univariate analyses – Optional extensions		
Sparsity	UE1	Create distributional plots to identify observations with extreme values
**IDA screening domain: Multivariate descriptions (structural variables and predictors)**		
Association	V1	Visualize and summarize the association of each predictor with the structural variables
Correlation	V2	Quantify association (e.g., pairwise correlations) between all key predictors in a matrix or heatmap
Interactions, if applicable	V3	Evaluate bivariate distributions of the predictors specified in interactions, incorporating appropriate graphical displays
Multivariate analyses – Optional extensions		
Correlation	VE1	Compare results from different association metrics
Clustering	VE2	Visualize clustering of predictors using a dendrogram to show closely associated predictors
Redundancy	VE3	Compute Variance Inflation Factors or fit parametric additive models to assess the predictability of each predictor from the remaining predictors

### Missing data

In many studies, missing values are a central and dominant problem which needs to be addressed. We propose to start data screening with missing values, first evaluating various levels of unit missingness (Table 2, M1) as recommended by the STROBE statement [15]. Unit missingness refers to observational units that have missing data on all variables required for analysis. In observational studies, this means that parts of the target population are (randomly or not randomly) underrepresented in the study cohort. Unit missingness could result from a biased selection process that may distort results (Table 2, ME1).

The proportion of missing values (item missingness) should be computed for the outcome and separately for each predictor (Table 2, M2). This is then followed by evaluating the number of complete observations that are available for a regression model (Table 2, M3). Information about patterns of missing values in the data may be useful for the modeling team to decide on possible substitution of predictors with abundant missing values. If stratified by the structural variables, such patterns may give further information about missingness mechanisms; for example, if missingness of a predictor is associated with time of recruitment this may indicate that measurements of this predictor became available only during the course of a study (Table 2, M4).

**Table 3:** Simplified data dictionary of the bacteremia study. Key predictors and structural variables are in boldface, predictors of medium importance in italic

Variable	Label	Scale	Units	Variable	Label	Scale	Units
ID	Patient Identification	nom	1–14,691	GBIL	Bilirubin	cont	mg/dl
BloodCulture	Blood culture result for bacteremia	nom	no, yes	GLU	Glucoses	cont	mg/dl
**AGE**	**Patient Age**	**cont**	**years**	HCT	Haematocrit	cont	%
**BUN**	**Blood urea nitrogen**	**cont**	**mg/dl**	HGB	Haemoglobin	cont	G/L
**CREA**	**Creatinine**	**cont**	**mg/dl**	HS	Uric acid	cont	mg/dl
**NEU**	**Neutrophiles**	**cont**	**G/L**	LDH	Lactate dehydrogenase	cont	U/L
**PLT**	**Blood platelets**	**cont**	**G/L**	LIP	Lipases	cont	U/L
**SEX**	**Patient sex**	**nom**	**1 = male, 2 = female**	LYM	Lymphocytes	cont	G/L
**WBC**	**White blood count**	**cont**	**G/L**	LYMR	Lymphocyte ratio	cont	% (mg/dl)
*ALAT*	*Alanin transaminase*	*cont*	*U/L*	MCH	Mean corpuscular hemoglobin	cont	fl
*ASAT*	*Aspartate transaminase*	*cont*	*U/L*	MCHC	Mean corpuscular hemoglobin concentration	cont	g/dl
*CRP*	*C-reactive protein*	*cont*	*mg/dl*	MCV	Mean corpuscular volume	cont	pg
*GGT*	*Gamma-glutamyl transpeptidase*	*cont*	*G/L*	MG	Magnesium	cont	mmol/L
*FIB*	*Fibrinogen*	*cont*	*mg/dl*	MONO	Monocytes	cont	G/L
*POTASS*	*Potassium*	*cont*	*mmol/L*	MONOR	Monocyte ratio	cont	%
ALB	Albumin	cont	G/L	MPV	Mean platelet volume	cont	fl
AMY	Amylase	cont	U/L	SODIUM	Sodium	cont	mmol/L
AP	Alkaline phosphatase	cont	U/L	NEUR	Neutrophile ratio	cont	%
APTT	Activated partial thromboplastin time	cont	sec	NT	Normotest	cont	%
BASO	Basophiles	cont	G/L	PAMY	Pancreas amylase	cont	U/L
BASOR	Basophile ratio	cont	%	PDW	Platelet distribution width	cont	%
CA	Calcium	cont	mmol/L	PHOS	Phosphate	cont	mmol/L
CHE	Cholinesterase	cont	kU/L	RBC	Red blood count	cont	T/L
CHOL	Cholesterol	cont	mg/dl	RDW	Red blood cell distribution width	cont	%
CK	Creatinine kinases	cont	U/L	TP	Total protein	cont	G/L
EOS	Eosinophils	cont	G/L	TRIG	Triclyceride	cont	mg/dl
EOSR	Eosinophil ratio	cont	%				

### Univariate descriptions

Information about the empirical univariate distributions of the outcome and the involved predictors in the data is important for later modeling decisions, for presentation of modeling results, and also for interpreting a model correctly (Table 2, U1 & U2). Such analyses may also detect concentrations of data, e.g., the distribution of distinct values, a spike at zero or digit preferences depending on the relative frequency. This information may guide the decision whether the functional form of a continuous predictor can be modelled flexibly, which would require a sufficient number of observations in the area where a nonlinear association with the outcome is expected, or should be better predefined, e.g., as linear. It may also guide a strategy to deal with influential data points, such as truncation or transformation of a predictor. For the purpose of presenting the modeling results, the univariate distribution of a predictor may guide the choice of appropriate units corresponding to the regression coefficients.

### Multivariate descriptions

Even an only moderate number of predictors can be challenging for concise multivariate descriptions. Therefore, we propose the following structured approach to limit the number of descriptions to be produced at this analysis stage. First, associations of each predictor with the structural variables should be evaluated graphically and numerically (Table 2, V1). These analyses help to understand how the predictors reflect structural heterogeneity. Next, associations between all pairs predictors should be assessed, and these analyses could be restricted to numerical evaluation if the number of predictors is large (Table 2, V2). In case of missing predictor values, it may be sufficient to use all pairwise complete observations to compute association measures between predictors. If nonlinear functional forms are considered for continuous predictors in a regression model, nonparametric (Spearman) correlation coefficients are a good choice. Other types of variables, e.g., categorical ones, may require other measures of association. If the analysis strategy prespecified the consideration of some biologically plausible interactions, the association of the involved predictors should be given special attention in IDA, as high correlation between them may influence the ability to detect their interaction (Table 2, V3).

We emphasize again that these multivariate analyses should not incorporate the outcome variable to avoid hypothesis generation activities.

## Illustrative example: bacteremia study

To illustrate the IDA plan (Table 2) an example study in a regression context with publicly available data and R code will be shown. The corresponding IDA plan, detailed analyses, and materials can be found in Additional file 2 and at the accompanying website (see Availability of data and materials). In this section, we chose a subset from this comprehensive material to exemplify the key aspects. Possible consequences are discussed in Sect. "Possible consequences of IDA".

### Overview of the bacteremia study

We will exemplify our proposed systematic approach to data screening by means of a diagnostic study with the aim to fit a diagnostic prediction model for the bacteremia status (= presence or absense of bacteria in the blood stream) of a blood sample. While blood culture analysis is the gold standard for identifying bacteremia in patients with suspected blood stream infection, true positive rates and false positive rates have been reported to be in similar ranges (4.1%-7% and 0.6%- 8%, respectively) [18]. Moreover, blood culture analysis usually takes a few days to complete but is needed to inform the decision whether to initiate antibiotic treatment or not. A quick and precise estimate of the pretest probability of a positive finding of the blood culture analysis based on routinely available measurements may help to avoid unnecessary antibiotic treatment and may increase the cost-effectiveness of blood culture analysis. Hence, the hypothetical primary objective of our example study is to use age, sex and 49 routinely collected laboratory variables to fit a diagnostic prediction model for blood-culture confirmed bacteremia. The hypothetical secondary objective is to describe the functional form of each predictor in the model, which is helpful for model explanation. Between January 2006 and December 2010, patients from the General Hospital of Vienna, Austria, with the clinical suspicion to suffer from bacteremia were included into this study if blood culture analysis was requested by the responsible physician and blood was sampled for assessment of hematology and biochemistry. An analysis of this study can be found in Ratzinger et al. [18].

The data consists of 14,691 observations from different patients among which 8% had bacteremia and 51 potential predictors. To protect data privacy our version of this data was slightly modified compared to the original version, and this modified version was cleared by the Medical University of Vienna for public use (DC 2019–0054). Compared to the official results given in [18], our results may differ to a negligible degree.

### Bacteremia study: prerequisites for the IDA plan

#### Research aim (PRE1)

We assume that the aims of the study are to fit a diagnostic prediction model for bacteremia with 51 potential predictors collected in routine laboratory analyses of blood sampled and to describe the functional form of each predictor. These research aims are considered as exploratory.

#### Analysis strategy (PRE2)

These aims are addressed by fitting a logistic regression model with bacteremia status as the dependent variable. Prediction models for bacteremia that preceded the model of Ratzinger et al. [18] (see the citations therein) included the predictors age (AGE), leukocytes (WBC), blood urea neutrogen (BUN), creatinine (CREA), thrombocytes (PLT), and neutrophiles (NEU). Hence, we consider these variables are key predictors with known strong associations with bacteremia. Upon consultation of a laboratory medicine specialist, some variables were considered as of medium importance to predict bacteremia: potassium (POTASS) (which is related to kidney function), and some acute-phase related parameters such as fibrinogen (FIB), C-reactive protein (CRP), aspartate transaminase (ASAT), alanine transaminase (ALAT), and gamma-glutamyl transpeptidase (GGT). All other potential predictors are probably of minor importance. Therefore, the three candidate models are based on the key predictors only, based on key predictors and predictors of medium importance, and based on all predictors. Continuous predictors should be modelled by allowing for flexible functional forms. Since there is a large number of potential predictors, the flexibility of the functional form of a predictor (which determines the number of parameters in the model) will follow its assumed importance for predicting bacteremia. Hence, more flexibility will be allowed for key predictors (e.g., to represent at least cubic associations with the outcome) than for all other predictors (e.g., enabling the modeling of quadratic associations). The decision on which candidate model to use will be made based on results of data screening before uncovering the association of predictors with the outcome variable. In this example, an adequate strategy to cope with missing values will also be chosen after screening the data. Candidate strategies are omission of predictors with abundant missing values, complete case analysis, single value imputation or multiple imputation with chained equations, or a combination of those. In other types of studies, in particular longitudinal studies or studies with few candidate predictors, one might be forced or able to prespecify handling of missing data if sufficient prior knowledge about missingness patterns is available [19].

#### Data dictionary (PRE3)

The data dictionary of the bacteremia data set consists of columns for variable names, variable labels, scale of measurement (continuous or categorical), units, plausibility limits, and remarks (a simplified version is in Table 3). In the original data dictionary the variables are sorted by alphabetical order, but for Table 3 we sorted them by importance.

#### Domain expertise (PRE4)

The demographic variables age and sex are chosen as the structural variables in this analysis for illustration purposes, since they are commonly considered important for describing a cohort in health studies. Key predictors and predictors of medium importance are as defined above. Laboratory analyses always bear the risk of machine failures, and hence missing values are a frequent challenge. This may differ between laboratory variables, but no a priori estimate about the expected proportion of missing values can be assumed. As most predictors measure concentrations of chemical compounds or cell counts, skewed distributions are expected. Some predictors describe related types of cells or chemical compounds, and hence some correlation between them is to be expected. For example, leukocytes consist of five different types of blood cells (BASO, EOS, NEU, LYM and MONO), and the sum of the concentration of these types approximately (but not exactly) gives the leukocyte count, which is recorded in the variable WBC. Moreover, these variables are given as absolute counts and as percentages of the sum of the five variables, which creates some correlation. Some laboratory variables differ by sex and age, but the special selection of patients for this study (suspicion of bacteremia) may distort or alter the expected correlations with sex and age.

### Bacteremia study: IDA plan

In the following, we exemplify an IDA plan for the bacteremia study which uses the template of Table 2. The plan is written in future tense as we assume it is created before looking into the data.

#### Participant missingness (M1)

As the data is exported from the registry of the laboratory, and only performed laboratory analyses are included, participant missingness cannot be evaluated.

#### Variable missingness (M2)

Numbers and proportions of missing values will be reported for each predictor and the outcome separately (M2). Type of missingness has not been recorded.

#### Complete cases (M3)

The number of available complete cases (outcome and predictors) will be reported when considering:

1. outcome
2. outcome and structural variables,
3. outcome and key predictors only,
4. outcome and key predictors and predictors of medium importance,
5. outcome and all predictors.

#### Patterns of missing values (M4)

Patterns of missing values will be investigated by:

1. computing a table of complete cases (see 4.3.3) for strata defined by the structural variables age and sex,
2. constructing a dendrogram of missingness indicators to explore which predictors tend to be missing together.

#### Univariate descriptions: Categorical variables (U1)

For sex and bacteremia status, the frequency and proportion of each category will be described.

#### Univariate descriptions: Continuous variables (U2)

For all continuous predictors, combo plots consisting of high-resolution histograms, boxplots and dotplots will be created. Because of the expected skewed distribution, combo plots will also be created for log-transformed predictors. As numerical summaries, minimum and maximum values, main quantiles (5th, 10th, 25th, 50th, 75th, 90th, 95th), the mean, the Gini mean difference, the number of distinct values, and the five lowest and five highest values will be reported.

Graphical and parametric multivariate analyses of the predictor space such as cluster analyses or the computation of variance inflation factors can be heavily influenced by the distribution of the predictors. In order to make this set of analyses more robust to highly influential points or areas of the predictor support, some predictors may need transformation (e.g. cube root or logarithmic transformation). As possible transformations we will consider cube roots and logarithms of predictors. Since some predictors may have values at or close to 0, we will consider the pseudolog transformation instead of the log transformation [20]. The success of transformations to symmetrize predictor distributions will be assessed by evaluating each untransformed and transformed predictor’s correlation with normal deviates. Additional file 2 Appendix A contains some further explanations on the pseudo-log transformation.

#### Multivariate descriptions: associations of predictors with structural variables (V1)

A scatterplot of each predictor with age, with different panels for males and females will be constructed. Associated Spearman correlation coefficients will be computed.

#### Multivariate descriptions: correlation analyses (V2)

A matrix of Spearman correlation coefficients will be computed.

#### Comparing nonparametric and parametric predictor correlation (VE1)

A matrix of Pearson correlation coefficients will be computed. Predictor pairs for which Spearman and Pearson correlation coefficients differ by more than 0.1 correlation units will be depicted in scatterplots.

#### Variable clustering (VE2)

A variable clustering analysis will be performed to evaluate which predictors are closely associated. A dendrogram groups predictors by their correlation. Scatterplots of pairs of predictors with Spearman correlation coefficients greater than 0.8 will be created.

#### Redundancy (VE3)

Variance inflation factors will be computed between the candidate predictors. This will be done for the three possible candidate models, and using all complete cases in the respective candidate predictor sets. Redundancy will further be explored by computing parametric additive models for each predictor in the first two candidate models using the Hmisc::redun function.

### Bacteremia study: results of IDA

The full results of IDA according to the IDA plan are available in the Supplementary File. Moreover, our accompanying website https://stratosida.github.io/regression-regrets/ also provides the R code for full reproducibility. The main findings of IDA can be understood from the selected results described below.

#### IDA domain: missing values (M)

There is no instance of unit missingness in the sample dataset. Outcome variable and structural variables are completely observed. An analysis with only the key predictors hardly suffers from missing values (94% complete cases). If the predictor set is extended to include those of medium importance, the proportion of cases included by a complete case analysis decreases to only 63.9%. Extending to all predictors, only 27% of the observations would be complete. Individual predictors are missing with proportions of 48% and less. Only seven predictors have missingness proportions of more than 20%, and ten predictors between 10 and 20%. The remaining 32 predictors have smaller missingness proportions. Age and sex, the structural variables, are never missing. Completeness of predictors does not vary between groups defined by the structural variables.

**Fig. 2:**
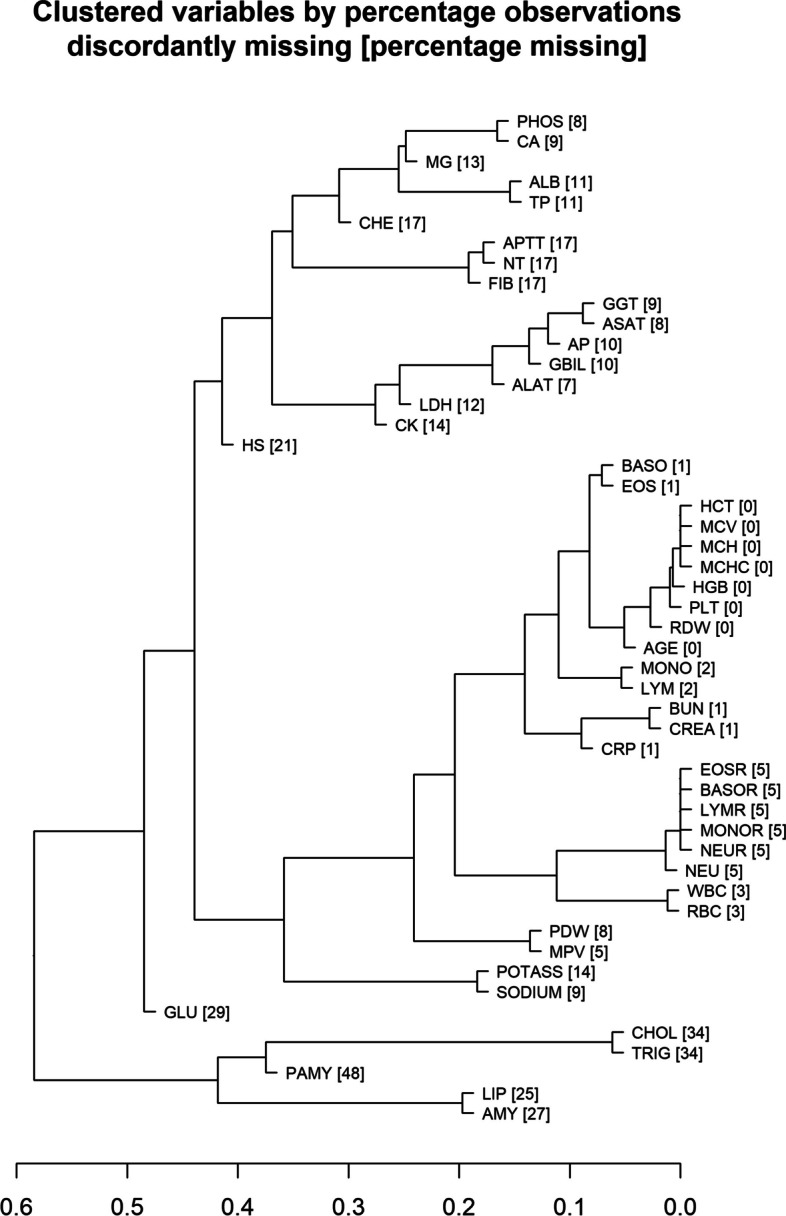
Missingness pattern among predictors depicted as dendogram. Numbers in brackets are percentages of missing values per variable. The position of each split on the horizontal axis corresponds to the proportion of discordant missingness between its branches. For example, the missingness status (present or missing value) for Lipases and Amylase (at the bottom of the graph) disagrees in about 20% of the observations

We also investigated the concordance of missingness between predictors (Fig. 2). GLU, PAMY and HS show very individual missingness patterns with more then 20% discordance with the patterns of any other predictors. Some groups of predictors have much lower discordance than missingness proportions, which points towards very similar missingness patterns. For example, AMY and LIP; TRIG and CHOL; or FIB, NT and APTT are such groups.

#### IDA domain: univariate distributions (U)

Many of the predictors measure concentrations of chemical compounds in the blood or represent cell counts. These predictors typically exhibit skewed distributions.

**Fig. 3:**
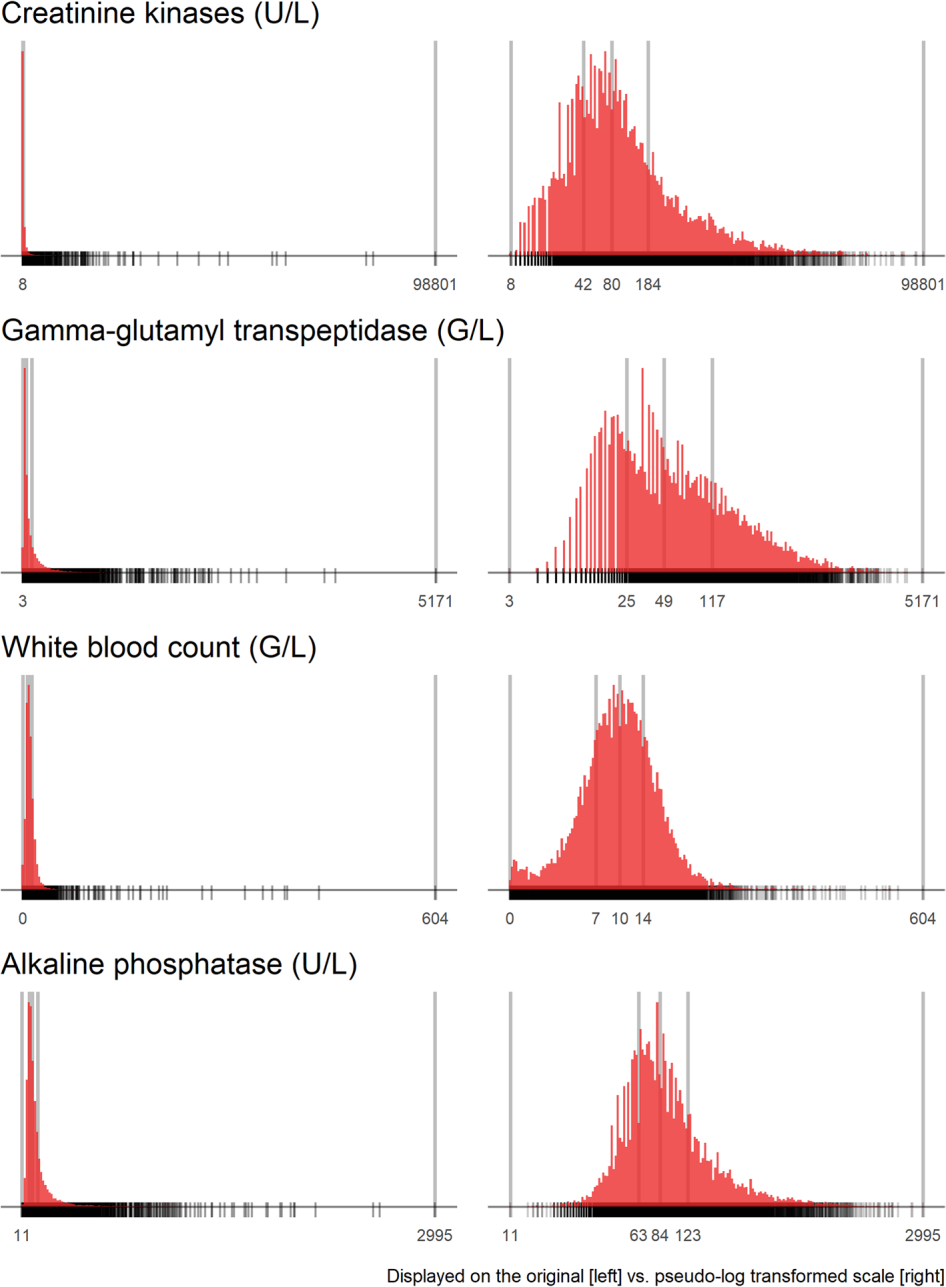
Four predictors for which a pseudo-log transformation enhanced graphical displays. The vertical bars refer to the quartiles and extremes of the distribution. Rugs on the bottom of the diagrams indicate location of data

For 15 predictors a pseudolog transformation increased the correlation with normal deviates by more than 0.2 correlation units compared to not transforming the predictor. For these predictors, original and transformed distributions have been compared (cf Fig. 3 for four examples), and in scatterplots (IDA domain: multivariate analyses) the transformed values will be used. We also evaluated cube root transformations but that transformation reduced skewness only very moderately.

We also investigated if there were spikes at specific values, by listing the five most frequent values of each predictor. For example, for basophiles and eosinophiles the most frequent value was 0 and occurred with a frequency (proportion) of 12,671 (87%) and 6,994 (48%), and corresponding concentration ratios (ratios of highest frequency and average frequency) were 15.7 and 17.3, respectively.

#### IDA domain: multivariate descriptions (V)

Absolute Spearman correlation coefficients of predictors with AGE, stratified by SEX, were generally below 0.3. Only few predictors had Spearman correlation coefficients with AGE between 0.2 and 0.3.

Some Spearman correlations coefficients between pairs of predictors were greater than 0.8, e.g. between WBC and NEU; between EOS and EOSR; BASO and BASOR; RBC, HGB and HCT; MPV and PDW; and LYMR and NEUR. These pairs were investigated by scatterplots. The high correlations could be explained by domain expertise.

For 23 out of 1,225 pairs of predictors Spearman and Pearson correlation coefficients differed by more than 0.1. Scatterplots of these pairs should be reviewed for anomalies.

Among the key predictors and the predictors of medium importance, WBC and NEU exhibited the highest degree of redundancy with variance inflation factors above 7 (key predictor set) or even above 14 (key and medium importance predictors). Including all remaining predictors, many predictors became almost exactly redundant. Variance inflation factors increased when considering parametric additive models instead of linear models.

## Possible consequences of IDA

In this section, we will describe how specific IDA results may be used in the regression analysis to follow. The possible impact of IDA has three aspects: it may induce refinements of the intended statistical analysis and help defining the statistical analysis plan, help avoiding misinterpretation of results of the regression analysis, and its results will also support decisions on how to present results of the regression analysis.

### Bacteremia study: refinements of the analysis strategy triggered by IDA results

Revisions of the analysis strategy based on the results of IDA are justified if any predictor-outcome associations were strictly not evaluated during IDA. An update of the analysis strategy could encompass a refinement of the model specifications, additional analyses, such as sensitivity analyses, or possibly a change to the intended analysis methods. In our examples, we show that large proportions and specific patterns of missing values, skewed distributions of predictors, and a high degree of redundancy between predictors may suggest that the plan should be updated. Furthermore, IDA findings might lead to planning of sensitivity analyses before formal statistical analyses commence.

In this example, the number of possible candidate predictors is relatively large if the final model should be ‘parsimonious’ and explainable. Here IDA can provide the necessary information to guide model building. This is often an iterative process that depends on observed features of the data, as the maximum correlation and number of complete cases change if predictors are removed. We consider IDA as the first iteration of this process. Further steps will then be carried out by the modeling team.

Predictors with univariate distributions that are particularly narrow, or, in case of categorical variables, that are extremely unbalanced may contribute only very little to predictive performance because most of the subjects are similar. The chances that such a variable is a strong predictor of the outcome is very low.

Given their histograms, basophiles (BASO) and the associated basophiles ratio (BASOR), and probably eosinophiles (EOS) and the eosinophiles ratio (EOSR) could be candidates for discarding because of their excessive spike at zero, which cannot be removed by any transformation. Given that the aim of the analysis is to predict bacteremia accurately, these variables are probably unlikely to contribute to overall predictive accuracy.

Predictors are also expected to add only little or no predictive performance if they are redundant to other predictors in the model. However, sometimes a reparametrization of the predictor space may remove the redundancy and enhance interpretability of the model. Analysis of the predictors’ multivariate distributions revealed that two predictors *a priorily* rated as important for predicting bacteremia exhibited a very high correlation: leukocytes (WBC) and neutrophiles (NEU). This correlation stems from the fact that neutrophiles are the biggest subtype of leukocytes. As a consequence of the correlation and the background information, one could replace WBC with a new variable WBC_NONEU = WBC – NEU. Using NEU and WBC_NONEU retains all the information of the two predictors for the model and keeps their regression coefficients interpretable but removes the high correlation (see also [21]).

From our IDA analysis, we would probably conclude that among the leukocyte-related predictors, only the computed WBC_NONEU (leukocytes minus neutrophiles) and NEU should be retained in an analysis with an extended candidate set, probably also MONO and LYM. The corresponding ‘ratio’ variables (MONOR, LYMR, NEUR) may not be needed for modeling as they are largely collinear with their absolute counterparts.

Likewise, one may also consider to remove those predictors that exhibit large proportions of missing values. Assume that a predictor with many missing values is closely associated with other predictors. In this case, it may be well imputable but is not likely to add predictive value on top of those other predictors. If that predictor in question is not associated with other predictors, it may not be well imputable and hence its imputation will introduce noise into the analysis. Hence, removing such variables may be indicated. It is not so clear, unfortunately, when to disregard a predictor because of its proportion of missing values. The threshold value may depend on how many predictors are affected by missing values, how much they are affected, and how the missingness pattern looks like. In our example, probably PAMY, TRIG and CHOL, all exhibiting more than one third of their values missing, may be the most obvious candidates for omission. Moreover, the following predictors all have missingness proportions of more than 20%: GLU, AMY, LIP, and HS. According to the missingness pattern dendogram, PAMY, TRIG, CHOL, AMY and LIP also have highly correlated missingness, suggesting that they do not serve each other in imputation models (Fig. 1).

Hence, 14 predictors (BASO, EOS, BASOR, EOSR, MONOR, LYMR, NEUR, PAMY, TRIG, CHOL, GLU, AMY, LIP, HS) could be excluded from model building without having to expect reduced predictive accuracy, which reduces the dimensionality of the predictor space, without unblinding the association of the predictors with the outcome, from 51 to 37. The modeling team will recompute the numbers of observations with complete recordings for all remaining predictors.

Generally, many different modeling strategies to handle nonlinear associations of predictors with the outcome are available, e.g. restricted cubic splines, penalized splines or fractional polynomials [3]. For example, restricted cubic splines provide a linear fit outside of the boundary knots, and one may want to adjust default knot positions in case of very skewed distributions. High-resolution histograms may help in such decisions. In our IDA, we already showed histograms based on pseudolog transformations of some predictors. These transfomations were necessary in scatterplots to enhance their interpretability, but whether to use transformed predictors in the outcome models may be debatable. Royston and Sauerbrei [22] discussed other ways of pretransforming predictors to increase robustness of models, in particular at the tails of the predictor support.

If interactions of predictors have been pre-specified, IDA may evaluate the joint distribution of these predictors. Strong association of the predictors involved in an interaction may make the inclusion of their interaction unnecessary as it would come with great estimation uncertainty (cf. [23], p. 301). For example, we included scatterplots of all predictors with age, stratified by sex in our IDA. Among the key predictors, BUN had the highest correlation with age with correlation coefficients of 0.487 (males) and 0.386 (females), while there was hardly any correlation of bacteremia and PLT. Hence, interaction terms involving age and PLT can be more precisely estimated than interaction terms involving age and BUN.

Sensitivity analyses, which are in general not part of IDA, are a tool to evaluate the robustness of estimates on decisions in model building, for example choices of different methods, impact of variable selections, or impact of strategies to handle missingness or influential points. Sensitivity analyses should be pre-specified, and IDA may suggest that certain sensitivity analyses are necessary to back up the modeling results. Regarding missing values in the bacteremia study, one could perform such a sensitivity analysis by not imputing any predictors of minor importance but just omitting them from the model. One may also consider to transform some predictors with particularly skewed distributions before modeling. While this may lead to differences in the interpretation of the associated regression coefficient (if a linear functional form is chosen for such a predictor), one could evaluate if it also affects prediction performance or the values of the standard errors of the other covariates’ regression coefficients (see Additional file 2 Appendix B.2 for an example). About dealing with the strong correlation between WBC and NEU, one could define such a sensitivity analysis by removing either WBC or NEU from the model and evaluate if this has a relevant effect on the model’s performance.

In all three cases, such sensitivity analyses are consequences of IDA but they are still predefined in the sense that they are planned before uncovering the association of the outcome with the predictors [14, 24, 25].

By contrast, for example, sensitivity analyses that result from observing an unexpected pattern in the residuals of a model (e.g. if residuals show a clear nonlinear association with a predictor) must be seen as post-hoc analyses. Modifying the model because of such an unplanned sensitivity analysis increases the risk of overfitting the model. Nevertheless, it should be done and reported as a post-hoc analysis.

### Bacteremia study: how IDA may guide the interpretation of modeling results

The results of the regression model consists of the estimated regression coefficients, their covariance matrix and in particular their standard errors, may include predictions for selected predictor patterns and will also comprise measures of model performance.

#### Skewed distributions

Skewed distributions of predictors may have consequences on the precision and the robustness of these results, and knowledge about the distributional shapes of the predictors are essential for interpretation. As revealed by our IDA, some of the predictors exhibited highly skewed distributions. For these predictors, the estimation of the nonlinear functional forms may suffer from disproportional impact of some observations, and estimation uncertainty will be reflected by wide confidence intervals. Impact of highly influential points may be reduced by pretransforming the predictors to more symmetric distributions, which however may change their interpretation if finally a linear functional form is chosen. Alternatively, the values could be winsorized before modeling as previously suggested [22, 24]. In addition, extreme values should be assessed for implausibility and, if classified as such, potentially removed. In general, there are numerous ways to make analyses robust against such influential points, including transformation, robust regression or by estimating robust variances [22, 26, 27].

#### Transformation of predictors

If a predictor has been transformed, regression coefficients are given for units of the transformed predictor. In case of the pseudo-log transformation using a base of 10 that was suggested for BUN, they would correspond to the difference in outcome expected for a tenfold increase in the original predictor. This correspondence is only approximate as a pseudo-log transformation was used. See Additional file 2 Appendix B.2 for analyses of the bacteremia study with and without preceding pseudo-log transformation of predictors. If for WBC and NEU, pseudo-log transformations will be used in modeling the data, a unit of pseudo-logarithm would correspond roughly to a tenfold of the original WBC or NEU. The range of the pseudo-logged values is about 1.5; thus a unit difference covers almost the entire range of the data and comparably large regression coefficients have to be expected. See Additional file 2 Appendix B.3 for an illustration.

#### Validity of predictions

IDA allows to identify the support of a model, i.e., the ranges of values of the predictors from which the model was derived and to which it should be applicable. Predictions for observations from areas with higher joint density of predictors are more precise, while predictions with smaller support are less precise. The joint distribution also helps to understand in which cases predictions would actually be extrapolations. For example, in Fig. 3 the density of data points in any of the age-sex-groups is very low beyond a value of the pseudolog of WBC (t_WBC) greater than 1.5. The support is also essential to understand measures of model performance. Usually, the wider the support of a model, the more variance in the outcome can potentially be explained, and hence measures like the area under the ROC curve or the R-squared also tend to be greater. See Additional file 2 Appendix B.4 for an illustration.

#### Missing data handling

While a method to handle missing data is usually prespecified, IDA can give some information to support this decision or put it into question. If multiple imputation was prespecified, it has to be expected that regression coefficients of predictors with higher proportion of missing values will generally be estimated more imprecisely compared to those with fewer missing values, relative to comparing these quantities after complete case analysis. Consequently, such predictors will seem less important in the final model than more complete predictors, given they have approximately equal association with the outcome. Hence the decision whether to apply multiple imputation *vs*. using complete case analysis may impact assessments of the contribution of individual predictors to model instability. See also Additional file 2 Appendix B.5 for an illustration.

#### Interpretation of nonlinear functional forms

For predictors for which a nonlinear functional relationship with the outcome is assumed, the partial response function (predicted values vs. predictor) will usually be evaluated graphically. Areas in which this response function has a wide confidence interval correspond to low support in observed predictor values, and such a low support may preclude the precise estimation of a nonlinear functional form. In Supplemental Appendix B.1 we used a simplified fractional polynomial model for bacteremia status to illustrate the interplay between decisions to apply transformation to predictors before model building and their consequences on the estimated functional forms. In Additional file 2 Appendix B.6 we show an example where a nonlinear effect of a predictor was identified, but in the most relevant subrange of the predictor where the data is dense, the estimated nonlinear functional form agreed well with a straight line.

#### Predictor selection or reparameterization of predictors

If two correlated predictors are considered for a model (like WBC and NEU), interpretation may be difficult if the correlation results from the definition of the predictors. In the example with WBC and NEU, WBC cannot stay constant while varying NEU because neutrophiles are a component of leukocytes. Above we suggested to replace WBC by WBC_noNEU and then WBC_noNEU and NEU can vary independently, ensuring interpretability of regression coefficients.

### How IDA may guide the presentation of results

While in this paper we intentionally do not present the actual modeling results for the bacteremia study, we give some general remarks on how IDA may guide the presentation of such results.

Transformations of predictors included in prediction models should be appropriately documented and reported. For continuous predictors, IDA suggests appropriate unit increments of predictors to which regression coefficients or derived quantities such as odds or hazard ratios should correspond (e.g. 1 year, 5 year or 10 year increments of age). Numerous examples from the medical literature demonstrate that this is often ignored, and one can find reports of regression coefficients of a continuous predictor and confidence limits that are all close to parity. For example, Ma et al. [28] report an adjusted risk ratio of CRP (95% confidence interval) of 0.982 (0.973, 0.991) with a *p*-value < 0.001 for predicting survival of persons admitted to a hospital with COVID-19. When considering the reported interquartile ranges (7.52 to 37.93 mg/L for survivors, and 35.52 to 148.31 mg/L for non-survivors), it becomes apparent that a unit difference in CRP in this study cohort is probably not an appropriate choice for presenting the model, if interpretability is a goal.

Royston and Sauerbrei [24, p. 54f] discuss choosing an appropriate reference category for categorical predictors in a regression model. While there may be background knowledge to support the choice of a specific category as the reference, IDA may be used to ensure that the ‘sample size (of the referent category) should not be too small’ to avoid inflation of standard errors for all comparisons to the reference [24, p.55].

In our example, one could be interested in presenting partial dependence of predictions on predictors by displaying the estimated response function of predictors. IDA guides the choice of an appropriate range for the x-axis, which will be either the range of the predictor or a bit less than the range depending on the data sparsity in the tails of its distribution. One could also use a scaling of the x-axis that corresponds to the transformation that was deemed appropriate to symmetrize the distribution of the predictor. See Additional file 2 Appendix B.2 for illustrations.

All changes to the prespecified analysis and reporting strategy induced by IDA must be transparently reported in a statistical methods summary for the statistical report. In this example we did not suggest specific changes, but only illustrated which aspects of an analysis plan could be further refined or put into question once the IDA results are available. For each of these refinements, usually many options are possible and specific choices may depend on the preferences and experience of the analysis team.

In this application with 50 possible candidate predictors to choose from there is a lot of emphasis on how to use IDA to guide model building by disregarding predictors in the analysis. This is of course very specific to this example, and IDA is not always related to this aspect.

## Discussion

In this paper we proposed a set of elements of initial data analysis that may help a data analyst in designing an IDA plan in conjunction with the statistical analysis plan. While we focused on studies in which a descriptive or predictive research question is addressed with a statistical regression model, it can be adapted to other studies. In this context, IDA has the purpose to inform the data analyst and the domain expert about key properties of the data, without exploring the predictor-outcome association. The IDA findings are essential to empirically support the choice of the original analysis strategy or to guide revisions. They are also key to correct interpretation of the analysis results. An IDA plan should balance an exhaustive investigation of the dataset with utility. It should have sufficient details to detect features of a data set that could affect a regression analysis, or the interpretation or presentation of results. It should cover necessary steps informed by the reseach aims and pre-specified analysis strategy in a systematic approach, to avoid missing items or overlooking important findings in lengthy template reports. While the IDA framework comprises six elements, here we devised a strategy to develop elements of an IDA plan from data screening onwards, balancing utility and parsimony. The strategy could be seen as a recommended minimum set of analyses that an IDA plan should contain in order to prepare for regression analysis. The IDA checklist can be used as a starting point for an analyst to design an IDA plan tailored to a specific study and possibly adding other aspects. Simplifications may be appropriate, in particular in the IDA domain of multivariate descriptions, for example if only few predictors are considered.

We included the outcome variable in univariate evaluations, but intentionally excluded it from any bivariate or multivariate analysis, as IDA shall not anticipate the main analysis. This principle distinguishes IDA from exploratory data analysis (EDA), where associations in the data are explored and new hypotheses can be generated. To protect against arriving at wrong conclusions from prematurely evaluating the association of the outcome with the predictors when they are performing IDA, one could generate an outcome-blinded ‘IDA data set’ from the main analysis set. In such a ‘blinded’ IDA data set, the outcome variable is detached from the predictors and permuted relative to the predictors, such that any associations of the outcome with predictors variables are destroyed and any apparent associations meaningless, but associations between the predictors retained. The conclusions from our IDA example analyses are unchanged had they been conducted on such a blinded IDA data set. This approach imitates a blinded data review in clinical trials [29].

In the IDA plan statements on how potential IDA findings may guide the decisions in adaptive analysis strategies, e.g. how to handle missing data or selecting predictors for a model, should be deterministically prespecified [30]. Well thought-out, systematic adaptive analysis strategies are reproducible unlike ad-hoc decisions during the analysis process [31]. Hence, an IDA plan enhances the statistical analysis strategy, and relevant IDA methods should be incorporated in the methods section of a research report. Even informal or exploratory analysis projects that are not intended to be reported in the scientific literature, will benefit from a systematic approach to IDA and separating IDA activities from the main analyses. Some IDA findings may suggest changes in the intended analyses that were not foreseeable, such as transformation of predictors, a refinement of the statistical model, or additional sensitivity analyses. The IDA findings that lead to such changes or IDA findings that help interpret the model results should be explicitly reported as results or in the discussion [25]. Transparent reporting of the planned and actually conducted analyses, as well as the reasons for the changes in an analysis plan, are essential for ensuring reproducibility and repeatability. Adequate reporting of research has been under discussion [15, 32] and we suggested reporting strategies of IDA for research papers [5]. Adapting structured reporting for IDA, as first proposed in the REMARK profile [25] and later extended [33] may also help to provide a better overview of all IDA steps. IDA augments knowledge about a dataset and transparency in reporting will aid in accessible and reusable data according to the FAIR principles [34]. Of note, a range of R packages may facilitate the conduct of several aspects of IDA as well as data quality [35, 36].

In this paper we focused on a predictive research question, but our recommendations may also guide the planning of IDA for descriptive or explanatory research questions, including the estimation of an adjusted exposure-outcome association, and of models that estimate causal effects. A separate article discusses IDA for longitudinal studies [19]. Our guidance may also help in designing a systematic approach to data screening for clinical trials, in particular if covariate adjustment is used, and may then be applied before treatment allocation is unblinded. We also expect that our recommendations may be useful for researchers fitting models with modern algorithmic approaches.

In summary, we provide practical recommendations for an IDA plan and how to carefully examine data properties to improve analyses and reproducibility of results. Our hope is that this empowers researchers to follow a systematic strategy for IDA.

### Supplementary Information

Supplementary Material 1.

Supplementary Material 2.

## Data Availability

All R code can be found at https://stratosida.github.io/regression-regrets. The data of the bacteremia study can be found at https://zenodo.org/records/7554815.
