# Supplementary material for: Regression without regrets –initial data analysis is a prerequisite for multivariable regression
Source: BMC Med Res Methodol. 2024 Aug 8;24:178. doi: 10.1186/s12874-024-02294-3 (PMC11308558; doi:10.1186/s12874-024-02294-3)
Supplement: Supplementary file 1 — Supplementary Material 1. [file 12874_2024_2294_MOESM1_ESM.docx]

# Additional file 1. Prerequisites and optional extensions for a general strategy to develop an IDA plan

Georg Heinze^*,1^, Mark Baillie^2^, Lara Lusa^3,4^, Willi Sauerbrei^5^, Carsten Oliver Schmidt^6^, Frank E. Harrell Jr^7^, Marianne Huebner^8^

on behalf of the Topic Groups “Initial Data Analysis” and “Selection of Variables and Functional Forms in Multivariable Analyses” of the STRATOS Initiative (STRengthening Analytical Thinking for Observational Studies, <http://www.stratos-initiative.org>)

2024-03-27

Contents

[Section S1. Prerequisites 2](#_Toc162533491)

[Section S2. Further aspects and possible extensions of IDA plans 4](#_Toc162533492)

[IDA domain extension: missing values 4](#_Toc162533493)

[IDA domain extension: univariate descriptions 4](#_Toc162533494)

[IDA domain extension: multivariate descriptions 5](#_Toc162533495)

[References 7](#_Toc162533496)

## Section S1. Prerequisites

Initial data analysis is aligned with research aims and background information about the study. This is described below.

(i) Research aim: The research aim should be clearly defined as either descriptive (estimating one or several associations of an outcome variable with predictors), predictive (validating, updating or newly developing a prediction model), or causal (estimating the causal effect of an intervention on the outcome) (Table 2, PRE1). If the research aim is descriptive, it should be clarified which predictor-outcome associations in the target population are of central interest and should be estimated and described. For a newly developed prediction models, is the transparency of the model important? For causal questions, the intervention in question should be clearly defined. It should also be clear whether the research objective is to be regarded as exploratory or confirmatory.

(ii) Analysis strategy: We assume that the analysis strategy has been specified in a statistical analysis plan (Table 2, PRE2). To plan IDA, knowledge about the set of predictors to be considered in a model, the outcome variable, and the analytical strategy to build the regression model are necessary. Any assumptions of the model should be stated.

(iii) Data dictionary and metadata: a detailed data dictionary should be available, which informs, amongst others, about the meaning of each variable, the units of measurement, the possible levels in case of categorical variables, admissible values or value ranges (Table 2, PRE3). More generally, metadata also refer to information about the study design and data collection processes.

(iv) Domain expertise, predictor grouping, variables of interest, structuring (Table 2, PRE4):

- Groups of predictor variables may be considered, for example according to their biological context as in the dataset described below, which may influence the analysis strategy and may also help to structure IDA.
- Missing value reasons: if not already specified in metadata, domain experts should be consulted to explain possible reasons for the occurrence of missing values for each predictor.
- Domain expertise can provide valuable insights into the anticipated shape of predictor distributions and potential correlations. Such knowledge can be informally summarized or synthesized into graphs linking predictors based on these suggestions [1]. The information provided later aids in evaluating observed distributions and associations.
- Structural variables: in the context of IDA, we define structural variables as those that help to structure IDA results for a clear organization and essential overview of data properties. Structuring can be based on levels of measurement (centers), on calendar time of recruitment, on demographic variables such as sex or age, or on variables of central importance to the research questions. Often the association of predictors with structural variables such as centers or time is of interest, and multivariate distributions of predictors may be easier to understand if stratified by structural variables such as sex. Ideally, structural variables are completely observable for all individuals. If a research aim includes many structural variables, it may be necessary to prioritize some of them for the task of structuring IDA. There may also be studies where no such structural variables can be identified. Structural variables may or may not be included as predictors in the analysis strategy. IDA analyses may also be structured by first describing predictors that are deemed more important to predict the outcome, followed by less important predictors.

## Section S2. Further aspects and possible extensions of IDA plans

### IDA domain extension: missing values

A framework for the treatment and analysis of missing values in observational studies (TARMOS framework) has been developed and described by STRATOS-TG1 [2]. Here we note two aspects of that framework that could be considered as parts of IDA:

- “A table of characteristics for the ‘complete’ versus ‘incomplete’ (or all) participants, or by whether variables with substantial missingness are observed.” [2] (Table 2, ME1)
- “An assessment of the predictors of missingness, e.g. using a logistic regression model fitted to an indicator for being a complete record, and predictors of missing values i.e. associations with the incomplete variables.” to make inferences about potential mechanisms underlying missing data [2]. (Table 2, ME1)

### IDA domain extension: univariate descriptions

- Distributional plots may be useful to allow identification of areas with no or sparse data, or to identify extreme values that could have disproportional influence on regression results (Table 2, UE1).
- If an unexpected distribution is identified in the ‘IDA domain: univariate distributions’, special attention should be given to evaluate bivariate distributions of predictors with this variable. For example, a skewed or multimodal univariate distribution for a predictor can result from a strong correlation of that predictor with other predictors. Multimodality in a distribution might also require further investigations, for e.g. possible measurement errors or digit preference. Of note, if a predictor exhibits a skewed distribution it may be difficult to depict in bivariate scatterplots, and the axis may need transformation.

### IDA domain extension: multivariate descriptions

Once decisions on how to handle missing values and how to include predictors in a model have been made, more data screening may follow which is then performed by the modeling team. Specifically, these aspects may complement the basic set of multivariate analyses when needed:

- Results from different correlation metrics could be compared (Table 2, VE1). Large differences of these metrics (e.g., measuring linear and monotone association) for a pair of predictors may indicate unusual types of association or outliers in a bivariate data cloud. In particular if there are many predictor variables, one could focus on the scatterplots of such pairs of predictors to investigate the data pattern.
- Variable clustering: identifies clusters of predictors that are closely associated (also contained as dendrogram in the heat map) (Table 2, VE2). Such clusters may give rise to model simplifications.
- Redundancy analysis: identifies if a predictor is (almost) entirely represented by a linear combination or generalized additive model of other predictors [3] (Table 2, VE3).

In some regression problems, specific further analyses may become relevant:

- With categorical predictors, correspondence analysis may be helpful to explore their associations graphically [4]
- In case of a mix of continuous and categorical predictors (which is the rule rather than the exception), computing variance inflation factors for each design variable allows to identify redundancies at the modelling level. Design variables are all variables that code predictors for the model. Categorical predictors are usually coded as several binary dummy variables. Purposeful dummy coding (e.g. concerning the choice of reference category, or using ordinal coding for ordinal predictors as proposed by [5]) later facilitates a meaningful interpretation of the regression coefficients. Continuous predictors with an assumed nonlinear association with the outcome are coded with basis variables (e.g. spline bases or fractional polynomials). As this aspect does not involve the outcome variable, it may be seen as IDA, but usually it will be part of the planned analyses.
- Depending on the scale of measurement, association between predictors could be visualized by scatterplots (continuous by continuous), or dotplots of original values (continuous by categorical), or frequencies (categorical by categorical). Ideally, these graphical displays should show the association of each predictor with all structural covariates simultaneously (Table 2, V2). For example, to display associations of a predictor X with the assumed structural covariates age and sex one could show scatterplots of X by age for males and females in two panels, or superimpose the two scatterplots using different symbols for males and females. If among the structural covariates there are two continuous ones, one of them could be categorized, but that bears the risk of overlooking patterns in the association of the predictor with that structural covariate. With many predictors evaluated, one should be aware that this approach could catch chance findings which may be overinterpreted.
- In situations with ‘many’ predictors, a heat map visualising the clustering of observations on one axis and of variables on the other axis may be a useful summary of the correlation structure of the independent variables. The structural covariates could be added and highlighted in the heat map.
- Multivariate analyses are particularly vulnerable to missing values, as only observational units which are complete in the considered predictor variables can be included. While imputation methods can be used to reconstruct missing data, we consider these model-based methods as part of modeling and not of IDA.

## References

[1] Heinze G, Wallisch C, Dunkler D. Variable selection – A review and recommendations for the practicing statistician. Biometrical Journal 2018;60(3):431-449. https://doi.org/10.1002/bimj.201700067

[2] Lee KJ, Tilling KM, Cornish RP, Little RJA, Bell ML, Goetghebeur E, Hogan JW, Carpenter JR; STRATOS initiative. Framework for the treatment and reporting of missing data in observational studies: The Treatment And Reporting of Missing data in Observational Studies framework. J Clin Epidemiol. 2021;134:79-88. doi: 10.1016/j.jclinepi.2021.01.008.

[3] Harrell Jr FE, Dupont C. Hmisc: Harrell MIscellaneous. R package version 4.7-0. https://cran.r-project.org/package=Hmisc

[4] Sourial N, Wolfson C, Zhu B, Quail J, Fletcher J, Karunananthan S, Bandeen-Roche K, Béland F, Bergman H. Correspondence analysis is a useful tool to uncover the relationships among categorical variables. J Clin Epidemiol. 2010;63(6):638-46. https://doi.org/10.1016/j.jclinepi.2009.08.008.

[5] Royston P, Sauerbrei W. Multivariable model-building. a pragmatic approach to regression analysis based on fractional polynomials for continuous variables. Wiley. Chichester; 2008.
