# Supplementary material for: Regression without regrets –initial data analysis is a prerequisite for multivariable regression
Source: BMC Med Res Methodol. 2024 Aug 8;24:178. doi: 10.1186/s12874-024-02294-3 (PMC11308558; doi:10.1186/s12874-024-02294-3)
Supplement: Supplementary file 2 — Supplementary Material 2. [file 12874_2024_2294_MOESM2_ESM.docx]

Additional File 2:
Regression without regrets - worked example

Mark Baillie

Georg Heinze

Marianne Huebner

2024-07-01

Table of contents

[Preface 2](#_Toc164148573)

[1. Bacteremia study 2](#_Toc164148574)

[1.1 Bacteremia study overview 2](#_Toc164148575)

[1.2 Where to access the data? 3](#_Toc164148576)

[2. IDA plan 3](#_Toc164148577)

[2.1 Prerequisites for the IDA plan 3](#_Toc164148578)

[2.2 IDA data derivations 4](#_Toc164148579)

[2.3 IDA planned analyses 5](#_Toc164148580)

[3. Analysis ready data for IDA 7](#_Toc164148581)

[3.1 Analysis ready dataset 7](#_Toc164148582)

[4. Results of IDA: Missing values 9](#_Toc164148583)

[4.1 M1: Participant missingness 10](#_Toc164148584)

[4.2 M2: Variable missingness 10](#_Toc164148585)

[4.3 M3: Complete cases 10](#_Toc164148586)

[4.4 M4: Patterns of missing values 11](#_Toc164148587)

[5. Univariate distribution checks 13](#_Toc164148588)

[5.1 U1: Categorical variables 13](#_Toc164148589)

[5.2 Continuous variables 14](#_Toc164148590)

[6. Multivariate analyses 20](#_Toc164148591)

[6.1 V1: Association with structural variables 21](#_Toc164148592)

[6.2 V2: Correlation coefficients between all predictors 24](#_Toc164148593)

[7. Final Analysis ready data 34](#_Toc164148594)

[7.1 Final derivations and transformations 34](#_Toc164148595)

[7.2 Save final analysis ready data sets 35](#_Toc164148596)

[References 35](#_Toc164148597)

[Appendix A — Pseudo-log transformations 36](#_Toc164148598)

[A.1 Introduction 36](#_Toc164148599)

[A.2 Impact of choice of $\sigma$ 36](#_Toc164148600)

[A.3 Finding a parameter that best achieves normality 42](#_Toc164148601)

[Appendix B — Supplementary Example 54](#_Toc164148602)

[B.1 Overview 54](#_Toc164148603)

[B.2 Example 1: to transform or not to transform 58](#_Toc164148604)

[B.3 Example 2: Interpretation of regression coefficient ‘size’ 61](#_Toc164148605)

[B.4 Example 3: the support of a model determines what it can explain 64](#_Toc164148606)

[B.5 Example 4: the limits of mulitiple imputation 66](#_Toc164148607)

[B.6 Example 5: Plot of functional form should focus on areas with high density 67](#_Toc164148608)

[Appendix C — Source data 70](#_Toc164148609)

[C.1 Data dictionary 70](#_Toc164148610)

[Appendix D — Results of IDA: Missing values Appendix 73](#_Toc164148611)

[D.1 M2: Variable missingness for remaining predictors 73](#_Toc164148612)

[Appendix E — Univariate distribution checks 74](#_Toc164148613)

[E.1 U2: Descriptive summaries 74](#_Toc164148614)

[Appendix F — Multivariate analyses 83](#_Toc164148615)

[F.1 V1: Association with structural variables 83](#_Toc164148616)

[F.2 VE3: Redundancy 84](#_Toc164148617)

# Preface

The focus of this report is to provide guidance on conducting initial data analysis in a reproducible manner in the context of intended regression analyses. The code to reproduce all analyses shown here is given at our accompanying website https://stratosida.github.io/regression-regrets/.

# 1. Bacteremia study

## 1.1 Bacteremia study overview

We will exemplify our proposed systematic approach to data screening by means of a diagnostic study with the primary aim of using age, sex and 49 laboratory variables to fit a diagnostic prediction model for the bacteremia status (= presence of bacteria in the blood stream) of a blood sample. A secondary aim of the study is to describe the functional form of each predictor in the model. Between January 2006 and December 2010, patients with the clinical suspicion to suffer from bacteremia were included if blood culture analysis was requested by the responsible physician and blood was sampled for assessment of hematology and biochemistry. An analysis of this study can be found in Ratzinger et al. (2014).

The data consists of 14,691 observations from different patients and 51 potential predictors. To protect data privacy our version of this data was slightly modified compared to the original, and this modified version was cleared by the Medical University of Vienna for public use (**DC 2019-0054**). Compared to the official results given in (Ratzinger et al. 2014), our results may differ to a negligible degree.

## 1.2 Where to access the data?

We refer to the **source** data as the raw data set available in this repository (**DC 2019-0054**). The data set is published on [Zenodo](https://doi.org/10.5281/zenodo.7554815) with the following doi: https://doi.org/10.5281/zenodo.7554815.

For simplicity, we have also stored the *source* data and accompanying materials such as the **data dictionary** in the data-raw folder. The data dictionary provides an overview of the collected *source* data - see [Appendix C.1](#sec-data_dict) for further details. Within the appendix, we also display a short snapshot of source data set from the data-raw folder of the project directory. The snapshot provides a glimpse of the data and the data dictionary for more context. However, we refer to the [Zenodo page](https://doi.org/10.5281/zenodo.7554815) for an interactive overview of the source data.

# 2. IDA plan

This document exemplifies the pre-specified plan for initial data analysis (IDA plan) for the bacteremia study.

## 2.1 Prerequisites for the IDA plan

### 2.1.1 PRE1: Research aims

We assume that the aims of the study are to fit a diagnostic prediction model for bacteremia based on 51 potential predictors and to describe the functional form of each predictor.

### 2.1.2 PRE2: Analysis strategy

The aims are addressed by fitting a logistic regression model with bacteremia status as the dependent variable. Based on domain expertise, the predictors are grouped by their assumed importance to predict bacteremia. Variables with known strong associations with bacteremia are age (AGE), leukocytes (WBC), blood urea neutrogen (BUN), creatinine (CREA), thrombocytes (PLT), and neutrophiles (NEU) and these predictors will be included in the model as key predictors. Predictors of medium importance are potassium (POTASS), and some acute-phase related parameters such as fibrinogen (FIB), C-reactive protein (CRP), aspartate transaminase (ASAT), alanine transaminase (ALAT), and gamma-glutamyl transpeptidase (GGT). All other predictors are of minor importance.

Continuous predictors should be modelled by allowing for flexible functional forms, where for all key predictors four degrees of freedom will be spent, and for predictors of medium and minor importance, three or two degrees of freedom should be foreseen at maximum, respectively. The decision on whether to use only key predictors, or to consider predictors also from the predictor sets of medium or minor importance depends on results of data screening, but will be made before uncovering the association of predictors with the outcome variable.

An adequate strategy to cope with missing values will also be chosen after screening the data. Candidate strategies are omission of predictors with abundant missing values, complete case analysis, single value imputation or multiple imputation with chained equations.

### 2.1.3 PRE3: Analysis Ready Data dictionary

The **source** data dictionary of the bacteremia data set consists of columns for variable names, variable labels, scale of measurement (continuous or categorical), units, plausibility limits, and remarks. See [Appendix C](#sec-source-data).

An additional [**analysis ready** data dictionary](https://docs.google.com/spreadsheets/d/1Ft5eyenvDnMBoLvJmcBaklfrYcwyW-rkt-ivIkaphdA/edit#gid=1128598743) will be created to capture additional derivation, transformations and metadata relevant to the research question.

### 2.1.4 PRE4: Domain expertise

The demographic variables age and sex are are chosen as the structural variables in this analysis for illustration purposes, since they are commonly considered important for describing a cohort in health studies. Key predictors and predictors of medium importance are as defined above. Laboratory analyses always bear the risk of machine failures, and hence missing values are a frequent challenge. This may differ between laboratory variables, but no a priori estimate about the expected proportion of missing values can be assumed. As most predictors measure concentrations of chemical compounds or cell counts, skewed distributions are expected. Some predictors describe related types of cells or chemical compounds, and hence some correlation between them is to be expected. For example, leukocytes consist of five different types of blood cells (BASO, EOS, NEU, LYM and MONO), and the sum of the concentration of these types approximately (but not exactly) gives the leukocyte count, which is recorded in the variable WBC. Moreover, these variables are given as absolute counts and as percentages of the sum of the five variables, which creates some correlation. Some laboratory variables differ by sex and age, but the special selection of patients for this study (suspicion of bacteremia) may distort or alter the expected correlations with sex and age.

## 2.2 IDA data derivations

Based on the prerequisites the following data derivations will be performed on the **source data** to form an **analysis ready data set** stored in the **data** folder.

The outcome variable from the source data **BC** will be renamed to **BACTEREMIA** to be more informative. A numeric variable **BACTEREMIAN** will also be derived to enable modelling with the following coding, 0: no, 1: yes.

For the purpose of stratifying IDA results by age, **AGE** will be categorized into the following three groups: [16, 50], (50, 65], (65, 101], both with numeric (**AGEGR01**) and character coding (**AGEGR01C**).

Predictors will be grouped by importance to the research question. Indicator flags for the predictor grouping will be derived:

- **KEY_PRED_FL01** will indicate the key predictors: AGE, WBC, BUN, CREA, PLT, and NEU
- **MED_PRED_FL01** will indicate the medium importance predictors: POTASS, FIB, CRP, ASAT, ALAT, GGT
- **REM_PRED_FL01** will indicator all remaining predictors not defined as key or medium importance.

Note that depending on the consequences of IDA, additional flags may be defined and derived to track the changes to the original analysis plan. For example, an addition to the key predictors can be handled by creating a new flag **KEY_PRED_FL02**, with an increment of the index to indicate that a change has occurred. This provides traceability to the consumer of the analysis data.

An additional metadata flag **PARCAT01** will be derived to indicate the blood cell variables which form the Leukocytes predictor (**WBC**): BASO, EOS, NEU, LYM and MONO.

## 2.3 IDA planned analyses

The following sections detail the IDA planned analyses.

### 2.3.1 M1: Participant missingness

As the data was obtained from the registry of the laboratory, and only performed laboratory analyses were included, participant missingness cannot be evaluated.

### 2.3.2 M2: Variable missingness

Numbers and proportions of missing values will be reported for each predictor separately. Type of missingness has not been recorded.

### 2.3.3 M3: Complete cases

The number of available complete cases (outcome and predictors) will be reported when considering:

1. the outcome variable (BACTEREMIA)
2. outcome and structural variables (BACTEREMIA, AGE, SEX)
3. outcome and key predictors only (BACTEREMIA, AGE, WBC, BUN, CREA, PLT, NEU)
4. outcome, key predictors and predictors of medium importance (BACTEREMIA, AGE, WBC, BUN, CREA, PLT, NEU, POTASS, FIB, CRP, ASAT, ALAT, GGT)
5. outcome and all predictors.

### 2.3.4 M4: Patterns of missing values

Patterns of missing values will be investigated by:

1. computing a table of complete cases (for the three predictor sets described above) for strata defined by the structural variables age and sex,
2. constructing a dendrogram of missing values to explore which predictors tend to be missing together.

### 2.3.5 U1: Univariate descriptions: categorical variables

For sex and bacteremia status, the frequency and proportion of each category will be described numerically.

### 2.3.6 U2: Univariate descriptions: continuous variables

For all continuous predictors, combo plots consisting of high-resolution histograms, boxplots and dotplots will be created. Because of the expected skew distribution, combo plots will also be created for log-transformed predictors.

As numerical summaries, minimum and maximum values, main quantiles (5th, 10th, 25th, 50th, 75th, 90th, 95th), and the first four moments (mean, standard deviation, skewness, curtosis) will be reported. The number of distinct values and the five most frequent values will be given, as well as the concentration ratio (ratio of frequency of most frequent value and mean frequency of each unique value).

Graphical and parametric multivariate analyses of the predictor space such as cluster analyses or the computation of variance inflation factors are heavily influenced by the distribution of the predictors. In order to make this set of analyses more robust to highly influential points or areas of the predictor support, some predictors may need transformation (e.g. logarithmic). We will compute the correlation of the untransformed and log-transformed predictors with normal deviates. Since some predictors may have values at or close to 0, we will consider the pseudolog transformation $f\left( x;\sigma\right)=sinh^{-1}\left( x/2\sigma\right)/log10$ (Johnson 1949) which provides a smooth transition from linear (close to 0) to logarithmic (further away from 0). The transformation has a parameter $\sigma$ which we will optimize separately for each predictor in order to achieve an optimal approximation to a normal distribution monitored via the correlation of normal deviates with the transformed predictor. For those predictors for which the pseudolog-transformation increases correlation with normal deviates by at least 0.2 units of the correlation coefficient, the pseudolog-transformed predictor will be used in multivariate IDA instead of the respective original predictor. For those predictors, histograms and boxplots will be provided on both the original and the transformed scale.

### 2.3.7 V1: Multivariate descriptions: associations of predictors with structural variables

A scatterplot of each predictor with age, with different panels for males and females will be constructed. Associated Spearman correlation coefficients will be computed.

### 2.3.8 V2: Multivariate descriptions: correlation analyses

A matrix of Spearman correlation coefficients between all pairs of predictors will be computed and described numerically as well as by means of a heatmap.

### 2.3.9 VE1: Multivariate descriptions: comparing nonparametric and parametric predictor correlation

A matrix of Pearson correlation coefficients will be computed. Predictor pairs for which Spearman and Pearson correlation coefficients differ by more than 0.1 correlation units will be depicted in scatterplots.

### 2.3.10 VE2: Variable clustering

A variable clustering analysis will be performed to evaluate which predictors are closely associated. A dendrogram groups predictors by their correlation. Scatterplots of pairs of predictors with Spearman correlation coefficients greater than 0.8 will be created.

### 2.3.11 VE3: Redundancy

Variance inflation factors will be computed between the candidate predictors. This will be done for the three possible candidate models, and using all complete cases in the respective candidate predictor sets. Redundancy will further be explored by computing parametric additive models for each predictor in the three candidate models.

# 3. Analysis ready data for IDA

Based on the IDA plan, this section prepares the source data to be analysis ready: read, clean, tidy and transform. This section focuses on the steps prior to IDA (data screening) and the required additions to the **source data** in order to prepare the data. The aim is to produce an analysis ready data set for the research objective.

## 3.1 Analysis ready dataset

The aim of this section and the remaining chapters of the report are to document the steps taken towards transforming the *source* data set to an *analysis ready* data set. These are the steps prior to the IDA analysis plan being executed.

The steps taken in this section are guided by the [data set specification](https://docs.google.com/spreadsheets/d/1Ft5eyenvDnMBoLvJmcBaklfrYcwyW-rkt-ivIkaphdA/edit#gid=2082358047) for the analysis ready data set, which is based on review of the IDA and analysis strategy.

For example, additional meta-data, data derivations and indicator flags are added to the *source* data set.

To support IDA, it is important that we keep track of the changes to the source data including all new modifications, data derivations and transformations. Therefore, we store references to the source data in the data folder after adding additional meta-data for all variables.

The format of the analysis ready data set follows that of the analysis ready [CDISC data model](https://www.cdisc.org/standards).

### 3.1.1 Data set transformations

Important meta-data is added to the data set from the data dictionary. At this stage we could select the variables of interest to take in to the IDA phase by dropping variables we do not check in IDA.

First the source data set and corresponding data dictionary are loaded. The variable names are normalized.

A new variable is created called PARAMCD which stores the abbreviated variable name as a reference. This provides a link between the dictionary and source data.

The source data is then transformed into a *long* format to store the lab specific measurements called ADLB. A long format enables efficient data processing and also allows new transformed variables to be added during the course of IDA. Structural and demographic variables will be stored in a *wide* format in a separate data set named ADSL.

### 3.1.2 Add lab variable meta-data

The lab parameter variable information such as labels and units on to the transformed data are added directly from the data dictionary.

At this point, additional variables and metadata are derived per the analysis plan including:

- Units
- Variable type
- Categories for sex
- rename outcome to be more informative

### 3.1.3 Reorder variables

Select and re-order the variables as per the data set specification.

### 3.1.4 Add informative variable meta-data

Add variable metadata as label attributes.

### 3.1.5 Visual check outcome is correct

Visual check we have not introduced any errors with the outcome variable. First display marginal distribution from source data variable (Table S1).

Table S1. Marginal distribution of the source outcome variable.

| BloodCulture | n |
| --- | --- |
| No | 675550 |
| Yes | 59000 |

Second, display marginal distribution from transformed data variable.

Table S2. Martinal distribution of the transformed data outcome variable.

| Blood culture result for bacteremia (Character coding) | n |
| --- | --- |
| No | 675550 |
| Yes | 59000 |

### 3.1.6 Derive indicator flags

The next step is to derive indicator flags for predictors as per the IDA plan (see [Section 2.2](#sec-IDA_data_derivations)):

- age (AGE), leukocytes (WBC), blood urea neutrogen (BUN), creatinine (CREA), thrombocytes (PLT), and neutrophiles (NEU) and these predictors will be included in the model as key predictors
- Predictors of medium importance are potassium (POTASS), and some acute-phase related parameters such as fibrinogen (FIB), C-reactive protein (CRP), aspartate transaminase (ASAT), alanine transaminase (ALAT), and gamma-glutamyl transpeptidase (GGT).

Next step, add metadata flags to indicate relationship between blood cell parameters. See [Section 2.2](#sec-IDA_data_derivations).

### 3.1.7 Data derivations

Now, derive age groups. For the purpose of stratifying IDA results by age, age will be categorized into the following three groups ([Section 2.2](#sec-IDA_data_derivations)):

- [16, 50],
- (50, 65],
- (65, 101].

### 3.1.8 Save analysis ready data for IDA

Save the analysis data sets in to two linked data sets following a structure similar to the CDISC ADaM data standard. Individual patient measurements are stored in a data set called ADSL. The lab specific data sets are stored in ADLB (a long format data set).

Saving ADSL into an intermediate location DATA/IDA/ADSL_01.rds prior to IDA.

Saving ADLB into an intermediate location DATA/IDA/ADLB_01.rds prior to IDA.

Note: At this stage of IDA, both ADSL and ADLB are *intermediate* files that will be used for further IDA. Findings in IDA may require updates to either data set.

# 4. Results of IDA: Missing values

This section reports the IDA analyses for missing data. The Section headers (i.e. M2) correspond to the IDA analysis plan in [Chapter 2](#sec-IDA_plan).

## 4.1 M1: Participant missingness

As the data was obtained from the registry of the laboratory, and only performed laboratory analyses were included, participant missingness cannot be evaluated.

## 4.2 M2: Variable missingness

Number and percentage of missingness for each predictor, sorted by descending missingness proportion (Tables S3, S4, S5).

### 4.2.1 Outcome and Structural variables

Table S3: Summary of missing values for outcome and structural predictors

| Variable | Missing (count) | Missing (%) |
| --- | --- | --- |
| BACTEREMIA | 0 | 0.0 |
| AGE | 0 | 0.0 |
| SEX | 0 | 0.0 |

### 4.2.2 Lab parameters

Missingness patterns for key and medium importance predictors are reported here. The remaining predictors are reported in Appendix [Section D.1](#sec-M1-appendix) .

Table S4: Summary of missing values for key predictors

| Predictor | Description | Missing (count) | Missing (%) |
| --- | --- | --- | --- |
| NEU | Neutrophiles (G/L) | 728 | 5.0 |
| WBC | White blood count (G/L) | 462 | 3.1 |
| BUN | Blood urea nitrogen (mg/dl) | 172 | 1.2 |
| CREA | Creatinine (mg/dl) | 159 | 1.1 |
| PLT | Blood platelets (G/L) | 42 | 0.3 |
| AGE | Patient Age (years) | 0 | 0.0 |

Table S5: Summary of missing values for medium importance predictors

| Predictor | Description | Missing (count) | Missing (%) |
| --- | --- | --- | --- |
| FIB | Fibrinogen (mg/dl) | 2567 | 17.5 |
| POTASS | Potassium (mmol/L) | 2008 | 13.7 |
| GGT | Gamma-glutamyl transpeptidase (G/L) | 1262 | 8.6 |
| ASAT | Aspartate transaminase (U/L) | 1154 | 7.9 |
| ALAT | Alanin transaminase (U/L) | 987 | 6.7 |
| CRP | C-reactive protein (mg/dl) | 155 | 1.1 |

## 4.3 M3: Complete cases

Number of available complete cases (outcome and predictors, Table S6).

Table S6: Summary of complete cases by sets of predictors

| Set | Complete (count) | Complete (%) |
| --- | --- | --- |
| Outcome | 14691 | 100.0 |
| Outcome and structural variables | 14691 | 100.0 |
| Outcome and key predictors only | 13793 | 93.9 |
| Outcome key predictors and predictors of medium importance | 9389 | 63.9 |
| Outcome and all predictors | 3979 | 27.1 |

## 4.4 M4: Patterns of missing values

### 4.4.1 Complete cases by strata defined by structural variables

The number of complete cases in strata defined by structural variables is described in Table S7.

Table S7: Summary of complete cases by sets of predictors

| Set | # patients | Complete (count) | Complete (%) |
| --- | --- | --- | --- |
| female - [16, 50] | | | |
| All predictors | 2462 | 604 | 24.5 |
| Key predictors | 2462 | 2309 | 93.8 |
| Medium importance predictors | 2462 | 1656 | 67.3 |
| female - (50, 65] | | | |
| All predictors | 1579 | 411 | 26.0 |
| Key predictors | 1579 | 1468 | 93.0 |
| Medium importance predictors | 1579 | 1075 | 68.1 |
| female - (65, 101] | | | |
| All predictors | 2114 | 545 | 25.8 |
| Key predictors | 2114 | 1975 | 93.4 |
| Medium importance predictors | 2114 | 1389 | 65.7 |
| male - [16, 50] | | | |
| All predictors | 2903 | 805 | 27.7 |
| Key predictors | 2903 | 2744 | 94.5 |
| Medium importance predictors | 2903 | 1993 | 68.7 |
| male - (50, 65] | | | |
| All predictors | 2671 | 771 | 28.9 |
| Key predictors | 2671 | 2504 | 93.7 |
| Medium importance predictors | 2671 | 1862 | 69.7 |
| male - (65, 101] | | | |
| All predictors | 2962 | 843 | 28.5 |
| Key predictors | 2962 | 2793 | 94.3 |
| Medium importance predictors | 2962 | 2014 | 68.0 |

### 4.4.2 Dendrogram of missingness indicators

Figure S1 depicts the results of a cluster analysis using the complete linkage method based on the percentage of discordant missing indicators. (This percentage was computed via the squared Euclidian distance of missingness indicators between predictors.) The horizontal axis shows the distance between two clusters, which is given by the maximum distance between any element of the first and the second clusters. For example, if two clusters are merged at a height of 25 it means that in 25% of the observations the missingness indicators of the most discordant predictors contained in the two clusters are discordant.

Figure S1. Dendrogram of missingness. See text for explanation of the horizontal axis. The numbers in brackets are the percentages of missing observations for each predictor.


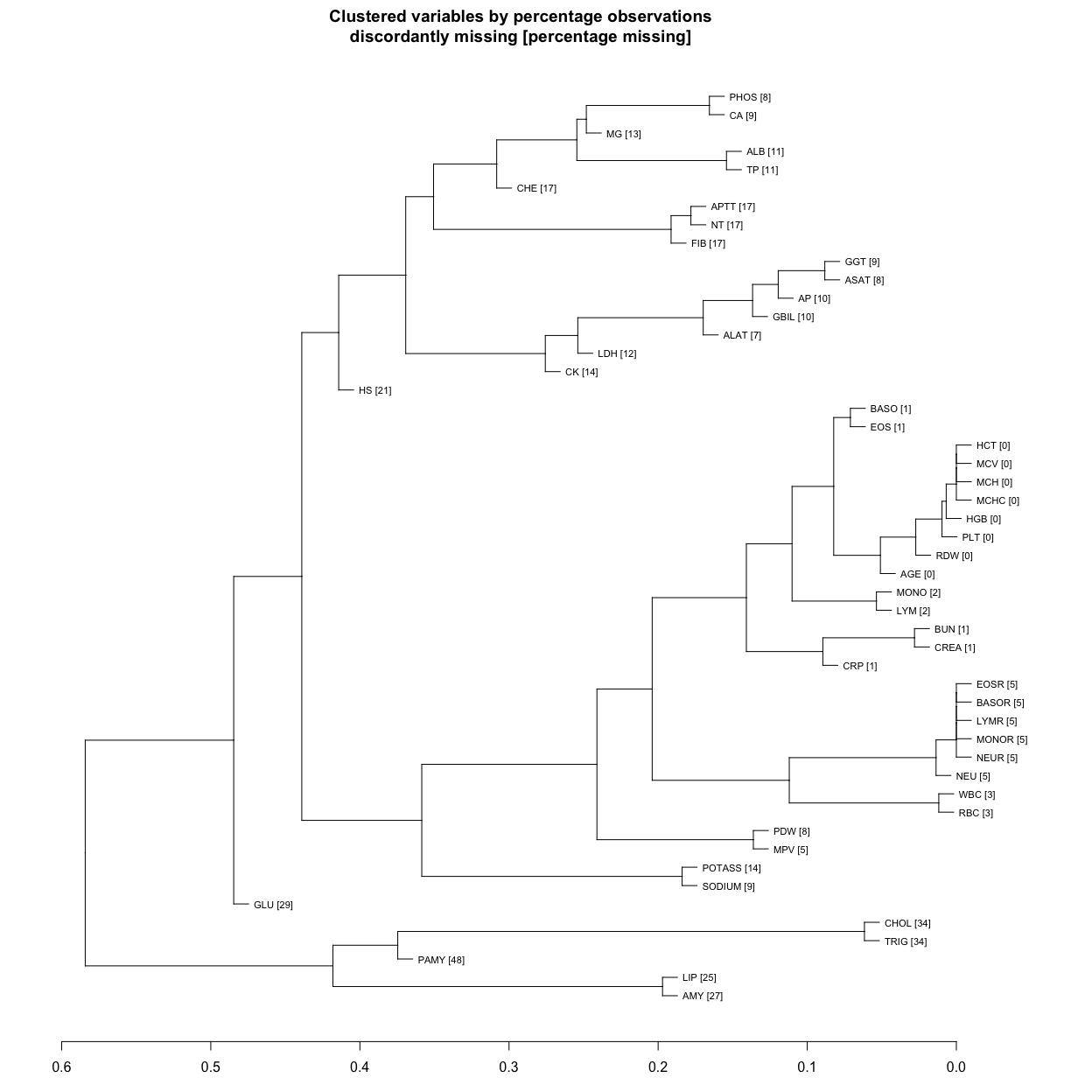


# 5. Univariate distribution checks

This section reports a series of univariate summary checks of the bacteremia dataset.

## 5.1 U1: Categorical variables

Age group, sex and bacteremia status are described in Table S8 by frequencies and proportions in each category.

Table S8: Summary of categorical variables including outcome.

| Category | Count | Proportion |
| --- | --- | --- |
| Age group | | |
| [16, 50] | 5365 | 0.37 |
| (65, 101] | 5076 | 0.35 |
| (50, 65] | 4250 | 0.29 |
| Sex | | |
| male | 8536 | 0.58 |
| female | 6155 | 0.42 |
| Presence of bacteremia | | |
| no | 13511 | 0.92 |
| yes | 1180 | 0.08 |

Also plot the categories as simple bar charts.

Figure S2: Graphical summary of categorical variables including outcome as bar charts.

| 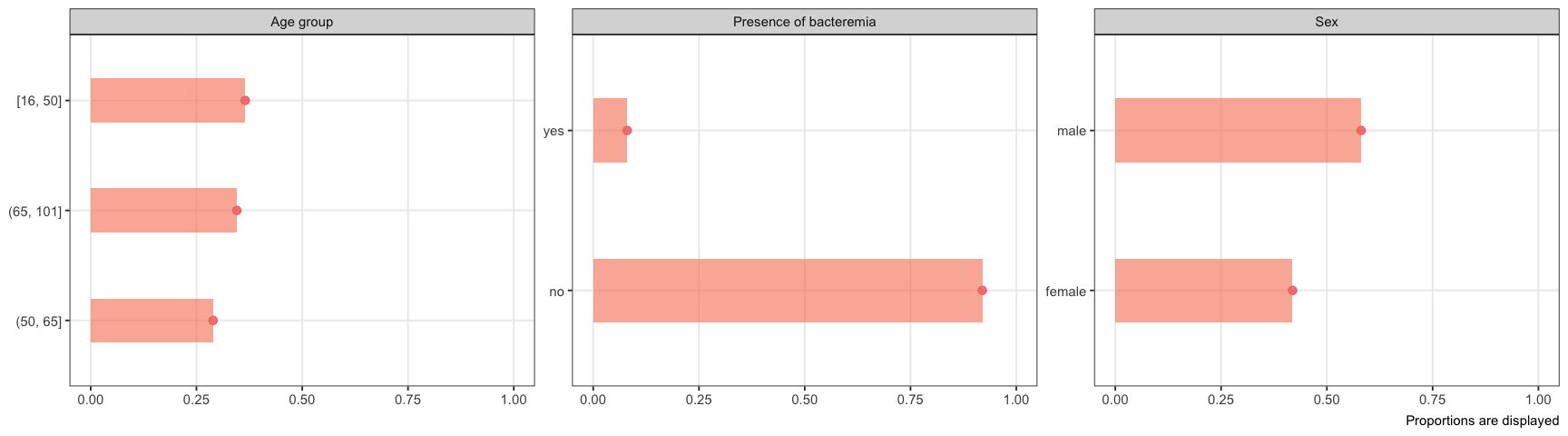 |
| --- |

## 5.2 Continuous variables

### 5.2.1 U2: Univariate distributions of continuous variables

#### 5.2.1.1 U2: Structural variables

The only structural continuous variable is AGE. This variable is also a key predictor and reported in the following section (see below).

#### 5.2.1.2 U2: Key predictors

Distribution of key predictors. Lines indicate the 5-number summary including reported numerical values (where possible).

The remaining predictors are reported in the appendix [Section E.1.1](#sec-u2-remaining).

Figure S3. Univariate distributions of key predictors (histograms). Vertical bars describe minimum, 25th, 50th, and 75th percentiles, and maximum.

| 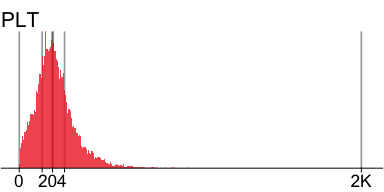 | 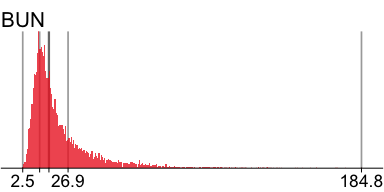 | 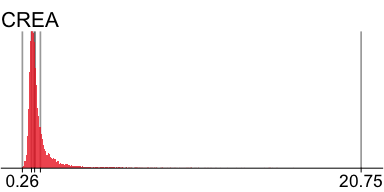 |
| --- | --- | --- |

| 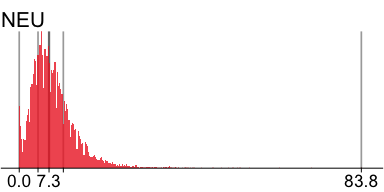 | 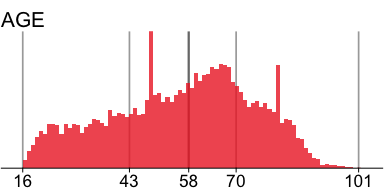 | 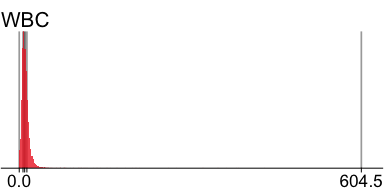 |
| --- | --- | --- |

#### 5.2.1.3 U2: Predictors of medium importance

Figure S4. Univariate distributions of predictors of medium importance (histograms). Vertical bars describe minimum, 25th, 50th, and 75th percentiles, and maximum.

| 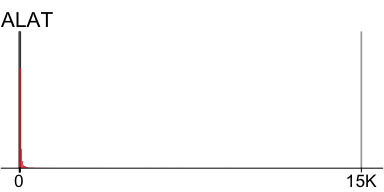 | 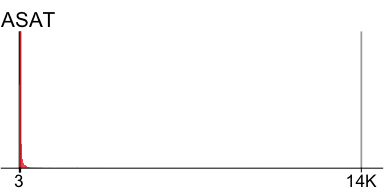 | 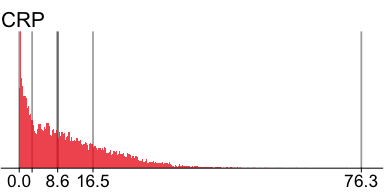 |
| --- | --- | --- |

| 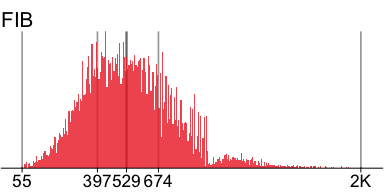 | 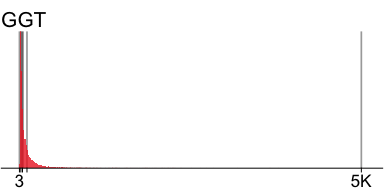 | 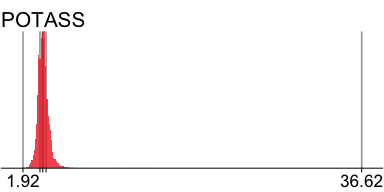 |
| --- | --- | --- |

###

### 5.2.2 Numerical summaries

#### 5.2.2.1 Key predictors

Figure S5: Combined numerical and graphical summaries of key predictors.


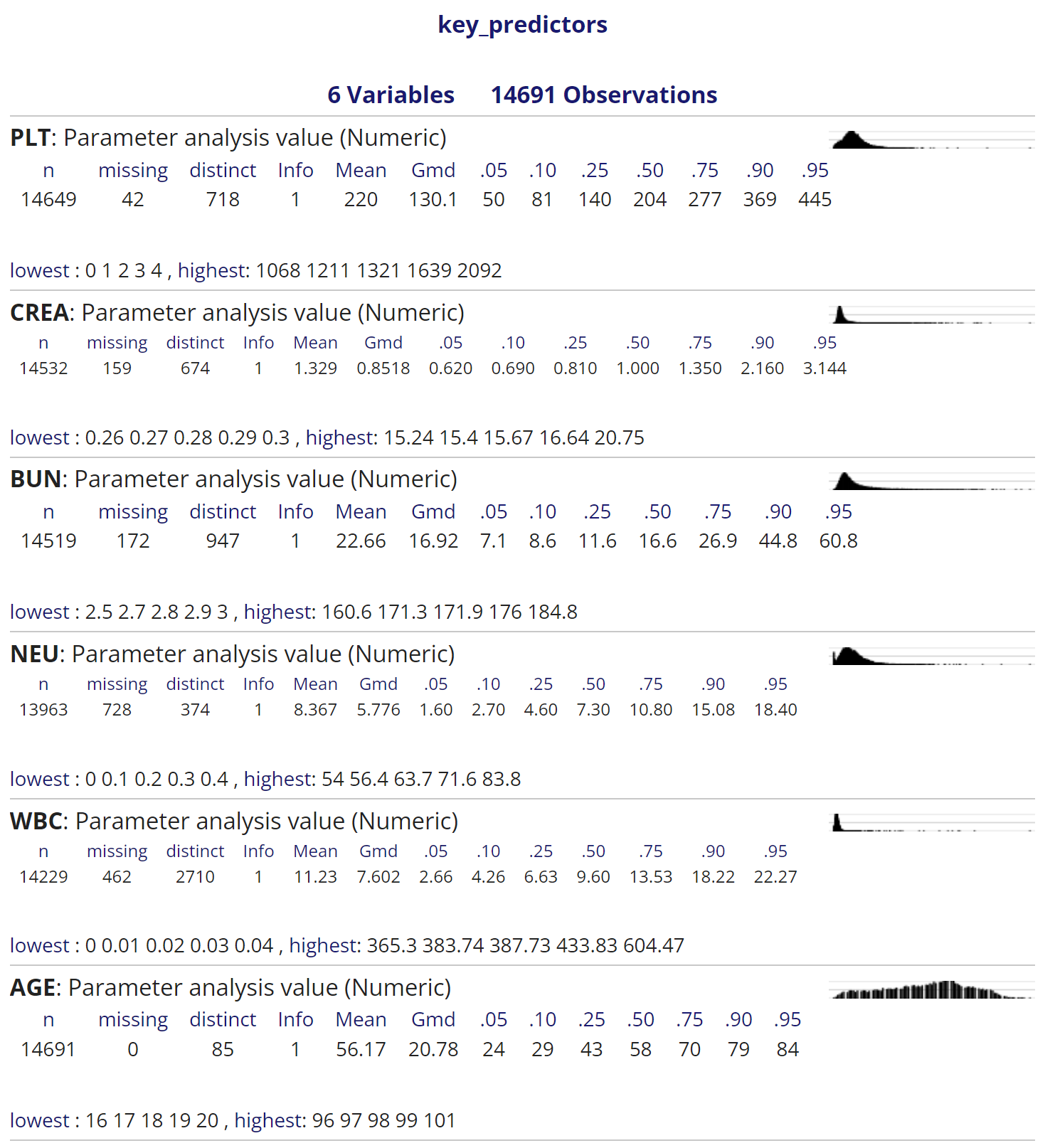


#### 5.2.2.2 Predictors of medium importance

Figure S6: Combined numerical and graphical summaries of predictors of medium importance

###
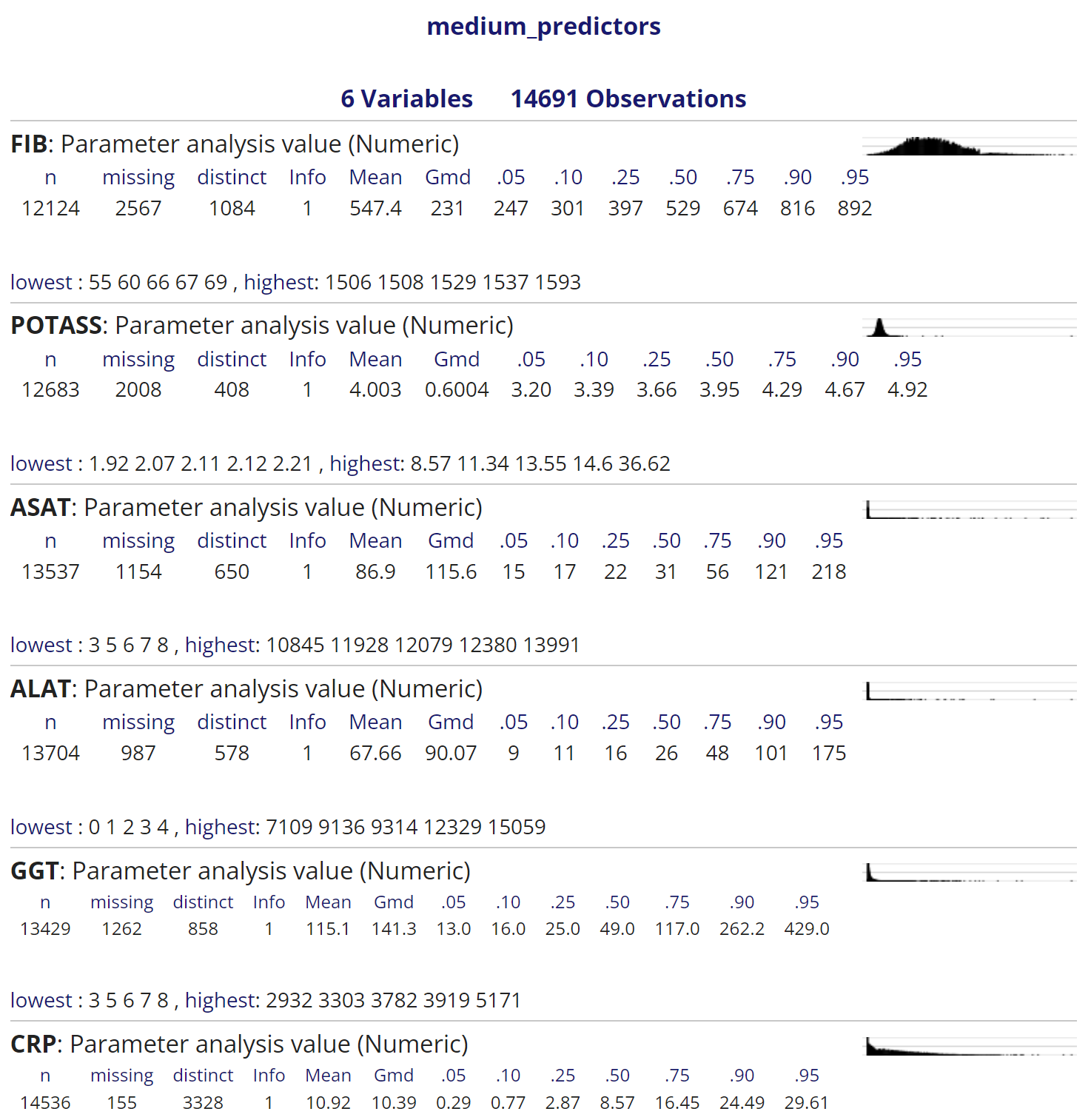


### 5.2.3 Suggested transformations

Next we investigate whether a pseudolog transformation of continuous variables may substantially symmetrize the univariate distributions of the continuous variables, and may hence be useful for multivariate summaries. We employ a function ida_trans for this purpose, which optimises the parameter sigma of the pseudo-logarithm for that purpose. The optimization targets the best possible linear correlation of the transformed values with normal deviates. If no better transformation can be found, or if the improvement in correlation is less than 0.2 correlation units, no transformation is suggested.

Display the proposed variable transformations and the new parameter codes.

Table S9. Predictors for which a transformation was proposed.

| PARAMCD | n |
| --- | --- |
| ALAT_T | 14691 |
| AMY_T | 14691 |
| AP_T | 14691 |
| ASAT_T | 14691 |
| BASO_T | 14691 |
| CK_T | 14691 |
| CREA_T | 14691 |
| EOS_T | 14691 |
| GBIL_T | 14691 |
| GGT_T | 14691 |
| LDH_T | 14691 |
| LIP_T | 14691 |
| LYM_T | 14691 |
| PAMY_T | 14691 |
| WBC_T | 14691 |

Register transformed variables in the data set. The updated data set with suggested log transformed data sets is saved at data/IDA/ADLB_02.rds.

The IDA analysis plan and specifications are updated with the proposed variable transformations. A new flag is derived to indicate the categorization of predictors now including transformations.

### 5.2.4 Comparison of univariate distributions with and without pseudo-log transformation

The comparison is only shown for variables where a transformation is suggested (Figure S7. Note, all observed values, and the distribution min, max and interquartile range as reference lines, are displayed.

Figure S7. Histograms of original and transformed predictors. The vertical bars describe the minimum, 25th, 50th and 75th percentiles, and maximum.

| 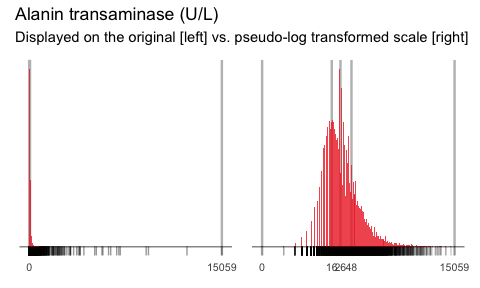 | 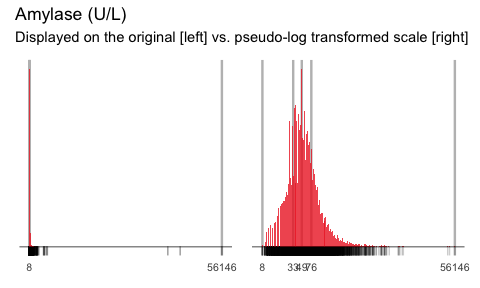 |
| --- | --- |

| 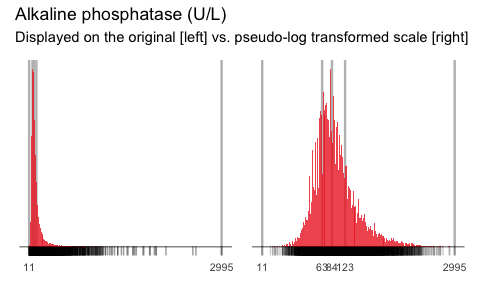 | 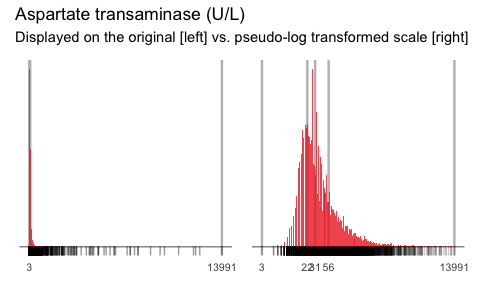 |
| --- | --- |

| 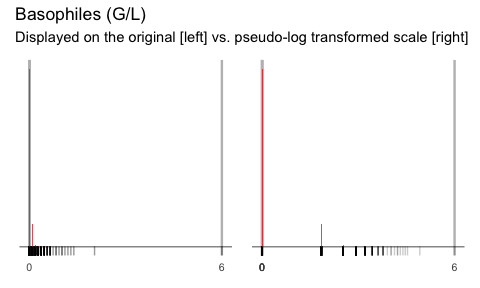 | 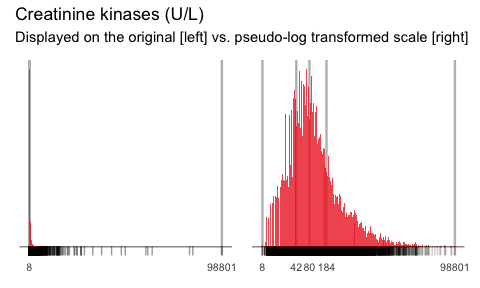 |
| --- | --- |

| 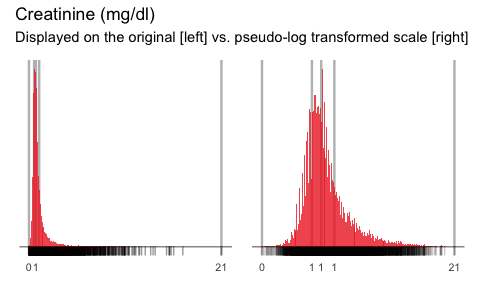 | 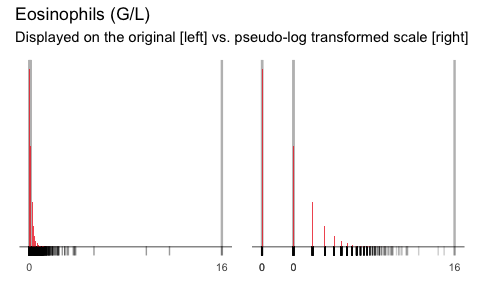 |
| --- | --- |

Figure S7 (continued). Histograms of original and transformed predictors. The vertical bars describe the minimum, 25th, 50th and 75th percentiles, and maximum.

| 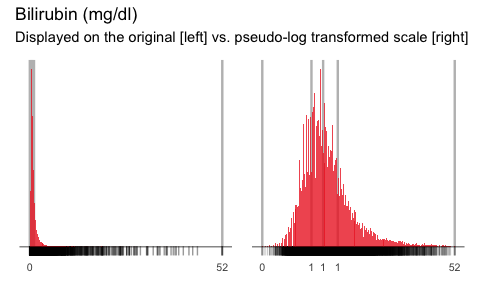 | 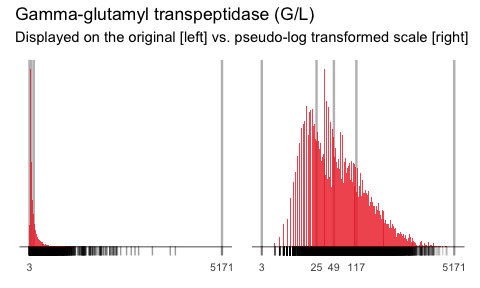 |
| --- | --- |

| 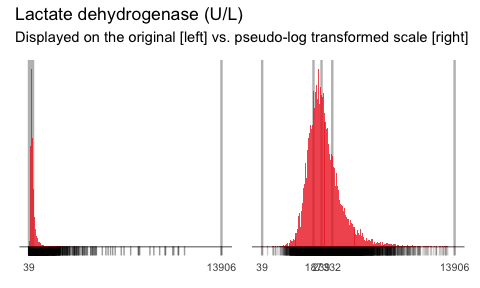 | 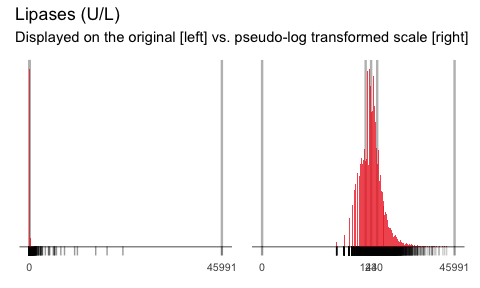 |
| --- | --- |

| 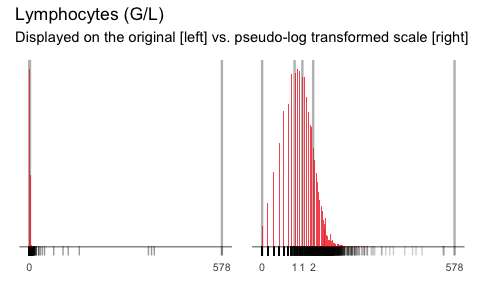 | 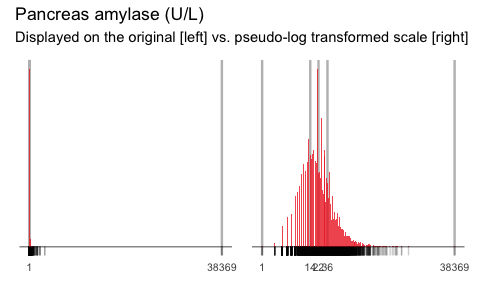 |
| --- | --- |

| 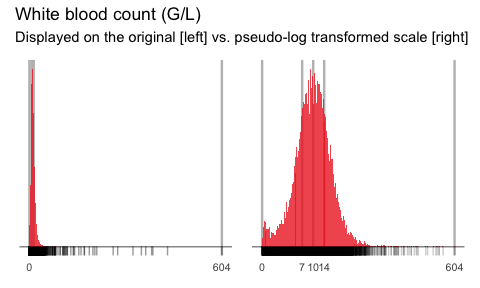 |
| --- |

# 6. Multivariate analyses

First load the required packages and data. Note that from the univariate analyses the analysis data set for the lab parameters has been updated to include transformed variables. Therefore, we load the second iteration of the data data/ADLB_02.rds.

## 6.1 V1: Association with structural variables

Attached the required structural variables to the lab data.

A scatterplot of each predictor with age, with different panels for males and females have been constructed (Figures S8, S9). Associated Spearman correlation coefficients have been computed.

The remaining predictors are reported in appendix [Section F.1](#sec-V1-appendix).

### 6.1.1 Key predictors

Figure S8. Scatterplots of key predictors and age, subgrouped by sex.

| 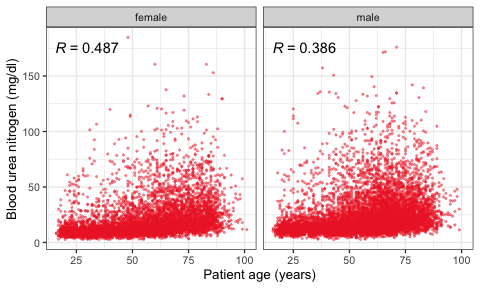 | 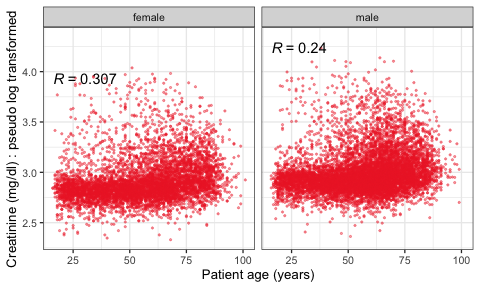 |
| --- | --- |

| 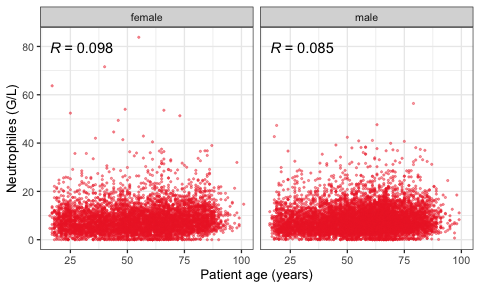 | 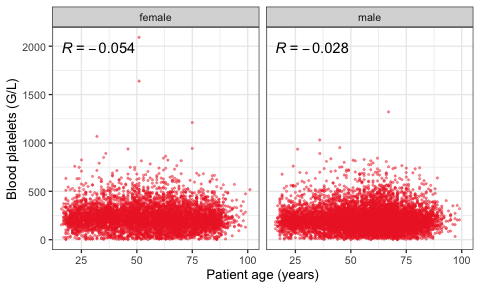 |
| --- | --- |

| 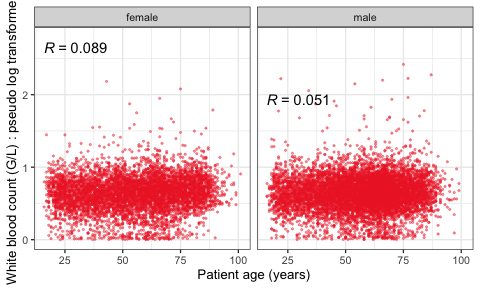 |
| --- |

###

### 6.1.2 Predictors of medium importance

Figures S9. Scatterplots of predictors of medium importance and age, subgrouped by sex.

| 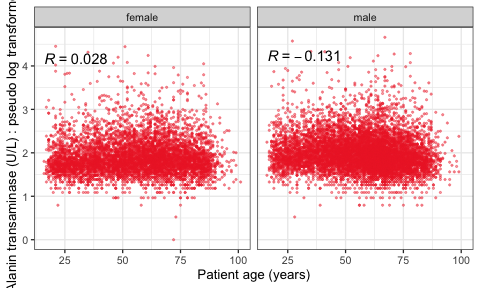 | 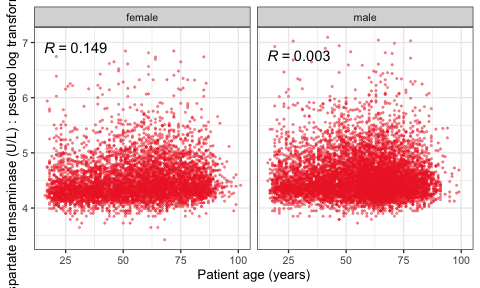 |
| --- | --- |

| 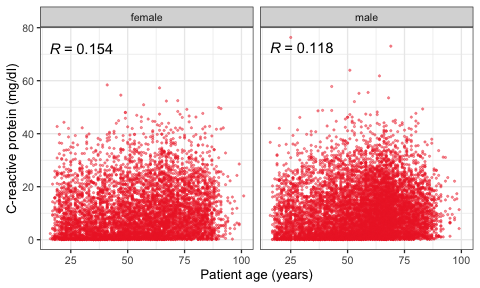 | 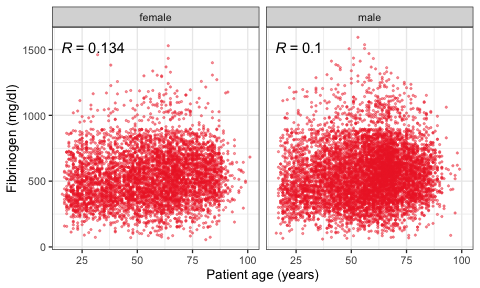 |
| --- | --- |

| 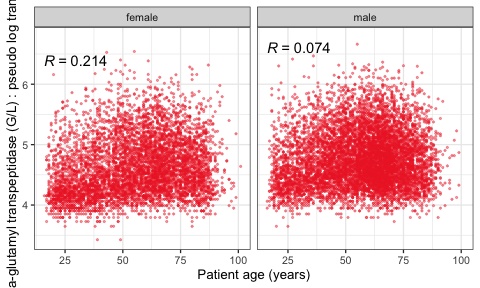 | 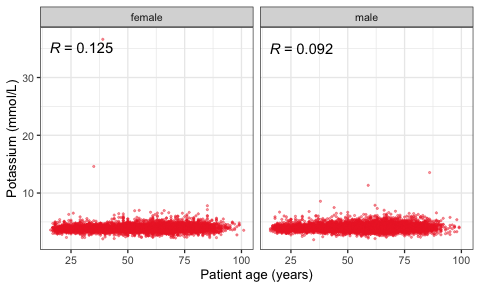 |
| --- | --- |

##

## 6.2 V2: Correlation coefficients between all predictors

Calculate correlation matrix using Spearman correlation coefficient.

The Spearman correlation coefficients are depicted in a quadratic heat map (Figure S10).

Figure S10. Spearman correlation plot of all predictors.


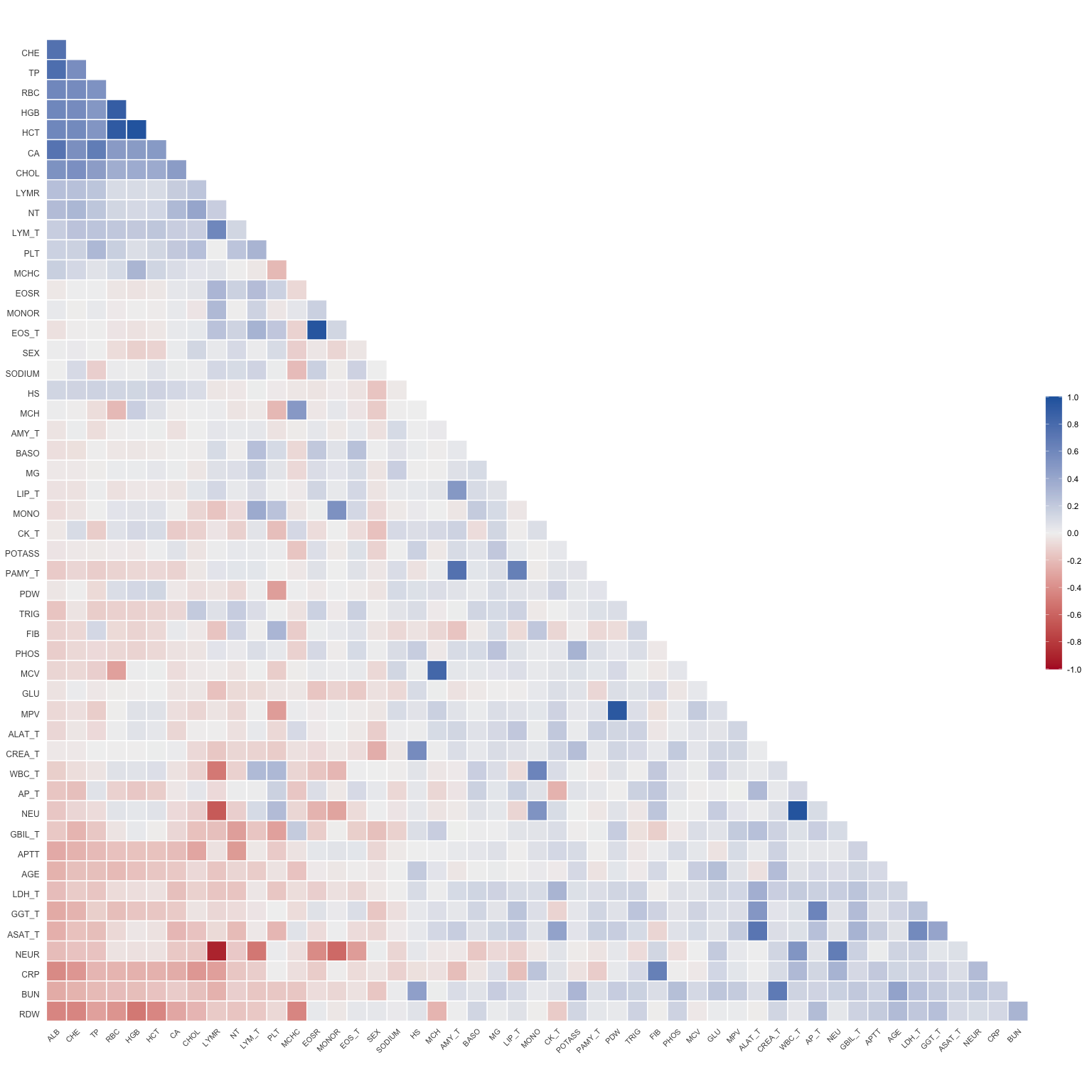


### 6.2.1 VE1: Comparing nonparametric and parametric predictor correlation

Plot the matrix of differences between Spearman and Pearson pairwise correlation coefficients.

Figure S11. Plot of differences between Spearman and Pearson correlation coefficients for all predictors.


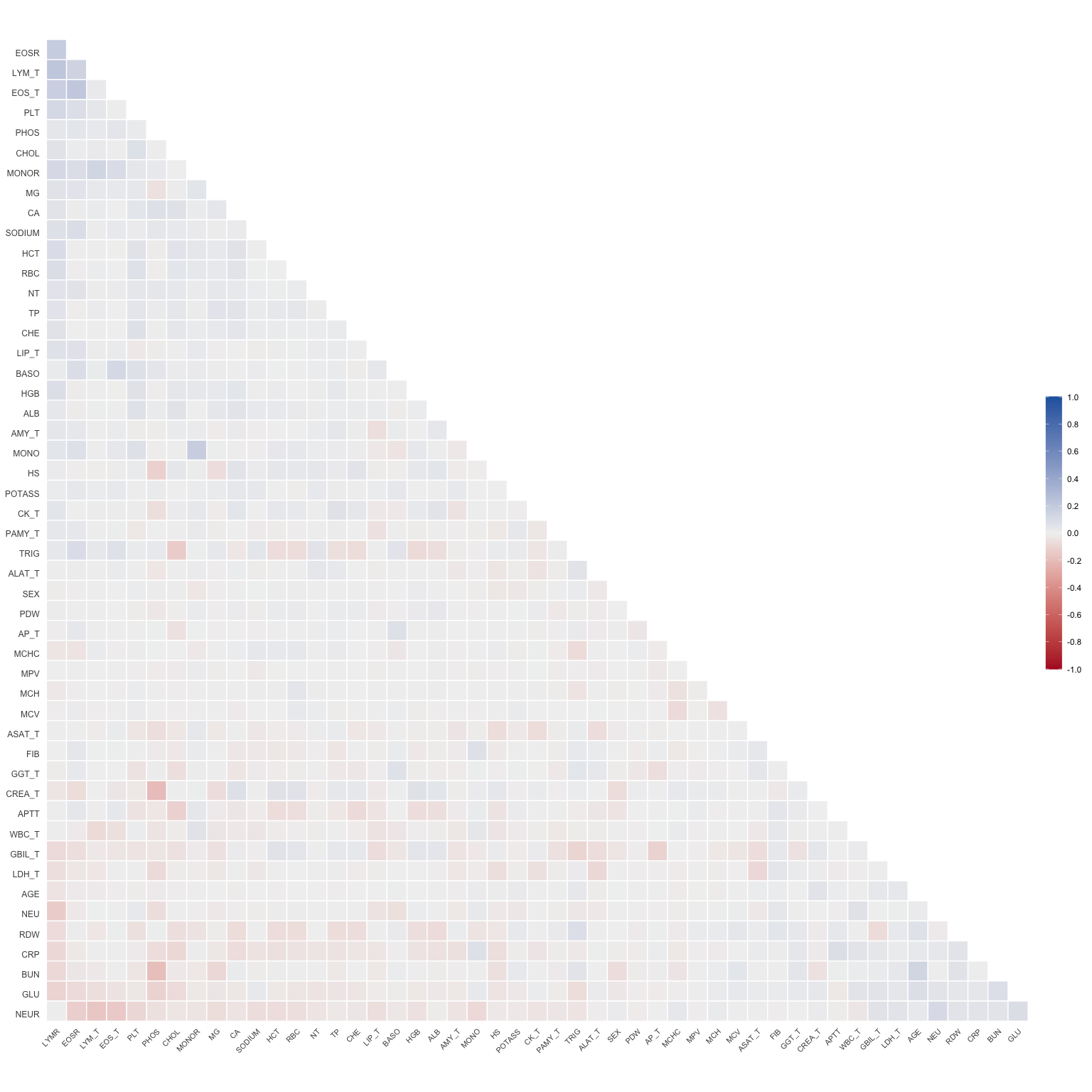


Plot the matrix of differences between Spearman and Pearson pairwise correlation coefficients but suppress differences less then 0.1 in absolute value.

Figure S12. Plot of differences between Spearman and Pearson correlation coefficients for all predictors, with absolute differences less than 0.1 suppressed.


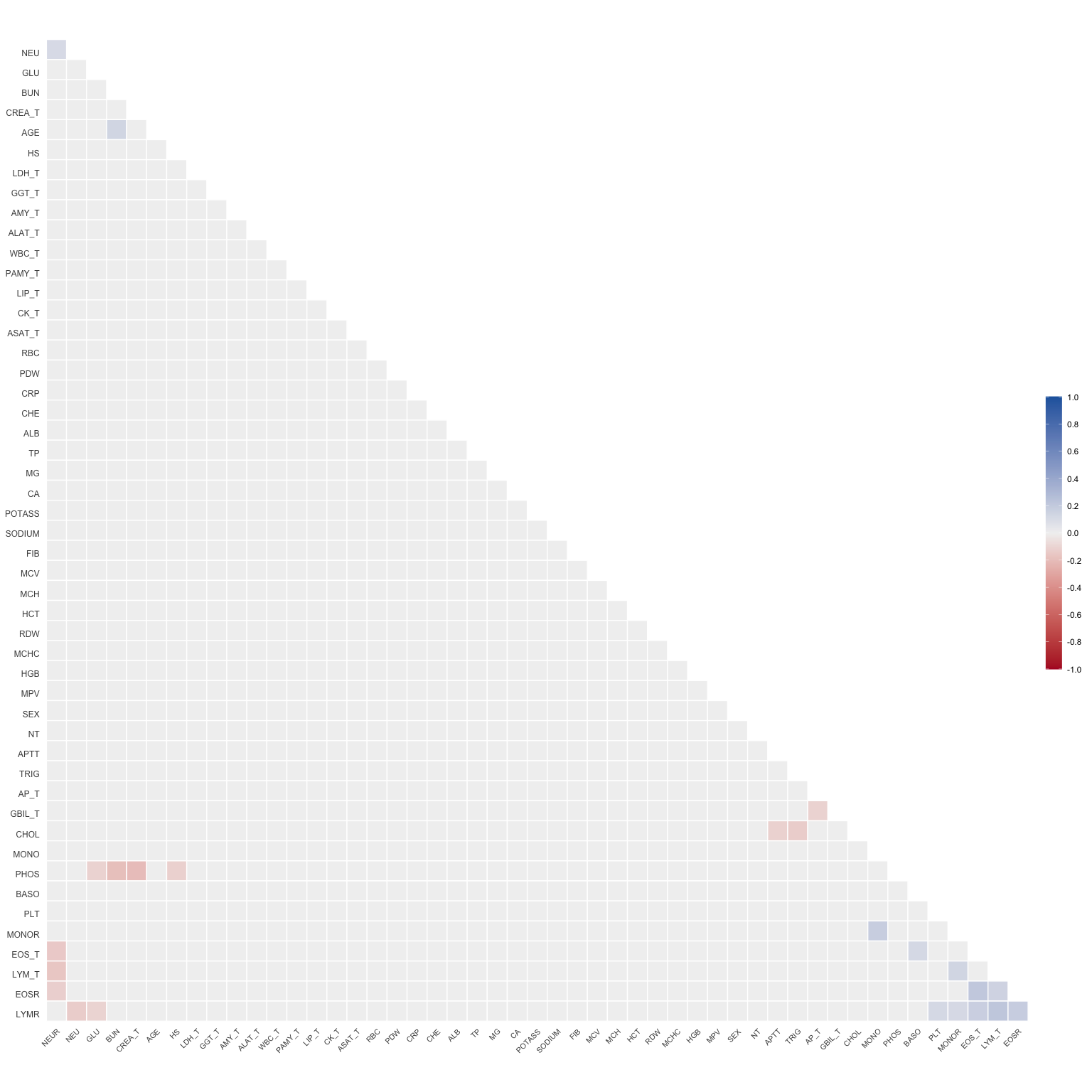


Predictor pairs for which Spearman and Pearson correlation coefficients differ by more than 0.1 correlation units in absolute value will be depicted in scatterplots. First report the table of predictor pairs where the difference (column r) is greater than 0.1.

Table S10. Predictor pairs where the absolute difference between Spearman and Pearson correlation coefficients is greater than 0.1 correlation units.

| x | y | r |
| --- | --- | --- |
| PLT | LYMR | 0.12 |
| MONO | MONOR | 0.18 |
| BASO | EOS_T | 0.12 |
| BUN | AGE | 0.13 |
| EOSR | LYMR | 0.18 |
| EOSR | EOS_T | 0.21 |
| EOSR | LYM_T | 0.14 |
| LYMR | MONOR | 0.11 |
| LYMR | EOS_T | 0.17 |
| LYMR | LYM_T | 0.22 |
| MONOR | LYM_T | 0.13 |
| NEU | NEUR | 0.11 |

Then plot the scatter plots (Figure S13).

Figure S13. Scatter plots of predictor pairs with absolute difference between Spearman and Pearson correlation coefficients greater than 0.1 correlation units.

| 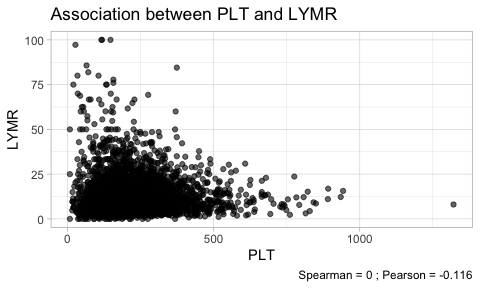 | 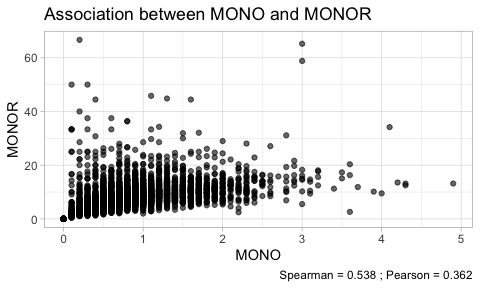 | 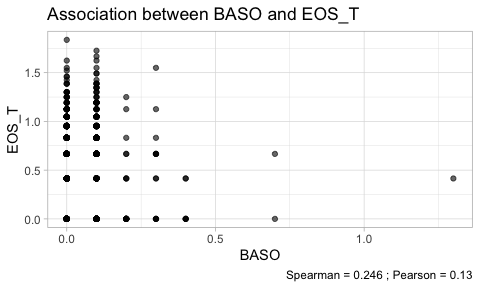 |
| --- | --- | --- |

| 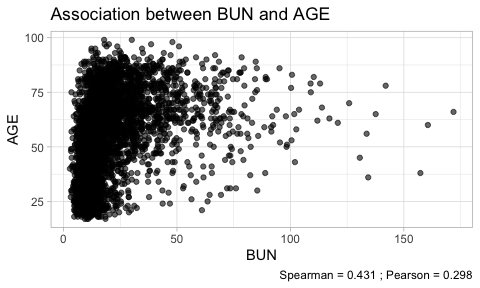 | 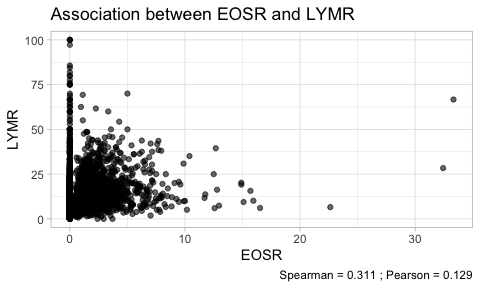 | 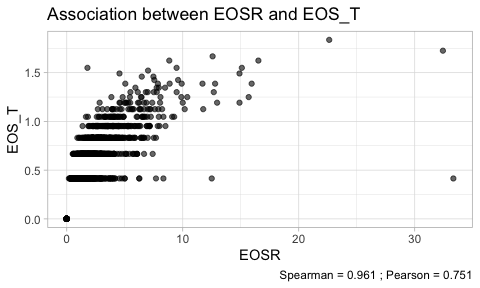 |
| --- | --- | --- |

| 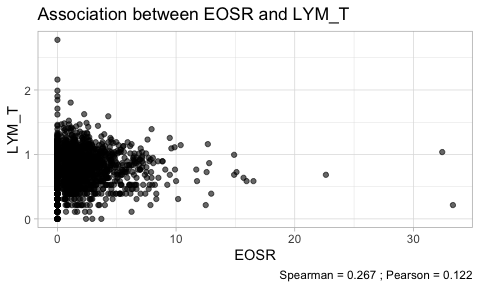 | 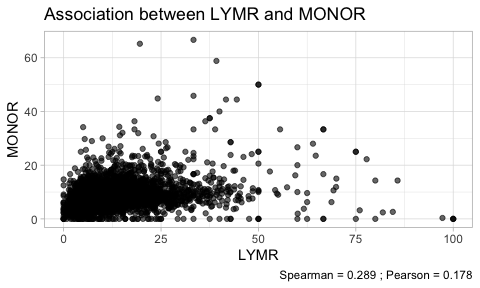 | 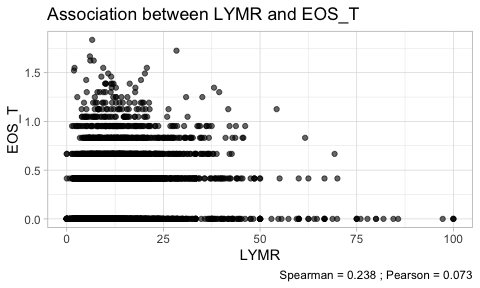 |
| --- | --- | --- |

| 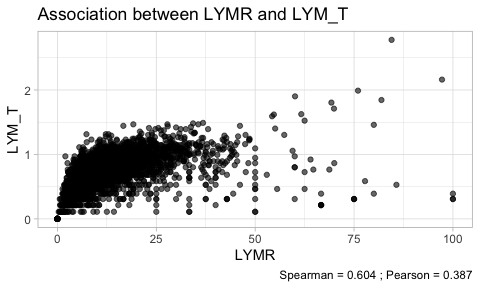 | 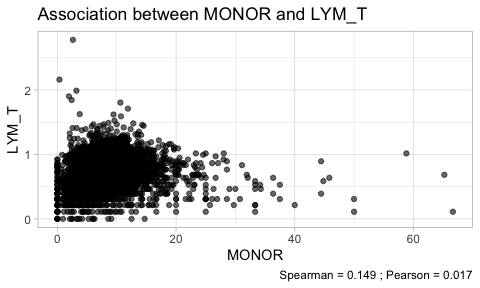 | 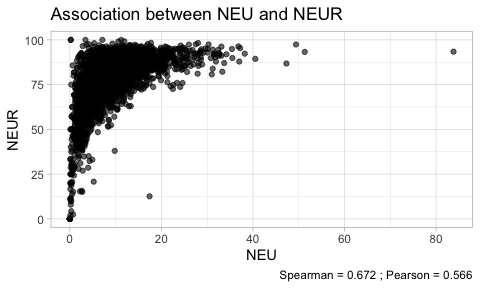 |
| --- | --- | --- |

###

### 6.2.2 VE2: Variable clustering

A variable clustering analysis has been performed to evaluate which predictors are closely associated. The dendrogram groups predictors by their correlation (Figure S14).

Figure S14. Dendrogram showing clusters of predictors by their correlation.


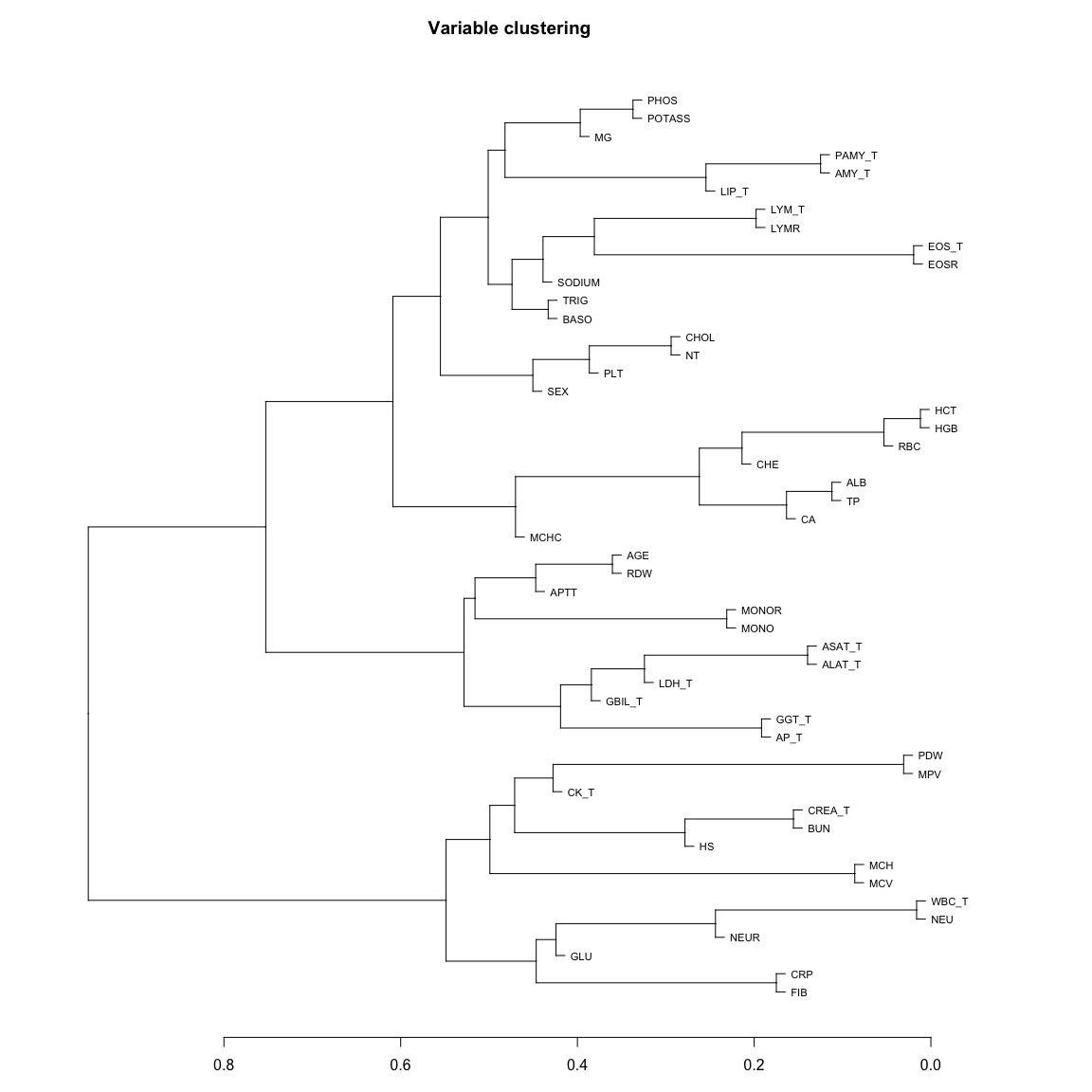


This can also be displayed as a network plot (Figure S15).

Figure S15: Network plot of predictors. The edges in the network describe the strength of correlation.


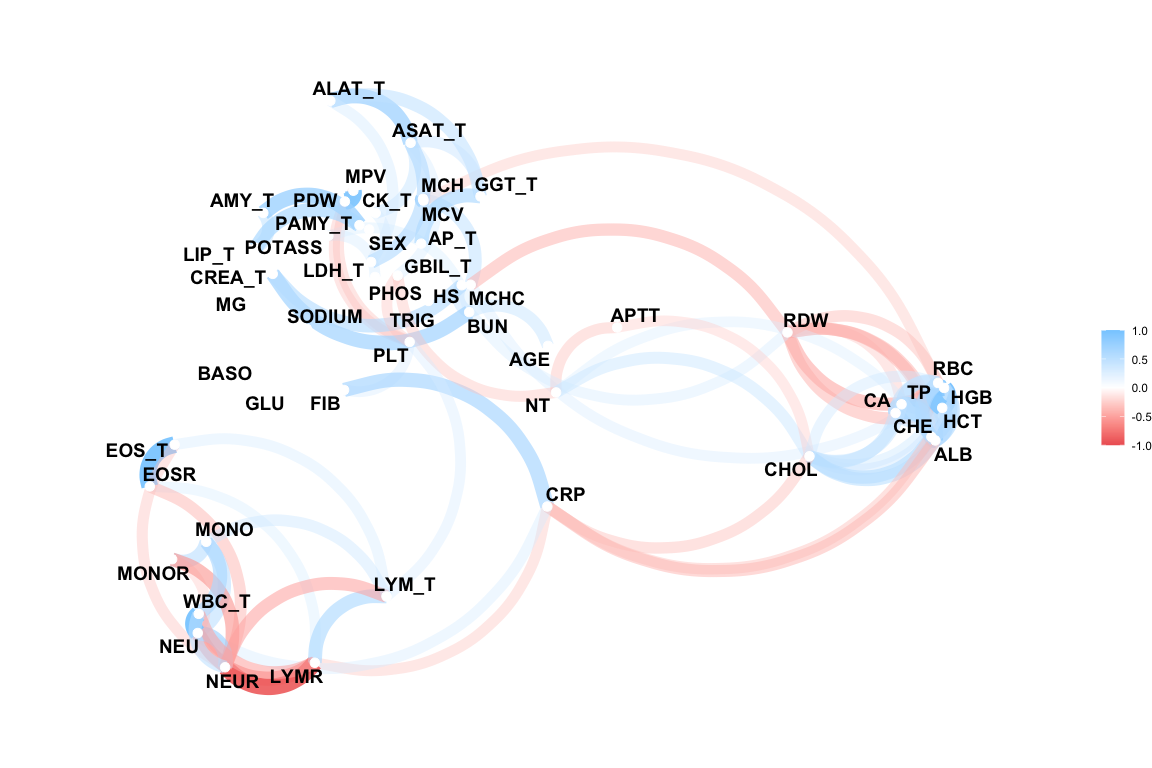


In the scatterplots of Figure S16 we show predictor pairs with Spearman correlation coefficients greater than 0.8.

Figure S16. Predictor pairs with absolute Spearman correlation coefficients greater than 0.8.

| 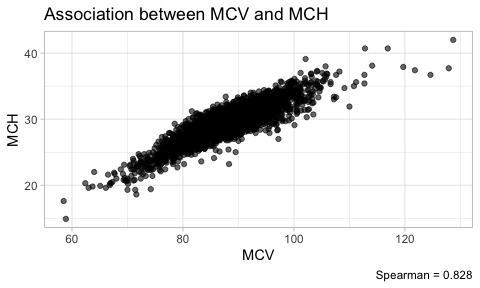 | 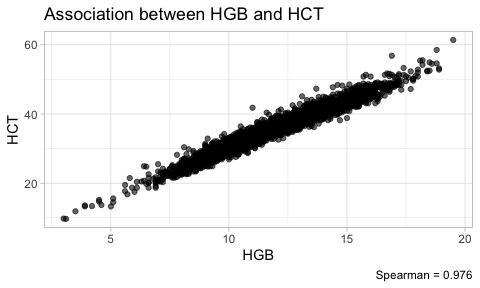 |
| --- | --- |

| 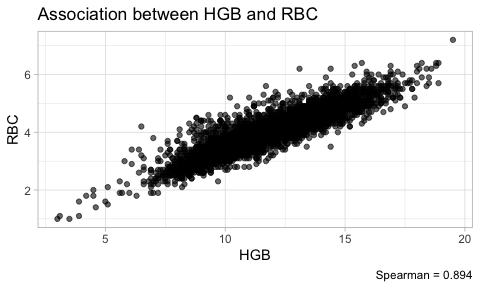 | 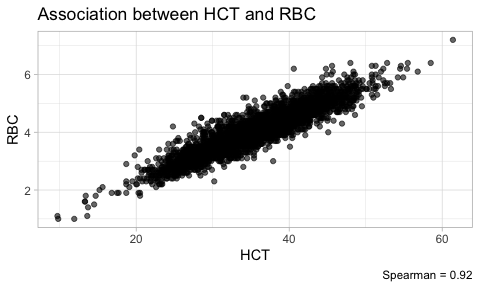 |
| --- | --- |

| 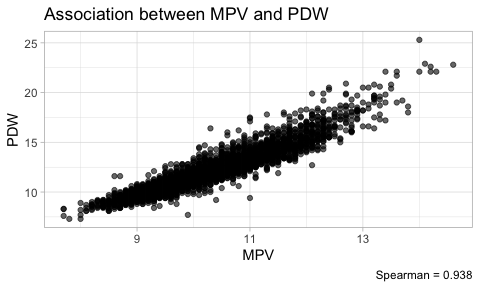 | 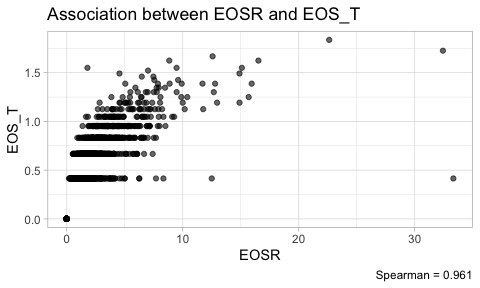 |
| --- | --- |

| 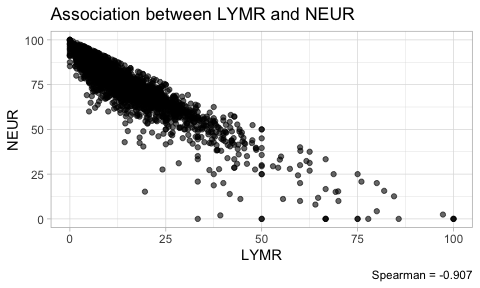 | 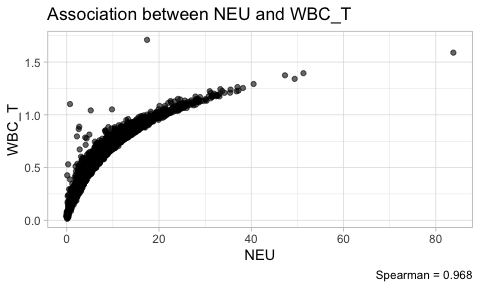 |
| --- | --- |

### 6.2.3 VE3: Redundancy

Variance inflation factors (VIF) will be computed between the candidate predictors. This will be done for the three possible candidate models, and using all complete cases in the respective candidate predictor sets. Since $VIF=\left( 1-R^{2} \right)^{-1}$, we also report the multiple R-squared values.

Redundancy was further explored by computing parametric additive models for each predictor in the key predictor model and the extended predictor model. VIFs and multiple $R^{2}$ are reported from those models, again for the three predictor sets.

Note, the all predictor model is reported in appendix [Section F.2](#sec-VE3-appendix).

#### 6.2.3.1 VIF for key predictor model

Table S11. Variance inflation factors and multiple R-squared of key predictors. The available sample size is 13793 (93.89 %).

| Parameter code | Variance inflation factor | Multiple R-squared |
| --- | --- | --- |
| SEX | 1.1 | 0.05 |
| PLT | 1.2 | 0.15 |
| BUN | 2.4 | 0.58 |
| NEU | 5.4 | 0.82 |
| AGE | 1.1 | 0.09 |
| CREA_T | 2.3 | 0.56 |
| WBC_T | 5.6 | 0.82 |

####

#### 6.2.3.2 VIF for model with key predictors and predictors of medium importance

Table S12. Variance inflation factors and multiple R-squared for key predictors and predictors of medium importance. The available sample size is 9389 (63.91 %).

| Parameter code | Variance inflation factor | Multiple R-squared |
| --- | --- | --- |
| SEX | 1.1 | 0.09 |
| PLT | 1.4 | 0.30 |
| FIB | 2.3 | 0.56 |
| POTASS | 1.1 | 0.10 |
| BUN | 2.6 | 0.61 |
| CRP | 2.2 | 0.54 |
| NEU | 5.9 | 0.83 |
| AGE | 1.2 | 0.15 |
| ALAT_T | 3.3 | 0.69 |
| ASAT_T | 3.2 | 0.69 |
| CREA_T | 2.4 | 0.58 |
| GGT_T | 1.4 | 0.30 |
| WBC_T | 6.0 | 0.83 |

#### 6.2.3.3 VIF for all predictor model

See appendix [Section F.2](#sec-VE3-appendix).

#### 6.2.3.4 Redundancy by parametric additive model: key predictor model

Table S13. Variance inflation factors and multiple R-squared for key predictors in parametric additive models. The available sample size is 13793 (93.89 %).

| Parameter code | Variance inflation factor | Multiple R-squared |
| --- | --- | --- |
| SEX | 1.1 | 0.11 |
| PLT | 1.2 | 0.16 |
| BUN | 2.6 | 0.61 |
| NEU | 11.5 | 0.91 |
| AGE | 1.3 | 0.21 |
| CREA_T | 2.4 | 0.58 |
| WBC_T | 13.7 | 0.93 |

####

#### 6.2.3.5 Redundancy by parametric additive model: key predictors and predictors of medium importance

Table S14. Variance inflation factors and multiple R-squared for key predictors and predictors of medium importance in parametric additive models. The available sample size is 9389 (63.91 %).

| Parameter code | Variance inflation factor | Multiple R-squared |
| --- | --- | --- |
| SEX | 1.2 | 0.15 |
| PLT | 1.5 | 0.32 |
| FIB | 2.4 | 0.58 |
| POTASS | 1.1 | 0.11 |
| BUN | 2.8 | 0.65 |
| CRP | 2.3 | 0.56 |
| NEU | 14.1 | 0.93 |
| AGE | 1.4 | 0.29 |
| ALAT_T | 3.4 | 0.70 |
| ASAT_T | 3.5 | 0.71 |
| CREA_T | 2.5 | 0.60 |
| GGT_T | 1.5 | 0.35 |
| WBC_T | 16.4 | 0.94 |

# 7. Final Analysis ready data

## 7.1 Final derivations and transformations

From the IDA exercise a decision was made to derive a new variable based on White Blood Count in the absence of Neutrophiles:

- WBC_NEU: WBC - NEU

The new derivation will also be checked as a candidate for pseudo-log transformation.

Figure S17. Histograms of original predictor WBC and transformed predictor WBC_NEU. The vertical bars describe the minimum, 25th, 50th, and 75th percentiles, and maximum. The ticks on the x-axis describe observed data points.


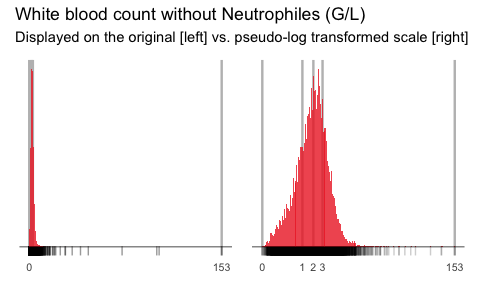


A visual check confirms that a pseudo-log transformation is appropriate for the newly derived variable.

The new derivation and transformation is added to the analysis ready data, setting the correct indicator flags to enable final analyses and modelling.

Visual check of indicator flag and new derivation added to the analysis data set.

[1] "c(\"PLT\", \"CREA\", \"BUN\", \"NEU\", \"AGE\", \"WBC_noNEU\")"

## 7.2 Save final analysis ready data sets

The final analysis ready data sets are then stored in data/Analysis.

# References

Johnson, N. L. 1949. “Systems of Frequency Curves Generated by Methods of Translation.” *Biometrika* 36 (1/2): 149–76. <http://www.jstor.org/stable/2332539>.

Ratzinger, Franz, Michel Dedeyan, Matthias Rammerstorfer, Thomas Perkmann, Heinz Burgmann, Athanasios Makristathis, Georg Dorffner, Felix Lötsch, Alexander Blacky, and Michael Ramharter. 2014. “A Risk Prediction Model for Screening Bacteremic Patients: A Cross Sectional Study.” *PLOS ONE* 9 (9): 1–10.

<https://doi.org/10.1371/journal.pone.0106765>.

# Appendix A — Pseudo-log transformations

## A.1 Introduction

This supplemental section illustrates how pseudo-log transformations can be used to transform skewed distributions towards normality.

The transformation $f\left( x \right)=sinh^{-}1\left( x/2\sigma\right)/log\left( 10 \right)$ is a pseudo-logarithmic transformation mentioned by (Johnson 1949). It has the following advantages over ordinary logarithmic transformations:

- it is defined also for $x=$ as $f\left( 0 \right)=0$
- it is a signed logarithmic transformation, and is defined also for negative values as $f\left( -\left| x \right| \right)=-f\left( \left| x \right| \right)$

Of course, this comes at the cost of deviation from the logarithmic transformation in terms of interpretability.

The parameter $\sigma$ may be used to adapt the transformation to a specific range of an empirical distribution.

We define the pseudo-logarithmic transformation in R as:

pseudo_log <-
 function(x, sigma = 1, base = 10)
 asinh(x / (2 * sigma)) / log(base)

inv_pseudo_log <-
 function(x, sigma = 1, base = 10)
 2 * sigma * sinh(x * log(base))

## A.2 Impact of choice of $\boldsymbol{\sigma}$

Next, we investigate how the parameter $\sigma$ impacts the result of the transformation. We assume that $x$ follows a log-normal distribution, and we will show results of $f\left( x \right)$ with different choices of $\sigma$. We center and scale $f\left( x \right)$ before plotting.

p <- seq(0.001, 0.999, 0.001)
x <- exp(qnorm(p, mean = 0, sd = 1))

hist(x)

Figure S18. Histogram of a simulated log-normally distributed variable x.


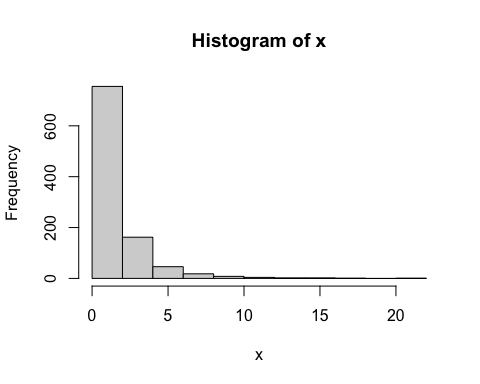


y <-
 cbind(
 log(x),
 scale(pseudo_log(x, 1)),
 scale(pseudo_log(x, 0.5)),
 scale(pseudo_log(x, 2)),
 scale(pseudo_log(x, 0.05))
 )

plot(x,
 y[, 2],
 type = "l",
 ylab = "f(x)",
 ylim = range(y))
lines(x, y[, 1], col = "red")
lines(x, y[, 3], type = "l", lty = 2)
lines(x, y[, 4], type = "l", lty = 3)
lines(x, y[, 5], type = "l", lty = 4)
legend(
 "topleft",
 lty = c(1, 1, 2, 3, 4),
 col = c("red", "black", "black", "black", "black"),
 legend = c("log", "sigma=1", "sigma=0.5", "sigma=2", "sigma=0.05")
)

Figure S19. Logarithmic and pseudo-log transformations of x using several values for the hyperparameter sigma of the pseudo-log transformation. Horizontal axis: original values of x. Vertical axis: transformations f(x).


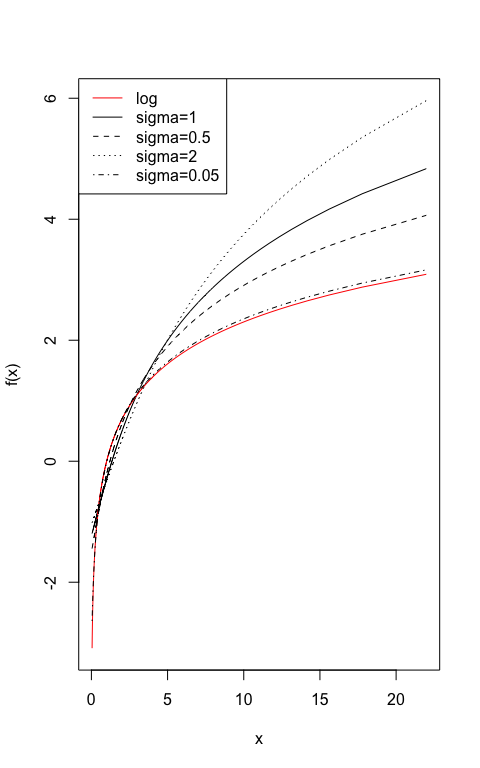


In the next code chunk, we multiply by 10 and repeat the exercise. We learn that the transformations become more similar and the choice of $\sigma$ less relevant.

x <- x * 10

y <-
 cbind(
 scale(log(x)),
 scale(pseudo_log(x, 1)),
 scale(pseudo_log(x, 0.5)),
 scale(pseudo_log(x, 2)),
 scale(pseudo_log(x, 0.05))
 )


plot(x, y[, 2], type = "l", ylab = "f(x)")
lines(x, y[, 1], col = "red")
lines(x, y[, 3], type = "l", lty = 2)
lines(x, y[, 4], type = "l", lty = 3)
lines(x, y[, 5], type = "l", lty = 4)
legend(
 "bottomright",
 lty = c(1, 1, 2, 3, 4),
 col = c("red", "black", "black", "black", "black"),
 legend = c("log", "sigma=1", "sigma=0.5", "sigma=2", "sigma=0.05")
)

Figure S20. Logarithmic and pseudo-log transformations of 10x using several values for the hyperparameter sigma of the pseudo-log transformation.
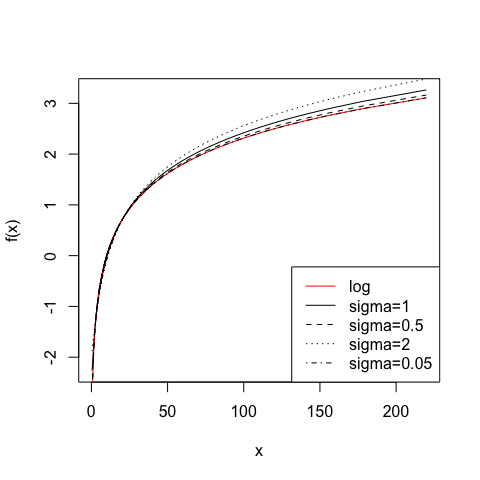


Finally, we apply the pseudo-logarithmic transformation to the original normal deviates. We learn that a higher value for the parameter $\sigma$ makes the distribution ‘slimmer’ while a lower value makes it ‘fatter’, and it is even possible to induce bimodality with low values of sigma:

z <- qnorm(p, mean = 0, sd = 1)
hist(z)
hist(pseudo_log(z, sigma = 1))
hist(pseudo_log(z, sigma = 2))
hist(pseudo_log(z, sigma = 0.5))
hist(pseudo_log(z, sigma = 0.05))

Figure S21. Histograms of a normally distributed variable x and of several pseudo-log transformations of x.

| 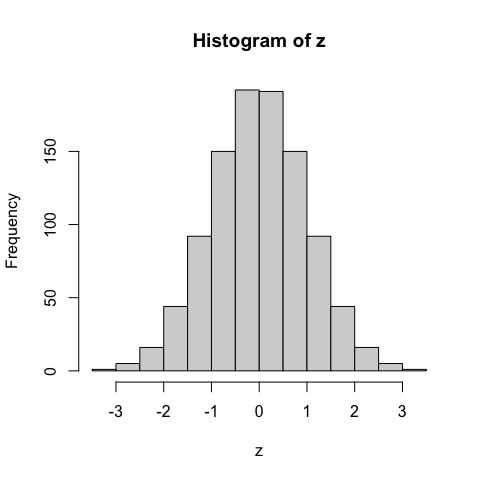 | 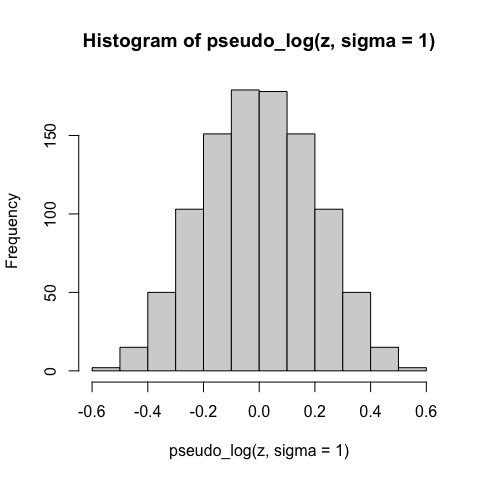 | 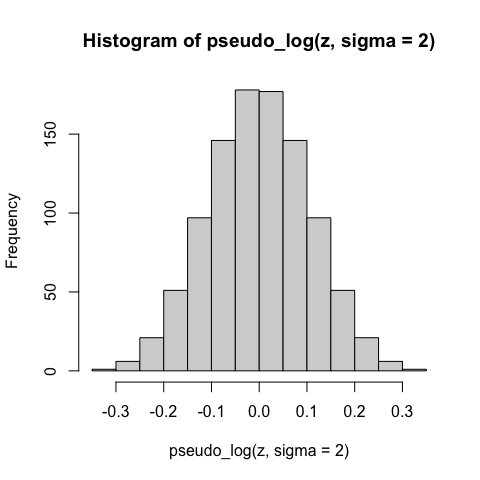 |
| --- | --- | --- |

| 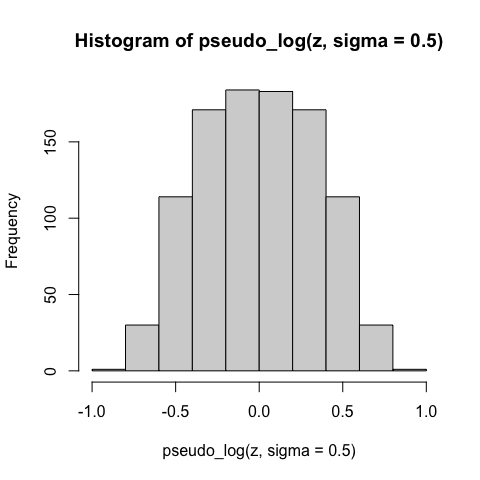 | 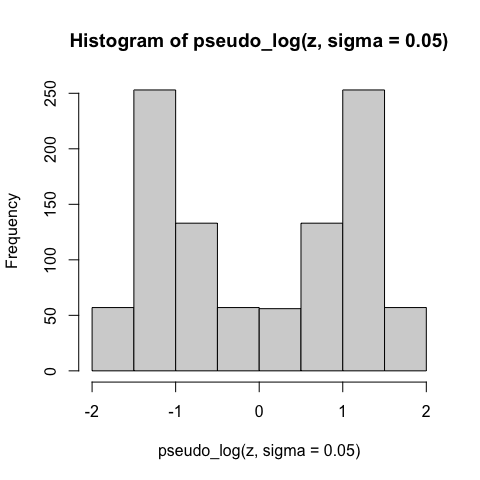 |
| --- | --- |

## A.3 Finding a parameter that best achieves normality

Any test statistic for testing normality could be chosen to find a suitable value of $\sigma$ that induces normality into the transformed values. Here we use the (Pearson) correlation coefficient to compare the empirical distribution with normal deviates.

### A.3.1 Deviates from log normal distribution

We simulate from a shifted log normal distribution and evaluate the value of $\sigma$ that optimizes agreement with a normal distribution:

# #| layout-ncol: 2 #Remark. did not render nicely in docx (GH)

x <- sort(exp(rnorm(1000) + 3))

hist(x)

Figure S22. Histogram of a simulated variable x drawn from a shifted log-normal distribution.
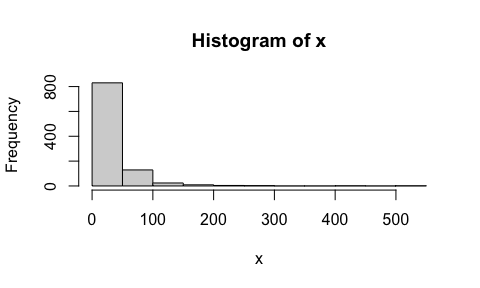


sigmas <- 2 ** seq(-10, 10, 1)
origcor <- cor(qnorm((1:length(x) - 0.5) / length(x)), x)

ncorx <-
 sapply(sigmas, function(X)
 cor(qnorm((1:length(
 x
 ) - 0.5) / length(x)), pseudo_log(x, X)))

cat("Optimal sigma: ")

Optimal sigma:

(optsigma <- sigmas[ncorx == max(ncorx)])

[1] 0.0009765625

plot(log2(sigmas), ncorx, ylab = "Correlation with normal deviates", ylim =
 c(0, 1))
points(log2(sigmas)[ncorx == max(ncorx)], max(ncorx), pch = 19)
abline(h = origcor)
legend(
 "bottomright",
 lty = c(1, NA, NA),
 pch = c(NA, 1, 19),
 legend = c("Original", "Pseudo-log", "Pseudo-log with optimal sigma")
)
box()

Figure S23. Correlation of pseudo-log transformation of x with normal deviates by sigma, the hyperparameter of the pseudo-log transformation.


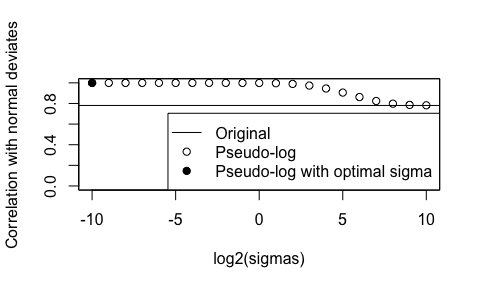


hist(pseudo_log(x, optsigma))

Figure S24. Histogram of the pseudo-log transformed x at the optimal value of sigma.


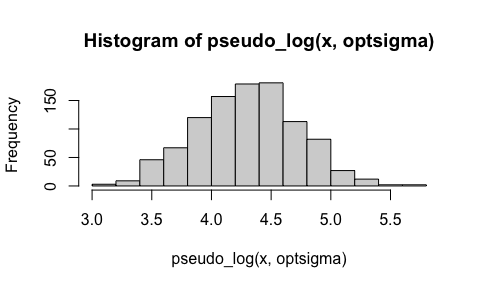


### A.3.2 Deviates from exponential distribution

Also with an exponential distribution, the pseudo-logarithm may achieve a better agreement with a normal:

x <- sort(exp(rnorm(1000) + 3))

hist(x)

Figure S25. Histogram of a simulated variable x following an exponential distribution.


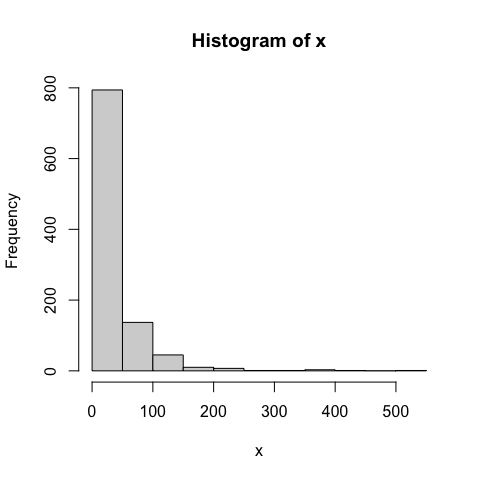


sigmas <- 2 ** seq(-10, 10, 1)
origcor <- cor(qnorm((1:length(x) - 0.5) / length(x)), x)

ncorx <-
 sapply(sigmas, function(X)
 cor(qnorm((1:length(
 x
 ) - 0.5) / length(x)), pseudo_log(x, X)))

cat("Optimal sigma: ")

Optimal sigma:

(optsigma <- sigmas[ncorx == max(ncorx)])

[1] 0.125

plot(log2(sigmas), ncorx, ylab = "Correlation with normal deviates", ylim =
 c(0, 1))
points(log2(sigmas)[ncorx == max(ncorx)], max(ncorx), pch = 19)
abline(h = origcor)
legend(
 "bottomright",
 lty = c(1, NA, NA),
 pch = c(NA, 1, 19),
 legend = c("Original", "Pseudo-log", "Pseudo-log with optimal sigma")
)
box()

Figure S26. Correlation of pseudo-log transformation of x with normal deviates by sigma, the hyperparameter of the pseudo-log transformation.
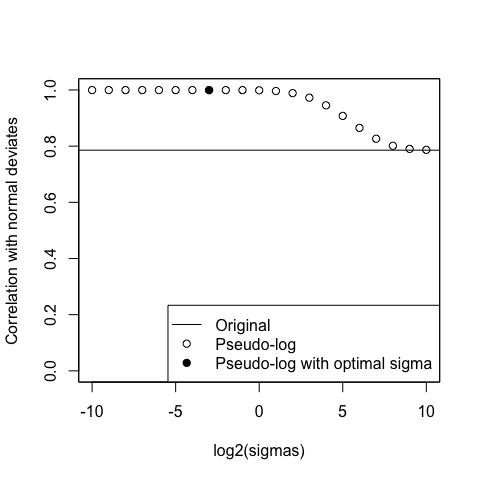


hist(pseudo_log(x, optsigma))

Figure S27. Histogram of the pseudo-log transformed x at the optimal value of sigma.


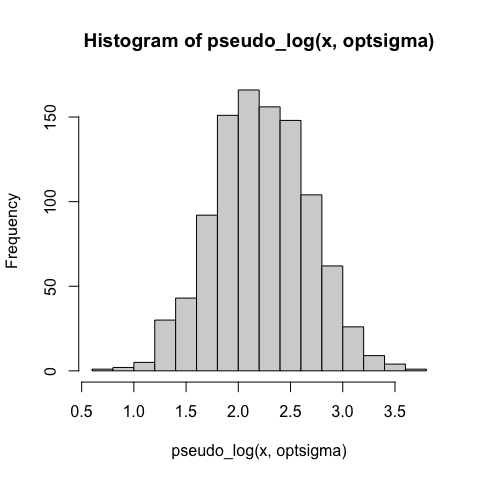


### A.3.3 Deviates from mixture distribution

Now we mix a normal, lognormal and exponential distribution:

x1 <- scale(rnorm(1000))
x2 <- scale(rexp(1000))
x3 <- scale(exp(rnorm(1000)))

p1 <- rbinom(1000, 1, 0.33)
p2 <- rbinom(1000, 1, 0.5)

x <- p1 * x1 + (1 - p1) * (p2 * x2 + (1 - p2) * x3)
x <- x - min(x)
x <- sort(x)

hist(x)

Figure S28. Histogram of a simulated variable x, drawn from a mixture of a normal, lognormal and exponential distribution.


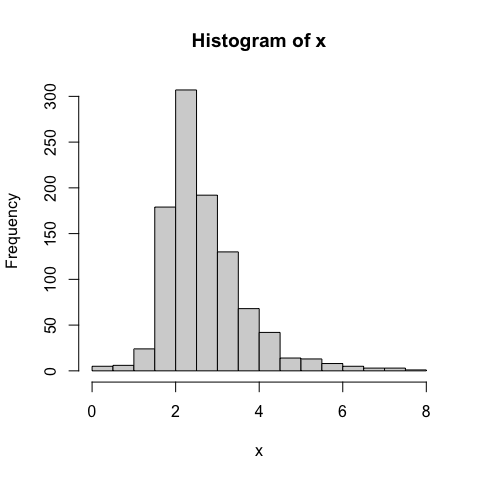


sigmas <- 2 ** seq(-10, 10, 1)
origcor <- cor(qnorm((1:length(x) - 0.5) / length(x)), x)

ncorx <-
 sapply(sigmas, function(X)
 cor(qnorm((1:length(
 x
 ) - 0.5) / length(x)), pseudo_log(x, X)))

cat("Optimal sigma: ")

Optimal sigma:

(optsigma <- sigmas[ncorx == max(ncorx)])

[1] 1

plot(log2(sigmas), ncorx, ylab = "Correlation with normal deviates", ylim =
 c(0, 1))
points(log2(sigmas)[ncorx == max(ncorx)], max(ncorx), pch = 19)
abline(h = origcor)
legend(
 "bottomright",
 lty = c(1, NA, NA),
 pch = c(NA, 1, 19),
 legend = c("Original", "Pseudo-log", "Pseudo-log with optimal sigma")
)
box()

Figure S29. Correlation of pseudo-log transformation of x with normal deviates by sigma, the hyperparameter of the pseudo-log transformation


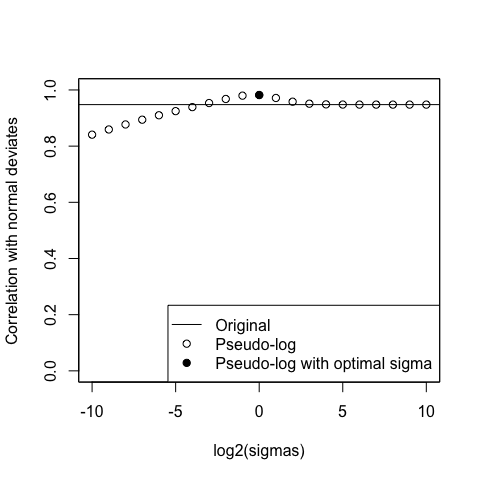


hist(pseudo_log(x, optsigma))

Figure S30. Histogram of the pseudo-log transformed x at the optimal value of sigma.


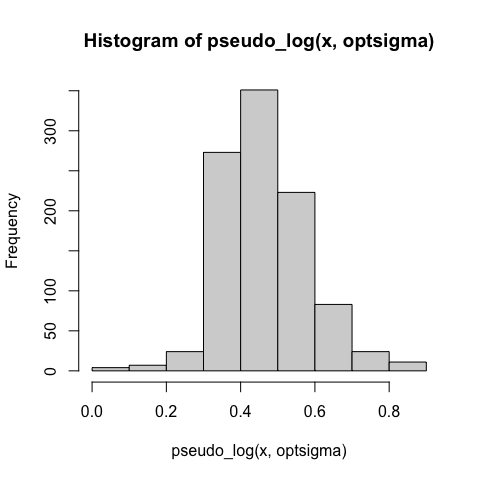


### A.3.4 Normal deviates

With simulated normal deviates, the pseudo-logarithm cannot improve the already perfect normality.

x <- sort(rnorm(1000))

hist(x)

Figure S31. Histogram of a simulated variable x, drawn from a standard normal distribution


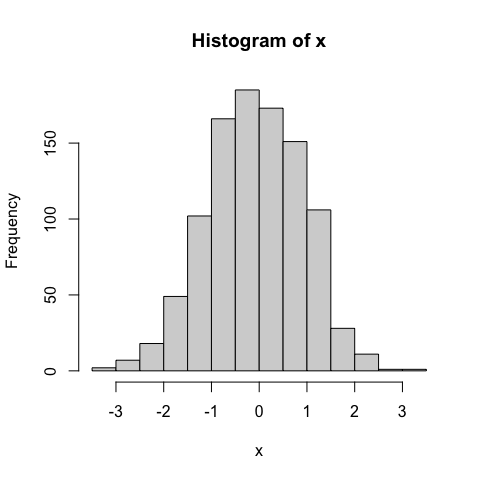


sigmas <- 2 ** seq(-10, 10, 1)
origcor <- cor(qnorm((1:length(x) - 0.5) / length(x)), x)

ncorx <-
 sapply(sigmas, function(X)
 cor(qnorm((1:length(
 x
 ) - 0.5) / length(x)), pseudo_log(x, X)))

cat("Optimal sigma: ")

Optimal sigma:

(optsigma <- sigmas[ncorx == max(ncorx)])

[1] 1024

plot(log2(sigmas), ncorx, ylab = "Correlation with normal deviates", ylim =
 c(0, 1))
points(log2(sigmas)[ncorx == max(ncorx)], max(ncorx), pch = 19)
legend(
 "bottomright",
 lty = c(1, NA, NA),
 pch = c(NA, 1, 19),
 legend = c("Original", "Pseudo-log", "Pseudo-log with optimal sigma")
)
abline(h = origcor)
box()

Figure S32. Correlation of pseudo-log transformation of x with normal deviates by sigma,
the hyperparameter of the pseudo-log transformation.


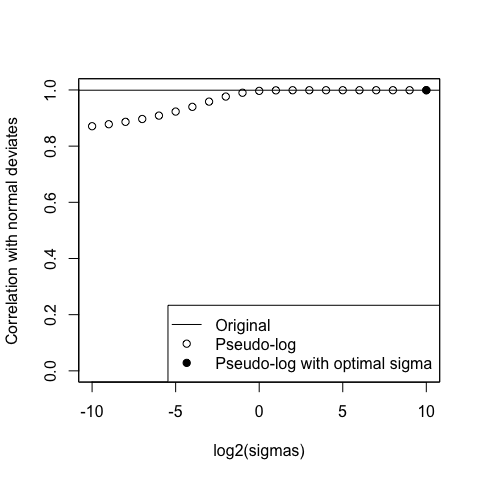


hist(pseudo_log(x, optsigma))

Figure S33. Histogram of the pseudo-log transformed x at the optimal value of sigma.


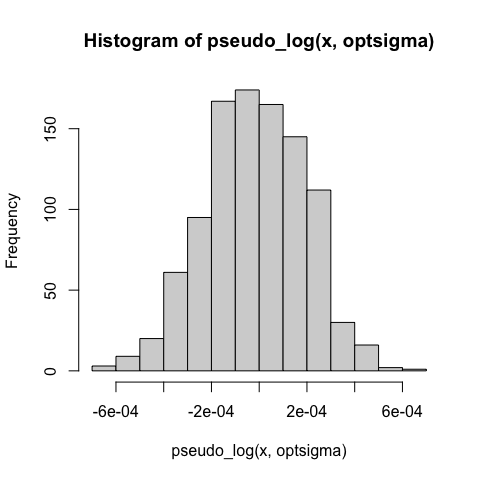


# Appendix B — Supplementary Example

We now perform an example research project on the final **analysis ready data** which is stored at data/analysis.

## B.1 Overview

In the following examples we use the Bacteremia data with complete observations regarding the key predictors PLT, BUN, NEU, AGE, CREA_T, WBC_noNEU_T, which represent 93.9% of the dataset **w.r.t key predictors**. We will fit a global logistic regression model with the outcome **BACTEREMIAN** (i.e. presence of bacteremia) and the key predictors as covariates. We will use pseudo-log transformations as suggested in the IDA. Within the model, all key predictors will be transformed by fractional polynomials of order 1 (df = 2).

The aim of the examples is to showcase how decisions derived from IDA influence the results of the fitted model.

### B.1.1 Global Model

The global model will be fitted by the *mfp* function. If not indicated otherwise, we will use the fp-transformations of the key predictors determined in global model in all consecutive models. For all models we report McFaddens’s R² and the AUC, i.e. the area under the ROC curve, and boxplots comparing “**BACTEREMIAN**” predictions with outcomes.

To put the results of the global model into context, we will first review the histograms of the original and the transformed predictors (Figure S32).

Figure S34: Histograms of key predictors of the global model.


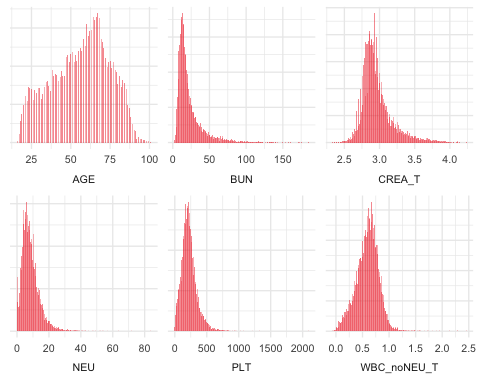


### B.1.2 Model Summary

The global model is specified as print(global_formula, quote = TRUE). The model is fit to the complete cases data on the **transformed** scale stored in the data set data_model_trans.

Report the global model fit.

Table S15: Fractional polynomial logistic regression of bacteremia status on key predictors, including pseudo-log transformed predictors for WBC_noNEU and CREA ("global model").

| term | estimate | std.error | statistic | p.value |
| --- | --- | --- | --- | --- |
| (Intercept) | -2.698 | 0.588 | -4.59 | 4.46 × 10^-6 |
| I((WBC_noNEU_T + 0.1)^0.5) | -5.223 | 0.255 | -20.51 | 1.85 × 10^-93 |
| I(((NEU + 0.1)/10)^0.5) | 1.525 | 0.117 | 13.09 | 4.00 × 10^-39 |
| I((AGE/100)^1) | 1.689 | 0.199 | 8.48 | 2.17 × 10^-17 |
| I(CREA_T^1) | 0.712 | 0.198 | 3.59 | 3.24 × 10^-4 |
| I(((PLT + 1)/100)^1) | -0.082 | 0.032 | -2.57 | 1.03 × 10^-2 |
| I((BUN/10)^1) | 0.010 | 0.024 | 0.43 | 6.65 × 10^-1 |

Plot predictions by outcome and report the model AUC (Figure S33).

Figure S35: Boxplots of predictions from global model by outcome status.

| \| 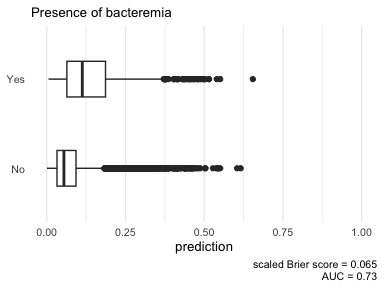  Global model - including covariates on the transformed psuedo log scale \| \| --- \| |
| --- | --- |

### B.1.3 Functional forms of global model

We now take a look at the functional forms of the covariates in the global model, which are determined by the fp algorithm. Besides scaling factors, only for WBC_noNEU_T the fp algorithm chose a non-linear transformation (note the ‘^0.5’ in the term column). This means all other covariates enter the model in a linear fashion. In the following effect plots, each variable is adjusted to the median of the other variables in the model.

Figure S36. Estimated functional forms of predictors in the global model. (a) PLT, (b) BUN, (c) NEU), (d) AGE, (e) CREA_T, (f) WBC_noNEU_T.

| \| \| 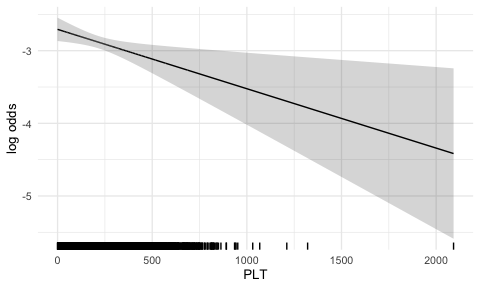  (a) \| \| --- \| \| \| 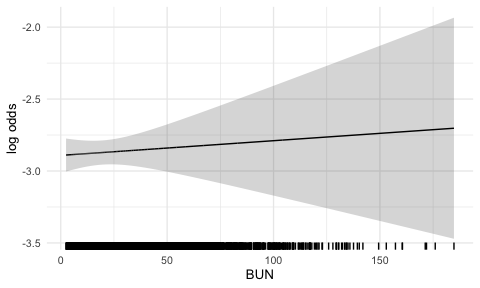  (b) \| \| --- \| \| \| --- \| --- \| --- \| --- \|  \| \| 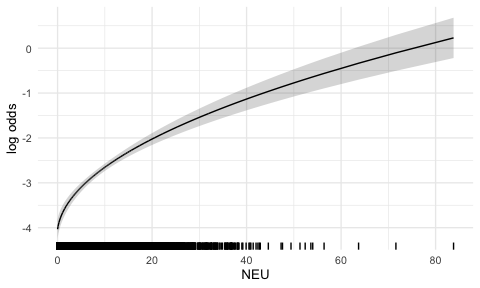  (c) \| \| --- \| \| \| 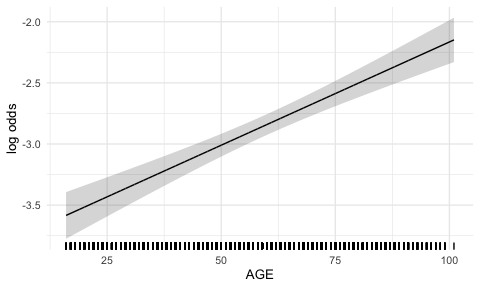  (d) \| \| --- \| \| \| --- \| --- \| --- \| --- \|  \| \| 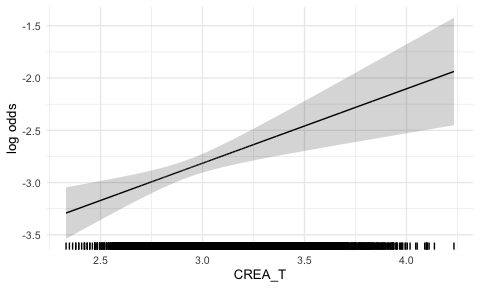  (e) \| \| --- \| \| \| 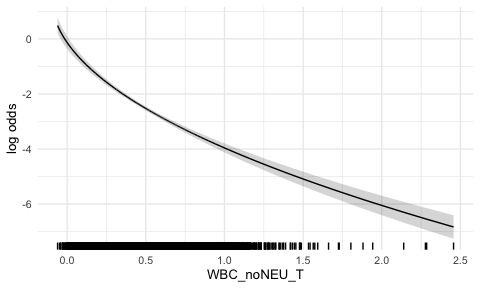  (f) \| \| --- \| \| \| --- \| --- \| --- \| --- \| |
| --- | --- | --- | --- | --- | --- | --- | --- | --- | --- | --- | --- | --- |

##

## B.2 Example 1: to transform or not to transform

Only for one out of the six key predictors did the fp algorithm chose a non-linear transformation. But out of those six variables, four were pseudo-log transformed before entering the model. In the first example we want to compare the global model to a model using the key predictors on their original scale (Table S18).

Table S16: Global model without pseudo-log tranformations

| term | estimate | std.error | statistic | p.value |
| --- | --- | --- | --- | --- |
| (Intercept) | -3.18 | 0.28 | -11.17 | 5.48 × 10^-29 |
| log((WBC_noNEU + 0.2)) | -1.25 | 0.06 | -20.22 | 5.92 × 10^-91 |
| I(((NEU + 0.1)/10)^0.5) | 1.52 | 0.12 | 12.99 | 1.34 × 10^-38 |
| I((AGE/100)^1) | 1.64 | 0.20 | 8.26 | 1.51 × 10^-16 |
| I(((PLT + 1)/100)^1) | -0.08 | 0.03 | -2.53 | 1.13 × 10^-2 |
| I((BUN/10)^1) | 0.02 | 0.02 | 0.68 | 5.00 × 10^-1 |
| I(CREA^-0.5) | -0.77 | 0.21 | -3.69 | 2.20 × 10^-4 |

Note the different fp-transformation arising when the key predictors are not pseudo-log transformed. On the original scale, three covariates instead of one now enter the model via a non-linear fp-transformation. This suggests that a transformation prior to the regression model ‘outsources’ the need for transformations within the model. Now let us compare the model performances.

Figure S37. Predictions by outcome status from the global model with key predictors entered with their original or pseudo-log transformed scales.

| \| 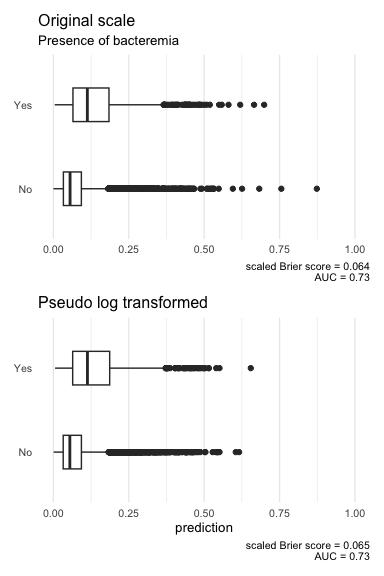 \| \| --- \| |
| --- | --- |

With regards to McFadden’s R² and the AUC, the differences between the two approaches is marginal.

Next we will compare the differences of the functional forms in the two models for those covariates where a pseudo-log transformation was suggested in IDA. We will look at the log odds for bacteremia by each covariate on the original and the transformed scale, and compare the global model using the original and the pseudo-log transformed covariates. Each variable is adjusted for the median of all other variables used.

Figure S38. Comparison of functional forms when predictors CREA and WBC_noNEU entered the global model either with their original scales or with their pseudo-log scales. The two functional forms resulting from the two models are shown on the original scale (left column) or on the pseudo-log scale (right column).

| 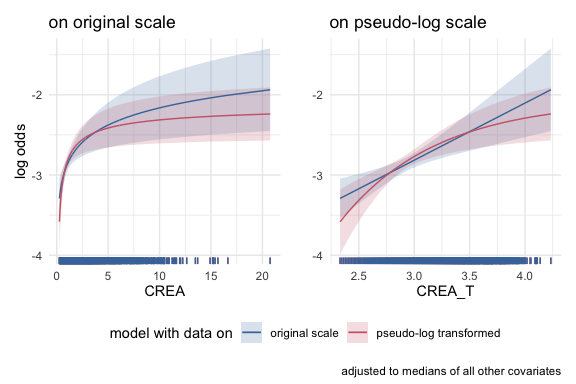 |
| --- |

| 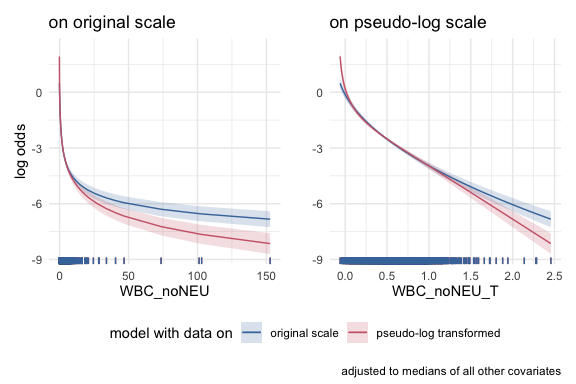 |
| --- |

|  |
| --- |

## B.3 Example 2: Interpretation of regression coefficient ‘size’

As a result of IDA, it turned out that the key predictor WBC_noNEU should be transformed for further analyses because of its extreme skewness. As a consequence, the original variable WBC_noNEU and the pseudo-logged variable WBC_noNEU_T are now on two very different scales (Figure S37).

Figure S39. Histograms of the original predictor WBC_noNEU (top) and the pseudo-log transformed predictor WBC_noNEU_T (bottom).


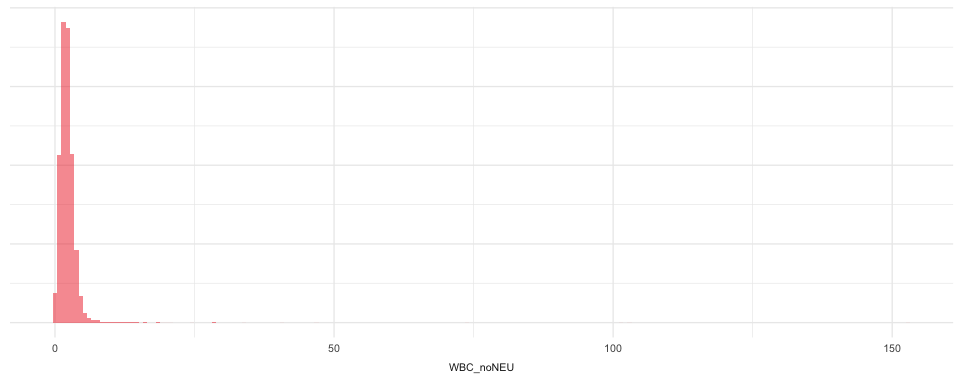


To illustrate that the scale of the original variable determines the size of the regression coefficient, let us consider two logistic regression models with WBC_noNEU and t_WBC_noNEU as single covariate, respectively.

Table S17: Regression coefficients in simple logistic regression models using the original predictor WBC_noNEU or the pseudo-log transformed predictor WBC_noNEU_T.

| term | estimate | std.error | statistic |
| --- | --- | --- | --- |
| WBC_noNEU | -0.557 | 0.035 | -15.731 |
| WBC_noNEU_T | -2.888 | 0.152 | -18.950 |

Even if the model with transformed WBC has a slightly better prediction performance as revealed by the summary plots (Figure S38), this is not the reason for the difference. The estimates -0.56 and -2.89 indicate the difference in log odds for the outcome when the ‘term’ variable differs by 1 unit, but cannot be compared directly. A $1$ unit difference is only a small step on the original scale, where WBC_noNEU covers values from -0.15 up to 152.74. In comparison, WBC_noNEU_T lies between -0.06 up to 2.46, so a difference of $1$ unit cover almost half the range of the variable.

Figure S40. Predictions from simple logistic regression models using the original predictor WBC_noNEU (top) or the pseudo-log transformed predictor WBC_noNEU_T (bottom).

## B.4 Example 3: the support of a model determines what it can explain

Next we compare the global model to a model were for an important variable, in our case we chose age, the variable support is reduced to the central 50% of the data (i.e. data within the 25% and 75% quantiles), and for comparison we also generate a scenario where we randomly sample 50% of the data.

When evaluating the apparent predictive performance in terms of the AUC and the scaled Brier score, we see that the model fitted on the participants with age between the first and third quartiles performs worse than the global model and the model where 50% were randomly sampled.

Table S18. Apparent predictive performance of global model ("complete"), model based on the subjects with age between the 25th and 75th percentiles of age ("central 50% of age distribution") and of a model based on randomly sampled 50% of the original cohort ("randomly sampled 50%").

| data | AUC | scaled Brier score |
| --- | --- | --- |
| complete | 0.731 | 0.065 |
| central 50% of age distribution | 0.723 | 0.053 |
| randomly sampled 50% | 0.738 | 0.065 |

Plot the model predictions based on different missing data scenarios

Figure S41. Predictions from the three models described in Table S21.

|  |
| --- |

##

## B.5 Example 4: the limits of multiple imputation

To show the effect of multiple imputation if the number of missing values is high, we construct a dataset with 50% artificially generated missing values in one variable. First, recall the output of the complete model, relying on the Bacteremia data with complete cases regarding the key predictors.

Table S19: Fractional polynomial logistic regression model (global model) of bacteremia status with key predictors

| term | estimate | std.error | statistic | p.value |
| --- | --- | --- | --- | --- |
| (Intercept) | -2.70 | 0.59 | -4.59 | 4.46 × 10^-6 |
| I((WBC_noNEU_T + 0.1)^0.5) | -5.22 | 0.25 | -20.51 | 1.85 × 10^-93 |
| I(((NEU + 0.1)/10)^0.5) | 1.52 | 0.12 | 13.09 | 4.00 × 10^-39 |
| I((AGE/100)^1) | 1.69 | 0.20 | 8.48 | 2.17 × 10^-17 |
| I(CREA_T^1) | 0.71 | 0.20 | 3.59 | 3.24 × 10^-4 |
| I(((PLT + 1)/100)^1) | -0.08 | 0.03 | -2.57 | 1.03 × 10^-2 |
| I((BUN/10)^1) | 0.01 | 0.02 | 0.43 | 6.65 × 10^-1 |

Creatinine (‘CREA’) is significant at a level that might not survive substantial missingness. We thus create a dataset were we artificially introduce 50% missing creatinine values, missing completely at random.

Next we fit a ‘complete case’ model in the case of missing creatinine data, using the fp-transformations from the global model.

Now we impute the missing creatinine data using MICE with 50 imputations, fit the model using the fp-transformations from the global model and pool the results.

We now can compare the outputs of the complete model, the complete model with missing data (i.e. only half of the original complete data is used), and the imputed model (Table S23).

Table S20: Comparison of the models based on no missing data, with complete cases, and with imputed data

| term | model | estimate | std.error | statistic | p.value |
| --- | --- | --- | --- | --- | --- |
| (Intercept) | missing, complete cases | -3.896 | 0.822 | -4.741 | 2.12 × 10^-6 |
|  | missing, imputed | -3.899 | 0.790 | -4.934 | 1.67 × 10^-6 |
|  | no missing data | -2.698 | 0.588 | -4.589 | 4.46 × 10^-6 |
| I(((NEU + 0.1)/10)^0.5) | missing, complete cases | 1.611 | 0.166 | 9.695 | 3.16 × 10^-22 |
|  | missing, imputed | 1.544 | 0.117 | 13.176 | 2.11 × 10^-39 |
|  | no missing data | 1.525 | 0.117 | 13.085 | 4.00 × 10^-39 |
| I(((PLT + 1)/100)^1) | missing, complete cases | -0.070 | 0.046 | -1.532 | 1.25 × 10^-1 |
|  | missing, imputed | -0.078 | 0.032 | -2.431 | 1.51 × 10^-2 |
|  | no missing data | -0.082 | 0.032 | -2.565 | 1.03 × 10^-2 |
| I((AGE/100)^1) | missing, complete cases | 1.897 | 0.287 | 6.601 | 4.09 × 10^-11 |
|  | missing, imputed | 1.669 | 0.199 | 8.365 | 6.61 × 10^-17 |
|  | no missing data | 1.689 | 0.199 | 8.484 | 2.17 × 10^-17 |
| I((BUN/10)^1) | missing, complete cases | -0.024 | 0.033 | -0.717 | 4.74 × 10^-1 |
|  | missing, imputed | -0.028 | 0.030 | -0.930 | 3.53 × 10^-1 |
|  | no missing data | 0.010 | 0.024 | 0.434 | 6.65 × 10^-1 |
| I((WBC_noNEU_T + 0.1)^0.5) | missing, complete cases | -5.372 | 0.365 | -14.703 | 6.18 × 10^-49 |
|  | missing, imputed | -5.275 | 0.257 | -20.555 | 1.91 × 10^-92 |
|  | no missing data | -5.223 | 0.255 | -20.507 | 1.85 × 10^-93 |
| I(CREA_T^1) | missing, complete cases | 1.093 | 0.275 | 3.969 | 7.22 × 10^-5 |
|  | missing, imputed | 1.153 | 0.277 | 4.154 | 5.09 × 10^-5 |
|  | no missing data | 0.712 | 0.198 | 3.595 | 3.24 × 10^-4 |

When half of the data is missing, the standard errors of the coefficients increase by a factor of approximately $\sqrt{2}$. Multiple imputation is able to recreate standard errors very close to those of the model with no missing data in most variables, but not in the one that was being imputed, namely creatinine. Its relative importance in the model is higher in the complete cases model than in the imputed model.

## B.6 Example 5: Plot of functional form should focus on areas with high density

The functional forms have wide confidence intervals when the data is sparse. In presentations of the effects, plots of the functional forms should focus on areas with high density. In this analysis, PLT was very sparse above ~800 [UNITS], which is reflected in a large confidence interval for high PLT values. In the effect plot PLT values could be limited to values <800 [UNITS].

Figure S42. Predictions by bacteremia status of the model with the key predictors where the predictors were considered only with linear functional forms.

|  |
| --- |

Figure S43. Functional forms of WBC_noNEU_T (top) and PLT (bottom) in the linear and MFP models. Left column: full range of predictors. Right column: plot is restricted to area of highest density.

| WBC_noNEU comparison |
| --- |

| PLT comparison |
| --- |

#

# Appendix C — Source data

The **source** data available in this repository (**DC 2019-0054**) can be located in the data-raw folder. For persistance, the data set is also published on [Zenodo](https://doi.org/10.5281/zenodo.7554815) with the following doi: https://doi.org/10.5281/zenodo.7554815.

The following sections provide a short overview of the data dictionary which accompanies the source data, and a short description of the data.

## C.1 Data dictionary

The data dictionary provides an overview of the collected data. First, we read and display the data dictionary below providing an overview of the collected measurements.

The variable name and label are displayed alongside the measurement scale and units as well as remarks and relevant study information from_paper.

Table S21. Data dictionary of bacteremia data set.

| variable | label | scale_of_measurement | units |
| --- | --- | --- | --- |
| ID | Patient Identification | nominal | 1-14691 |
| SEX | Patient sex | nominal | 1=male, 2=female |
| AGE | Patient Age | continuous | years |
| MCV | Mean corpuscular volume | continuous | pg |
| HGB | Haemoglobin | continuous | G/L |
| HCT | Haematocrit | continuous | % |
| PLT | Blood platelets | continuous | G/L |
| MCH | Mean corpuscular hemoglobin | continuous | fl |
| MCHC | Mean corpuscular hemoglobin concentration | continuous | g/dl |
| RDW | Red blood cell distribution width | continuous | % |
| MPV | Mean platelet volume | continuous | fl |
| LYM | Lymphocytes | continuous | G/L |
| MONO | Monocytes | continuous | G/L |
| EOS | Eosinophils | continuous | G/L |
| BASO | Basophiles | continuous | G/L |
| NT | Normotest | continuous | % |
| APTT | Activated partial thromboplastin time | continuous | sec |
| FIB | Fibrinogen | continuous | mg/dl |
| SODIUM | Sodium | continuous | mmol/L |
| POTASS | Potassium | continuous | mmol/L |
| CA | Calcium | continuous | mmol/L |
| PHOS | Phosphate | continuous | mmol/L |
| MG | Magnesium | continuous | mmol/L |
| CREA | Creatinine | continuous | mg/dl |
| BUN | Blood urea nitrogen | continuous | mg/dl |
| HS | Uric acid | continuous | mg/dl |
| GBIL | Bilirubin | continuous | mg/dl |
| TP | Total protein | continuous | G/L |
| ALB | Albumin | continuous | G/L |
| AMY | Amylase | continuous | U/L |
| PAMY | Pancreas amylase | continuous | U/L |
| LIP | Lipases | continuous | U/L |
| CHE | Cholinesterase | continuous | kU/L |

Table S21 (continued). Data dictionary of bacteremia data set.

| variable | label | scale_of_measurement | units |
| --- | --- | --- | --- |
| AP | Alkaline phosphatase | continuous | U/L |
| ASAT | Aspartate transaminase | continuous | U/L |
| ALAT | Alanin transaminase | continuous | U/L |
| GGT | Gamma-glutamyl transpeptidase | continuous | G/L |
| LDH | Lactate dehydrogenase | continuous | U/L |
| CK | Creatinine kinases | continuous | U/L |
| GLU | Glucoses | continuous | mg/dl |
| TRIG | Triclyceride | continuous | mg/dl |
| CHOL | Cholesterol | continuous | mg/dl |
| CRP | C-reactive protein | continuous | mg/dl |
| BASOR | Basophile ratio | continuous | % |
| EOSR | Eosinophil ratio | continuous | % |
| LYMR | Lymphocyte ratio | continuous | % (mg/dl) |
| MONOR | Monocyte ratio | continuous | % |
| NEU | Neutrophiles | continuous | G/L |
| NEUR | Neutrophile ratio | continuous | % |
| PDW | Platelet distribution width | continuous | % |
| RBC | Red blood count | continuous | T/L |
| WBC | White blood count | continuous | G/L |
| BloodCulture | Blood culture result for bacteremia | nominal | no, yes |

We do not display all observations measured as it is too wide and long to fit reasonably in to the report. However, we refer you to the [Zenodo page](https://doi.org/10.5281/zenodo.7554815) for an interactive overview of the source data.

# Appendix D — Results of IDA: Missing values Appendix

## D.1 M2: Variable missingness for remaining predictors

Table S22: Summary of missing values for remaining predictors

| Predictor | Description | Missing (count) | Missing (%) |
| --- | --- | --- | --- |
| PAMY | Pancreas amylase (U/L) | 7114 | 48.4 |
| TRIG | Triclyceride (mg/dl) | 5061 | 34.4 |
| CHOL | Cholesterol (mg/dl) | 5045 | 34.3 |
| GLU | Glucoses (mg/dl) | 4192 | 28.5 |
| AMY | Amylase (U/L) | 3913 | 26.6 |
| LIP | Lipases (U/L) | 3699 | 25.2 |
| HS | Uric acid (mg/dl) | 3061 | 20.8 |
| APTT | Activated partial thromboplastin time (sec) | 2549 | 17.4 |
| NT | Normotest (%) | 2467 | 16.8 |
| CHE | Cholinesterase (kU/L) | 2447 | 16.7 |
| CK | Creatinine kinases (U/L) | 2080 | 14.2 |
| MG | Magnesium (mmol/L) | 1869 | 12.7 |
| LDH | Lactate dehydrogenase (U/L) | 1714 | 11.7 |
| ALB | Albumin (G/L) | 1676 | 11.4 |
| TP | Total protein (G/L) | 1583 | 10.8 |
| GBIL | Bilirubin (mg/dl) | 1441 | 9.8 |
| AP | Alkaline phosphatase (U/L) | 1400 | 9.5 |
| SODIUM | Sodium (mmol/L) | 1282 | 8.7 |
| CA | Calcium (mmol/L) | 1276 | 8.7 |
| PHOS | Phosphate (mmol/L) | 1242 | 8.5 |
| PDW | Platelet distribution width (%) | 1102 | 7.5 |
| BASOR | Basophile ratio (%) | 732 | 5.0 |
| EOSR | Eosinophil ratio (%) | 732 | 5.0 |
| LYMR | Lymphocyte ratio (% (mg/dl)) | 732 | 5.0 |
| MONOR | Monocyte ratio (%) | 732 | 5.0 |
| NEUR | Neutrophile ratio (%) | 732 | 5.0 |
| MPV | Mean platelet volume (fl) | 702 | 4.8 |
| RBC | Red blood count (T/L) | 461 | 3.1 |
| LYM | Lymphocytes (G/L) | 262 | 1.8 |
| MONO | Monocytes (G/L) | 246 | 1.7 |
| BASO | Basophiles (G/L) | 146 | 1.0 |
| EOS | Eosinophils (G/L) | 135 | 0.9 |
| RDW | Red blood cell distribution width (%) | 56 | 0.4 |
| MCV | Mean corpuscular volume (pg) | 42 | 0.3 |
| HCT | Haematocrit (%) | 42 | 0.3 |
| MCH | Mean corpuscular hemoglobin (fl) | 42 | 0.3 |
| MCHC | Mean corpuscular hemoglobin concentration (g/dl) | 42 | 0.3 |
| HGB | Haemoglobin (G/L) | 41 | 0.3 |

# Appendix E — Univariate distribution checks

This section reports a series of univariate summary checks of the bacteremia dataset.

## E.1 U2: Descriptive summaries

### E.1.1 U2: Remaining predictors

We present a visual summary in Figure S44.

Figure S44: Histograms of remaining predictors

|  |  |  |
| --- | --- | --- |

|  |  |  |
| --- | --- | --- |

|  |  |  |
| --- | --- | --- |

|  |  |  |
| --- | --- | --- |

|  |  |  |
| --- | --- | --- |

Figure S44 (continued). Histograms of remaining predictors.

|  |  |  |
| --- | --- | --- |

|  |  |  |
| --- | --- | --- |

|  |  |  |
| --- | --- | --- |

|  |  |  |
| --- | --- | --- |

|  |  |  |
| --- | --- | --- |

|  |  |  |
| --- | --- | --- |

|  |  |  |
| --- | --- | --- |

Figure S44 (continued). Histograms of remaining predictors.

|  |  |
| --- | --- |

Figure S45. Combined numerical and graphical summaries of remaining predictors.

Figure S45 (continued). Combined numerical and graphical summaries of remaining predictors.

Figure S45 (continued). Combined numerical and graphical summaries of remaining predictors.

Figure S45 (continued). Combined numerical and graphical summaries of remaining predictors.

Figure S45 (continued). Combined numerical and graphical summaries of remaining predictors.

Figure S45 (continued). Combined numerical and graphical summaries of remaining predictors.

### E.1.2 Full descriptive summaries

For U2, we present only a limited number of statistics are presented for brevity. However, a full set of descriptive summaries are available according to the specifications in the IDA plan as a data set. The summary statistics can be viewed and analysed in the following directory data/results/U2-descriptive-stats.csv.

# Appendix F — Multivariate analyses

First load the required packages and data. Note:, from the univariate analyses the analysis data set for the lab parameters has been updated to include transformed variables. Therefore, we load the second iteration of the data data/IDA/ADLB_02.rds.

## F.1 V1: Association with structural variables

Figure S46. Association of remaining predictors with structural variables age and sex.

|  |  |  |
| --- | --- | --- |

|  |  |  |
| --- | --- | --- |

|  |  |  |
| --- | --- | --- |

|  |  |  |
| --- | --- | --- |

Figure S46 (continued). Association of remaining predictors with structural variables age and sex.

|  |  |  |
| --- | --- | --- |

|  |  |  |
| --- | --- | --- |

|  |  |  |
| --- | --- | --- |

|  |  |  |
| --- | --- | --- |

|  |  |  |
| --- | --- | --- |

|  |  |  |
| --- | --- | --- |

Figure S46 (continued). Association of remaining predictors with structural variables age and sex.

|  |  |  |
| --- | --- | --- |

|  |  |  |
| --- | --- | --- |

|  |
| --- |

## F.2 VE3: Redundancy

### F.2.1 Linear model

VIF for the all predictor model.

Table S23. Variance inflation factors and multiple R-squared of all predictors in a model with all predictors. The available sample size is 3979 (27.08 %).

| Parameter code | Variance inflation factor | Multiple R-squared |
| --- | --- | --- |
| SEX | 1.3 | 0.25 |
| MCV | 129.5 | 0.99 |
| HGB | 254.3 | 1.00 |
| HCT | 249.4 | 1.00 |
| PLT | 1.9 | 0.48 |
| MCH | 179.4 | 0.99 |
| MCHC | 47.5 | 0.98 |
| RDW | 1.9 | 0.47 |
| MPV | 9.2 | 0.89 |
| MONO | 4.3 | 0.77 |
| BASO | 2.6 | 0.62 |
| NT | 1.5 | 0.32 |
| APTT | 1.2 | 0.18 |
| FIB | 2.9 | 0.66 |
| SODIUM | 1.4 | 0.30 |
| POTASS | 1.4 | 0.29 |
| CA | 2.1 | 0.52 |
| PHOS | 1.6 | 0.36 |
| MG | 1.3 | 0.23 |
| BUN | 3.2 | 0.69 |
| HS | 1.8 | 0.43 |
| TP | 4.5 | 0.78 |
| ALB | 6.3 | 0.84 |
| CHE | 2.8 | 0.65 |
| GLU | 1.3 | 0.20 |
| TRIG | 1.4 | 0.29 |
| CHOL | 1.9 | 0.47 |
| CRP | 2.8 | 0.65 |

Table S23 (continued). Variance inflation factors and multiple R-squared of all predictors in a model with all predictors. The available sample size is 3979 (27.08 %).

| Parameter code | Variance inflation factor | Multiple R-squared |
| --- | --- | --- |
| EOSR | 47.3 | 0.98 |
| LYMR | 1,321.2 | 1.00 |
| MONOR | 321.4 | 1.00 |
| NEU | 8.9 | 0.89 |
| NEUR | 2,179.1 | 1.00 |
| PDW | 9.1 | 0.89 |
| RBC | 40.1 | 0.98 |
| AGE | 1.5 | 0.31 |
| ALAT_T | 3.9 | 0.74 |
| AMY_T | 2.7 | 0.63 |
| AP_T | 2.3 | 0.57 |
| ASAT_T | 6.6 | 0.85 |
| CK_T | 2.2 | 0.55 |
| CREA_T | 2.9 | 0.65 |
| EOS_T | 3.5 | 0.71 |
| GBIL_T | 1.6 | 0.38 |
| GGT_T | 2.6 | 0.62 |
| LDH_T | 2.2 | 0.55 |
| LIP_T | 2.2 | 0.54 |
| LYM_T | 4.5 | 0.78 |
| PAMY_T | 3.4 | 0.71 |
| WBC_T | 15.7 | 0.94 |
